# Evaluation of the Chemical Profile by Paper Spray Mass Spectrometry of *Eugenia uniflora* Pulp, Peel, Seeds, and Jelly

**DOI:** 10.1002/jms.5173

**Published:** 2025-09-16

**Authors:** Viviane D. M. Silva, Laiza A. Nogueira, Ana Luiza C. C. Ramos, Bruna V. Nunes, Claudio A. Lopes, Mauro R. Silva, Eric M. Garcia, Hosane Aparecida Taroco, Ricardo Manuel S. de Boavida Ferreira, Rodinei Augusti, Julio Onesio‐Ferreira Melo

**Affiliations:** ^1^ Departamento de Ciências Exatas e Biológicas Campus Sete Lagoas, Universidade Federal de São João del‐Rei Sete Lagoas Minas Gerais Brazil; ^2^ Departamento de Alimentos, Faculdade de Farmácia Campus Belo Horizonte, Universidade Federal de Minas Gerais Belo Horizonte Minas Gerais Brazil; ^3^ LEAF‐Instituto Superior de Agronomia Universidade de Lisboa Lisbon Portugal; ^4^ Departamento de Bioquímica e Imunologia Universidade Federal de Minas Gerais Belo Horizonte Minas Gerais Brazil; ^5^ Departamento de Química Campus Belo Horizonte, Universidade Federal de Minas Gerais Belo Horizonte Minas Gerais Brazil

**Keywords:** Brazilian cherry, conservation, fingerprints, phytochemicals

## Abstract

Pitangueira ( *Eugenia uniflora* ) is a fruit tree found in the Cerrado, Caatinga, and Atlantic Forest Brazilian biomes. Due to its intense and characteristic aroma and flavor, its fruits (Brazilian cherry) can be consumed raw and as an ingredient in jellies, giving it better use and a more excellent shelf life. The objective of this work was to evaluate the chemical profile of chemical compounds in the pulp, peel, seed, and jelly prepared with the pulp and peel of Brazilian cherry. The characterization of the chemical profile was performed by a fast and simple method: paper spray ionization method coupled with mass spectrometry in positive and negative ionization modes. Forty‐six compounds were tentatively identified in the negative mode and 15 in the positive mode, belonging to the classes of phenolic acids, organic acids, fatty acids, hydroxycinnamic acids, sugars, betalains, carotenoids, flavonoids, and tannins. Characterizing these native Brazilian fruits and their products may increase interest in their use, highlighting the potential of Brazilian cherry as a source of nutrients and bioactive compounds. Therefore, developing technological processes for better use and conservation of fruits and, at the same time, identifying the phytochemical compounds present in the product is a way of valuing and preserving our Brazilian biodiversity.

**Abbreviations**
HCA: hierarchical clustering analysis
HPLC‐ESI‐MS/MS: high‐performance liquid chromatography coupled to electrospray ionization tandem mass spectrometry
PCA: principal component analysis
PS‐MS: paper spray mass spectrometry
PS(−)‐MS: paper spray mass spectrometry in negative ionization mode
PS(+)‐MS: paper spray mass spectrometry in positive ionization mode

## 1 Introduction

The pitangueira ( *Eugenia uniflora* L.), a fruit tree from the Myrtaceae family, comes from the subtropical climate region, is native to the Brazilian Atlantic Forest, and is also found in the Cerrado and Caatinga biomes. Its fruit, the pitanga or Brazilian cherry, has high levels of carbohydrates, vitamins A, C, B12, and B3, carotenoids [1, 2, 3, 4], and other antioxidant compounds, such as anthocyanins (in the case of purple fruits) [5].

According to the genotype, the Brazilian cherry epicarp begins with a green color, turning yellow, orange, red, dark red, and purple. This characteristic results from the accumulation of pigments during maturation and accentuated physical–chemical changes that give the fruit an attractive flavor and aroma for consumption [6, 7]. Three different types of Brazilian cherry are reported depending on the color of the fruit's epicarp: the orange Brazilian cherry, the red Brazilian cherry, and the purple Brazilian cherry [7, 8]. The red variety is most affluent in carotenoids, such as *β*‐carotene and lycopene, and in the flavonoids myricetin, kaempferol, and quercetin [2]. In addition to color contributions, anthocyanin and carotenoid pigments help prevent anti‐inflammatory and anti‐carcinogenic diseases and reduce reactive oxygen species, scavenging free radicals and highlighting their antioxidant action [5]. Furthermore, these fruits are reported to have antimicrobial, antipyretic, antirheumatic, hypocholesterolemic, antidiabetic, antihypertensive, and antinociceptive activities [3, 9, 10].

Furthermore, compared with other vegetal species, Brazilian cherry has lower lipid concentrations and low caloric content [11]. In sensory terms, due to its sweet and sour flavor and its high pulp yield, Brazilian cherry can be consumed fresh, processed as frozen pulp and jellies, or added to ice creams, alcoholic beverages, and confectionery [3, 12].

The use of fruits in developing new products seeks to help improve the sensory perceptions of confectionery products, reducing the amount of sugar added to the food and, consequently, nutritionally and sensorially enriching the final product. Furthermore, using by‐products aligns with fully utilizing food and minimizing losses and waste [13]. The Brazilian cherry comprises 77% peel and pulp, 23% seeds [2], and around 85% water [14]. In this way, using Brazilian cherry peel in the production of jams is a viable alternative to add economic value to the fruit, minimizing losses that may occur during the commercialization of the fresh fruit.

The chemical profile of the fruit is influenced by the species, variety, climate, ripeness, region, harvest time, storage, and extract preparation method [2]. Aiming at a broader characterization of Brazilian cherry, paper spray mass spectrometry (PS‐MS) allows fingerprints to be obtained in wide ranges of masses, where sample preparation is minimal or unnecessary, and the ionization step is ultrafast [15, 16]. The identification of several chemical compounds present in the peel and pulp can add value to the fruit's production chain, as it aims at the applicability of a by‐product in the form of jellies, which could be associated with improved quality of life due to its high antioxidant capacity from Brazilian cherry. Therefore, the objective of the work was to develop a jelly using the peel and pulp of red Brazilian cherry and identify the possible compounds present in this product as well as in the parts of the Brazilian cherry by PS‐MS in order to verify whether the processing to prepare the jelly affects this characterization.

## 2 Materials and Methods

### 2.1 Vegetal Material

The red Brazilian cherry samples were collected in Sete Lagoas, MG, in October 2022. The fruits were washed in running water to eliminate dirt and disinfected in a 200 ppm sodium hypochlorite solution for 15 min with subsequent rinsing. They were then packaged in polyethylene bags, identified, and stored in a freezer (−18°C).

### 2.2 Jelly Development

The Brazilian cherry was thawed, and the peels, pulp, and seeds were manually separated. To develop the jellies, 60 g of a mixture of Brazilian cherry peel and pulp, 40 g of crystal sugar, and 0.2 g of xanthan gum were used. Initially, sugar was added to the sample, and this mixture was subjected to cooking on a hot plate, with continuous manual stirring, for approximately 4 min. Then, xanthan gum was added, keeping the mixture under heating with continuous manual stirring for 2 min. Finally, the jelly was packaged in a glass jar, closed with a metal lid, and stored at room temperature for 10 days [17].

### 2.3 Chemical Profile by PSMS

The extracts were prepared from the fruit parts, pulp, and peel homogenized together, and the seeds were crushed in a batch analytical mill (IKA A 11 basic, Staufen, Germany) to evaluate the chemical profile. The jelly and the cherry parts were weighed (1 g) separately on an analytical balance, and 8 mL of methanol (HPLC grade) was added to a Falcon‐type flask. These mixtures were vortexed for 30 s and left at rest for 1 h at room temperature [18].

The chemical profile of the extracts was determined according to the methodology described by Ramos et al. [18] using an LCQ Fleet mass spectrometer (ThermoScientific, USA) coupled with an ionization source for paper pulverizing. For this, chromatographic paper in the shape of an equilateral triangle (1.5 cm) was used, placed at a distance of 0.5 cm from the spectrometer entrance in a metal connector. A total of 2 μL of the extract and 40 μL of methanol were applied to the paper, reading in triplicate in positive and negative ionization modes.

The instrumental conditions of the analyses were: source voltage at 4.5 kV for the positive mode and 3.5 kV for the negative mode; capillary voltage of 40 V; transfer tube temperature of 275°C; tube lens voltage of 120 V; mass range from 100 to 1000 *m/z*. To identify the compounds, the charge mass ratios from literature data were compared with the instrumental signals obtained and subsequent fragmentation with collision energies of 15–30 eV [16, 17, 19, 20, 21, 22].

### 2.4 Statistical Analysis

Principal component analysis (PCA) was performed using the central average of the data by MatLab software Version R2021a (9.10.0.1602886) (Mathworks, Natick, MA, USA) and the PLS Toolbox extension Version 8.9 (Eigenvectors Research, Manson, WA, USA).

## 3 Results

Sixty‐one compounds were tentatively identified, 15 in the positive mode and 46 in the negative mode, belonging to different classes, such as phenolic acids, organic acids, fatty acids, hydroxycinnamic acids, sugars, betalains, carotenoids, flavonoids, and tannins. In general, in the Brazilian cherry pulp and peel sample, 56 compounds (92%) were found in pulp and peel jelly, 48 compounds (79%), and in the Brazilian cherry seed, 25 compounds (41%).

### 3.1 Chemical Profile in Positive Ionization Mode

Examples of spectra of PS‐MS analysis in the positive mode (PS(+)‐MS) of Brazilian cherry pulp and peel, seed, and jelly are illustrated in Figure 1. Product ion mass spectrum is shown in Figures S1–S15.

**FIGURE 1:**
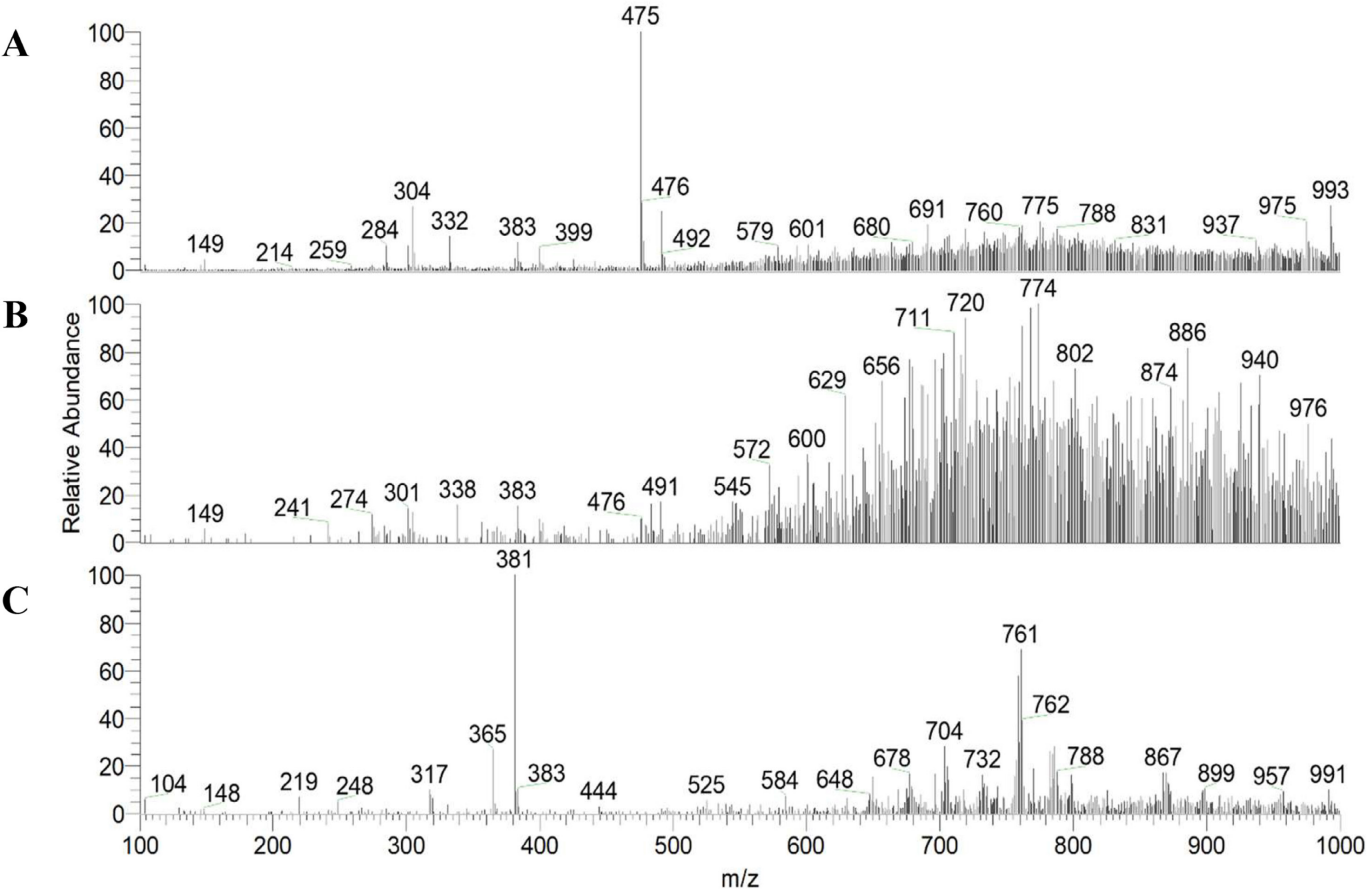
Representation of PS(+)‐MS of a sample of pulp and peel (A), seed (B), and jelly (C) of Brazilian cherry.

Table 1 presents the possible compounds identified in the extracts of Brazilian cherry samples through ionization in positive mode. It was observed that 15 compounds were suggested in the pulp and peel fraction; of these, nine compounds were present in the jelly and eight in the seed. Only five compounds were common to these three samples: (9Z)‐violaxanthin/neoxanthin (*m/z* 601), luteolin malonyldihexoside (*m/z* 703), apigenin hexoside caffeate (*m/z* 723), kaempferol diacetyl dicoumaroylhexoside (*m/z* 825), and kaempferol dihexoside (*m/z* 869).

**TABLE 1:** Compounds tentatively identified in Brazilian cherry seed (S), pulp and peel (P + P), and peel and pulp jelly (J) in positive PS(+)‐MS mode.

Compound	Precursor ion (*m/z*)	Fragments (*m/z*)	Chemical class	Samples			References
				S	P + P	J	
Diosmetin	301	286	Flavonoid	—	X	—	[23]
Fertaric acid	365	203, 347	Phenylpropanoid	—	X	X	[24]
Sucrose		185, 203	Sugar				[23, 25]
Sucrose/hexose	381	201, 219	Sugar	—	X	X	[16, 20]
Caffeoyl‐hexose		201, 219, 335, 363	Phenylpropanoid				[24, 26]
Dihydrosynapic acid	475	457	Phenylpropanoid	X	X	—	[20, 23]
Malvidin‐*O*‐galactoside	493	331	Flavonoid	—	X	—	[27]
6′‐*O*‐malonyl‐2‐descarboxy‐isobetanin	593	507, 549	Betalains	—	X	X	[23, 28]
(9Z)‐Violaxanthin/neoxanthin	601	221, 565, 583	Carotenoid	X	X	X	[29]
Rutin	611	303	Flavonoid	—	X	—	[30]
Luteolin malonyldihexoside	703	523, 541, 671, 685	Flavonoid	X	X	X	[24]
Kaempferol hydroxypropionylhexoside hexoside	721	477, 533, 559, 689	Flavonoid	X	X	—	[24]
Apigenin hexoside caffeate	723	543, 561, 691	Flavonoid	X	X	X	[24]
Kaempferol hexosyl acetylhexosyl hexoside	783	649, 737, 765	Flavonoid	—	X	X	[26]
Kaempferol diacetyl dicoumaroylhexoside	825	713, 779, 807	Flavonoid	X	X	X	[24]
Kaempferol dihexoside	869	707, 823	Flavonoid	X	X	X	[24]
Myricetin coumaryl dihexoside	957	911	Flavonoid	X	X	—	[24]

*Note:* X: detected; —: not detected.

**FIGURE 2:**
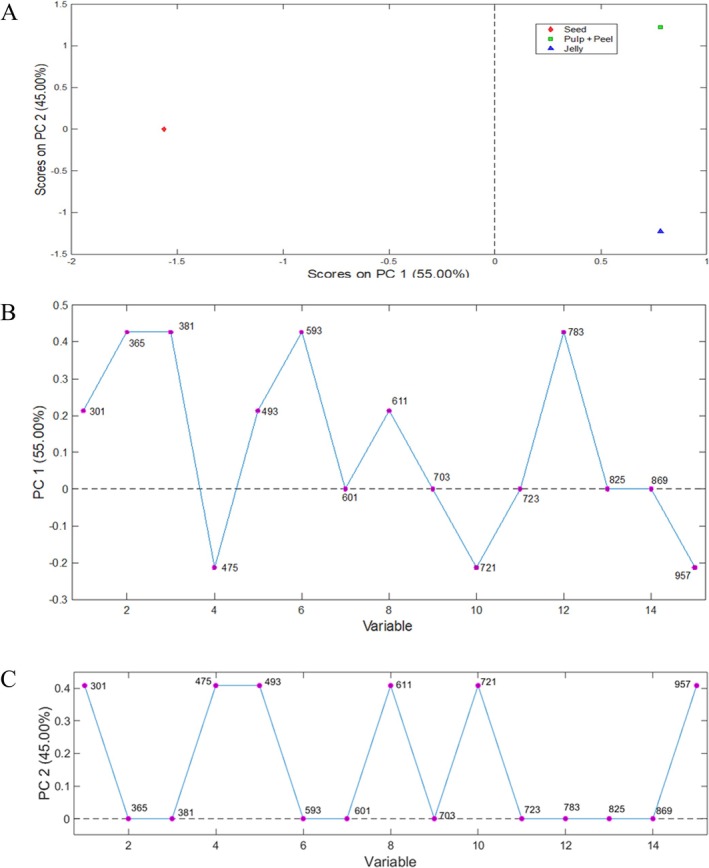
Representation of *E. uniflora* extracts in the first and second dimensions through principal component analysis (PCA) (A) and of the main ions responsible for sample separation in PC 1 (B) and PC 2 (C) in positive mode.

Matrices X (3 × 15) were obtained from fingerprints of the samples analyzed in the positive ionization modes. PCA was used by selecting two principal components (Figure 2A), whose model explained 100% of the total variance. With PCA, it was possible to group the samples according to their variances of characteristics and similarities. Thus, it can be observed in Figure 2A that PC 1 (55.0% of the total variance) allowed the separation between the seed samples (negative scores) and the jelly and pulp + peel samples (positive scores). It was expected that pulp + peel and jam would be in the same group because the jam was prepared with these two parts of the pitanga.

Figure 2B,C shows the ions responsible for the separation of each group in PC 1 and PC 2, respectively. In PC 1 (Figure 2B), the ions responsible for separating the pulp + peel and jelly samples were *m/z* 301 (diosmetin), 365 (fertaric acid or sucrose), 381 (sucrose/hexose), 493 (malvidin‐*O*‐galactoside), 593 (6′‐*O*‐malonyl‐2‐descarboxy‐isobetanin), 611 (rutin), and 783 (kaempferol hexosyl acetylhexosyl hexoside). While the differentiation of the seeds is due to the compounds: dihydrosynapic acid (*m/z* 475), kaempferol hydroxypropionylhexoside hexoside (*m/z* 721), and myricetin coumaryl dihexoside (*m/z* 957) (negative scores). In PC 2 (Figure 2C), the separation between the jelly and the pulp + peel sample is due to the ions of *m/z* 301 (diosmetin), 475 (dihydrosynapic acid), 493 (malvidin‐*O*‐galactoside), 611 (rutin), 721 (kaempferol hydroxypropionylhexoside hexoside), and 957 (myricetin coumaryl dihexoside).

### 3.2 Chemical Profile in Negative Ionization Mode

Examples of spectra of PS‐MS analysis in the negative mode (PS(−)‐MS) of Brazilian cherry pulp and peel, seed, and jelly are illustrated in Figure 3. Examples of product ion mass spectra are shown in Figures S16–S61.

**FIGURE 3:**
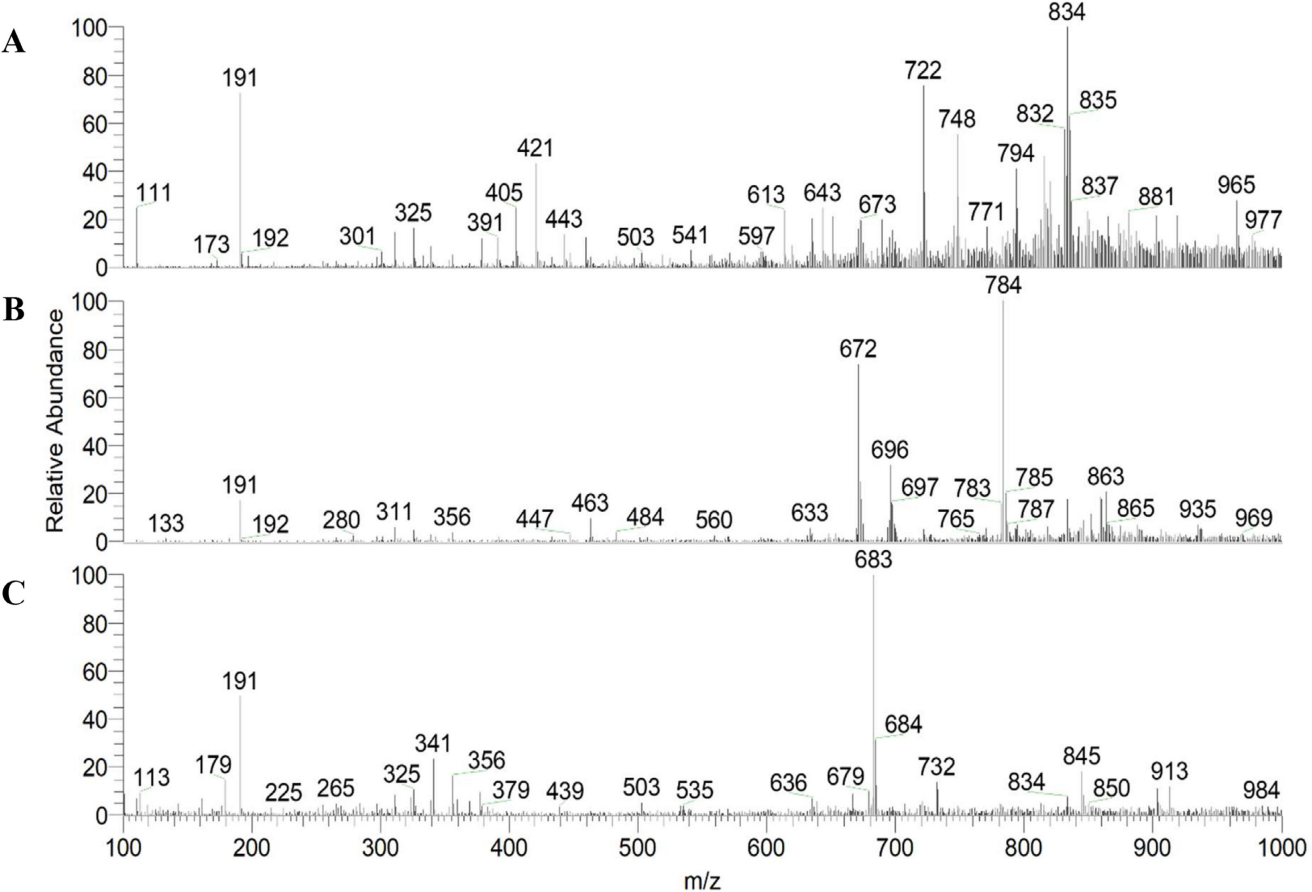
Representation of PS(−)‐MS of a sample of pulp and peel (A), seed (B), and jelly (C) of Brazilian cherry.

Regarding the result in negative mode, presented in Table 2, most of the identified compounds were found in the pulp and peel (41 compounds) and in the jelly (39 compounds), while 17 compounds were found in the seed. As in the positive mode, flavonoids presented the highest number of representatives.

**TABLE 2:** Compounds tentatively identified in Brazilian cherry seed (S), pulp and peel (P + P), and peel and pulp jelly (J) in negative PS(−)‐MS mode.

Compound	Precursor ion (*m/z*)	Fragments (*m/z*)	Chemical class	Samples			References
				S	P + P	J	
Coniferaldehyde	177	162	Phenylpropanoid	—	X	X	[31]
Glucose/hexose ([M − H]^−^)	179	71, 89, 179	Sugar	—	X	X	[16, 25]
Citric acid	191	85, 87, 111, 129, 173	Organic acids	X	X	X	[23, 32, 33]
Quinic acid		85, 109, 111, 113, 127, 131, 153, 171, 173					[8, 20, 32, 33, 34]
Ferulic acid	193	134	Phenylpropanoid	—	X	X	[31]
Caffeic acid	215	153, 179, 197	Phenylpropanoid	—	X	X	[26]
Eugenitin	255	179, 211, 237	Chomones	—	X	X	[26]
Tryhydroxyflavone	269	225	Flavonoid	—	X	X	[25]
Naringenin	271	151	Flavonoid	—	X	X	[10]
Oleic acid	281	237	Fatty acids	—	X	X	[25]
Stearic acid	283	265	Fatty acids	—	X	X	[25]
Ellagic acid	301	175, 229, 257	Polyphenol	X	—	—	[14, 33]
Quercetin		151, 163, 179, 185, 193, 229, 257, 273	Flavonoid				[8, 29, 32, 33, 34]
(Epi)gallocatechin	305	261, 137	Flavonoid	—	X	X	[23, 35]
Ferulol malic acid	309	291	Phenylpropanoid	—	X	X	[23]
Eicosanoic acid	311	293	Fatty acids	X	X	X	[20, 25]
*p*‐Coumaric acid hexoside	325	119, 145, 163, 183	Phenylpropanoid	X	X	X	[16, 20, 23]
Oxo‐dihydroxy‐octadecenoic acid isomer	327	183, 291, 309	Fatty acids	—	X	X	[25]
Caffeoyl‐2‐hydroxyethane‐1,1,2‐tricarboxylic acid	339	295	Phenylpropanoid	—	X	X	[23]
Caffeoyl‐hexoside	341	161, 179, 185, 297	Phenylpropanoid	—	X	X	[25, 29, 36]
5‐*O*‐galloylquinic acid	343	191	Polyphenol	X	—	—	[37]
Chlorogenic acid	353	123, 183, 191	Flavonoid	—	X	X	[29, 31, 35]
Pinoresinol	357	311	Lignan	—	X	X	[20]
Syringic acid hexoside	359	153, 197	Polyphenol	—	X	X	[16, 20]
Coumaric acid derivative	369	205, 223	Phenylpropanoid	—	X	X	[36]
Hexose or sucrose ([2Hex + H_2_O‐H]^−^ or [Suc + 2 H_2_O‐H]^−^)	377	215, 341	Sugar	—	X	X	[16, 20, 25]
Feruloylquinic acid	383	191	Phenylpropanoid	—	X	X	[8]
Sucrose ([M + HCOO]^−^)	387	341	Sugar	X	X	X	[23]
Kaempferol pentoside	417	151, 169, 227, 255, 284, 285	Flavonoid	—	X	X	[10, 14]
Derivatives of caffeic acid	443	443	Phenylpropanoid	—	X	X	[23]
Kaempferol‐hexoside	447	255, 284, 285, 301	Flavonoid	X	—	—	[29, 36]
Quercitrin		151, 179, 255, 271, 300, 301					[10, 14, 32]
Quercetin‐*O*‐hexoside	463	151, 179, 255, 271, 300, 301, 419	Flavonoid	X	—	—	[23, 29, 32, 38, 39]
Myricetin‐rhamnoside		151, 169, 179, 271, 287, 301, 316, 317, 445					[3, 8, 10, 20, 34, 38]
2,3‐Di‐*O*‐galloyl‐D‐glucoside	483	331, 271, 169	Tannin	X	—	—	[10]
Rosmarinic acid rhamnoside	505	359	Phenylpropanoid	—	X	X	[40]
Cyclo lariciresinol hexoside	521	359	Lignans	—	X	X	[20, 25]
Tinosposide A	535	373, 517	Lignans	—	X	X	[23, 25]
Quercetin galloyl hexoside	615	301	Flavonoid	X	X	—	[3, 8, 9, 32]
Myricetin‐galloyl‐hexoside	631	613, 571, 499, 479, 271	Flavonoid	—	X	—	[3, 9, 29, 32, 35]
Strictinin	633	301, 481	Tannin	X	X	X	[35, 39]
Trigalloyl‐glucoside	635	465	Tannin	X	X	X	[10]
Di‐glucoside of di‐hydro‐myricetin	643	481	Flavonoid	—	X	X	[23]
Caffeic acid hexoside dimer	683	341	Tannin	—	X	X	[16]
Prodelphinidin B3–*O*‐gallate isomer	761	591	Tannin	—	X	X	[35]
Pedunculagin	783	765, 631, 613, 481, 451, 301, 275, 257	Tannin	X	X	X	[20, 36, 39]
Digalloyl‐HHDP‐hexose	785	301, 419, 633, 767	Tannin	X	X	X	[29]
Digalloyl‐HHDP‐glucose		300, 483, 633					[10]
Tellimagrandin I		301, 483, 615					[39]
Procyanidin B trimer	865	407	Tannin	X	X	X	[35]
Castalagin/vescalagin	933	631	Tannin	X	X	X	[35, 36, 39, 40]
Galloyl‐HHDP‐DHHDP‐hexoside	951	915	Tannin	X	X	X	[40]

*Note:* X: detected; —: not detected.

**FIGURE 4:**
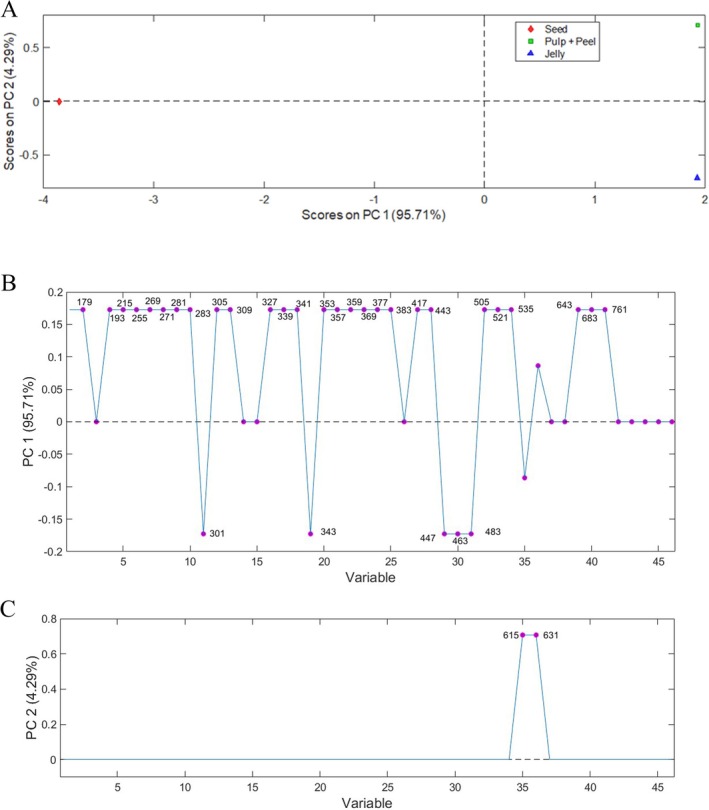
Representation of *E. uniflora* extracts in the first and second dimensions through principal component analysis (PCA) (A) and of the main ions responsible for sample separation in PC 1 (B) and PC 2 (C) in negative mode.

In the PCA in the negative mode, the Matrix Y and Y (3 × 61) was obtained from the ions obtained in the negative ionization mode, selecting two principal components, whose model explained 100% of the total variance. PC 1 corresponded to 95.71% of the total variance and allowed the separation of the samples as occurred in the positive ionization mode (Figure 4A).

Figure 4B, in PC 1, highlights 28 ions responsible for differentiating the pulp + peel and jelly samples from those seed samples, most of which belong to the classes of phenylpropanoids and flavonoids. Exclusively in the seeds (negative scores), compounds with *m/z* 301 (ellagic acid or quercetin), 343 (5‐*O*‐galloylquinic acid), 447 (kaempferol‐hexoside or quercitrin), 463 (quercetin‐*O*‐hexoside or myricetin‐rhamnoside), and 483 (2,3‐di‐*O*‐galloyl‐*D*‐glucoside) are highlighted. In PC2 (Figure 4C), it was found that the compounds quercetin galloyl hexoside (*m/z* 615) and myricetin‐galloyl‐hexoside (*m/z* 631) were the only ones that differentiated the pulp + peel of the jelly sample, thus indicating that the processing performed to obtain the jelly was adequate, as it kept most of the compounds found in the pulp + peel also in the jelly.

**FIGURE 5:**
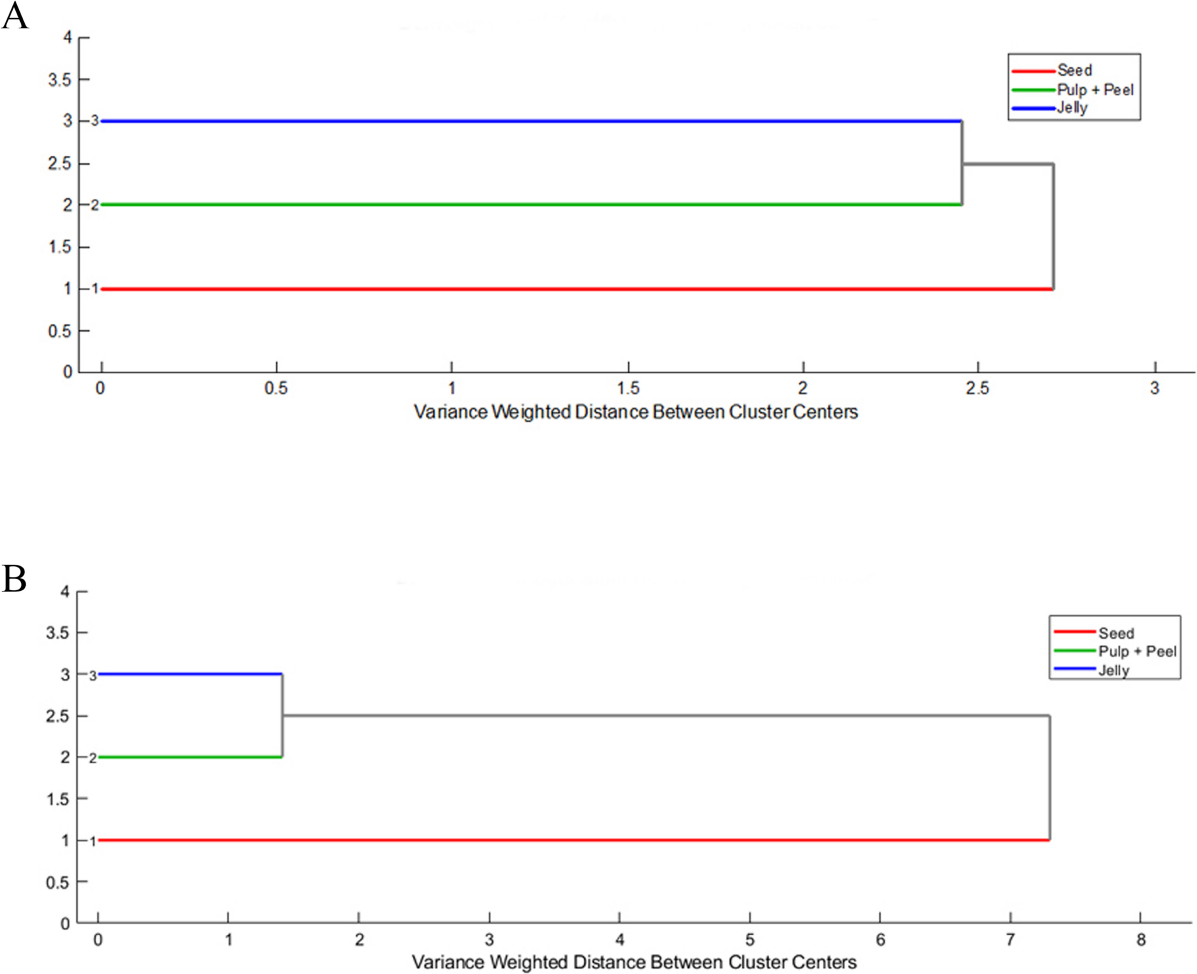
Dendrogram obtained from the analysis of hierarchical clusters (HCA) in the representation of *Eugenia uniflora* samples in positive mode (A) and negative mode (B).

Figure 5 shows the dendrogram generated by hierarchical clustering analysis (HCA), which, like the PCA, also showed the grouping of the analyzed samples, separating pulp + peel and jelly from the seed sample.

## 4 Discussion

### 4.1 Chemical Profile in Positive Ionization Mode

It was found that the processing of Brazilian cherry for the production of jelly reduced the amount of compounds compared with the pulp and peel sample. Heating during the jelly preparation may have caused the loss of these compounds. This can be observed with some glycoside compounds, in which glycosidic bonds may have been broken; for example, with the compounds at *m/z* 493, 611, 721, and 957 that were not identified in the jelly. However, there are still substances characteristic of fresh fruit. Zitha et al. [41] also found losses of phenolic compounds, such as rutin, catechin, and quercetin, in mangaba jelly compared with the mangaba pulp ( *Hancornia speciosa* ), due to the high temperatures used during processing. Therefore, for the samples analyzed, consuming Brazilian cherry pulp and peel in natura or in the form of jelly can bring health benefits due to compounds with bioactive properties, such as flavonoids.

Table 1 shows that most tentatively identified compounds are flavonoids (60%). Flavonoids represent one of the most important groups of phenolic compounds, as they are essential for plants and have antioxidant action, playing a fundamental role in disease prevention [42].

The peak at *m/z* 365 can be suggested to be fertaric acid or sucrose because fragments of both compounds were found in the Brazilian cherry pulp, peel, and jelly. Fertaric acid is a phenolic acid that has also been identified in other fruits of the same *Eugenia* species, such as in the pulp and peel of uvaia ( *Eugenia pyriformis* ) [26] and in the pulp and peel of araçá‐boi ( *Eugenia stipitata* ) [24].

Sucrose was identified in the literature as having signals at *m/z* 365 ([M + Na]^+^) and 381 ([2Hex + K − H_2_O]^+^) and is present in Brazilian cherry's pulp, peel, and jelly. This compound was also found in other fruits of the Myrtaceae family and their derived products, such as pulp [16], jelly [17], and ice cream [43] from the pulp of *Eugenia dysenterica* and in the pulp of *Myrciaria floribunda* [20].

Carotenoids are lipophilic natural pigments with antioxidant properties; therefore, they are necessary for our health and also of great interest to the food industry [44]. In this work, the presence of the carotenoid (9Z)‐violaxanthin or neoxanthin (*m/z* 601) was suggested in all samples analyzed, as well as it was also identified in the peel and pulp of Brazilian cherry and the mesocarp of *Caryocar brasiliense* in the study by Biazotto et al. [29].

The signal at *m/z* 611 was observed only in the mixture of Brazilian cherry pulp and peel and in the crude extract of *Eugenia brasiliensis* leaves in the study by Siebert et al. [30]. This ion was related to the flavonoid rutin, which demonstrated interesting antioxidant activity, probably related to the content of phenolics and flavonoids, using the HPLC‐ESI‐MS/MS analysis technique [30].

The ion with *m/z* 869 was identified in the three samples and suggested to be the flavonoid kaempferol dihexoside, based on its transitions *m/z* 869 → 707 and 823, which was also found by Araújo et al. [24] in the crude extract of the edible fraction and seeds of *Eugenia stipitata* (Myrtaceae).

### 4.2 Chemical Profile in Negative Ionization Mode

Only two glycosidic compounds identified in the pulp and peel (*m/z* 615 and 631) were not present in the jelly. Probably, heating during the preparation of the jam broke the glycosidic bond of these substances, which caused possible degradation.

The ion with *m/*z 191 can be recognized as citric acid, a natural source of organic acid, found in citrus fruits [23] or as quinic acid. These two compounds were present in the three samples analyzed. They were also observed in the seeds and leaves, respectively, of *Eugenia dysenterica* , also from the Myrtaceae family, by Justino et al. [32].

The compound at *m/z* 215 was identified in the pulp, peel, and jelly of Brazilian cherry as caffeic acid ([M + Cl]^−^), also found by Farias et al. [26] when evaluating the chemical profile of *Eugenia pyriformis* (Myrtaceae) seeds. This hydroxycinnamic acid has been reported to have potential antioxidant and anti‐inflammatory effects [45].

Four fatty acids have been suggested in pita Brazilian cherry pulp, peel, and jelly: oleic acid (*m/z* 281), stearic acid (*m/z* 283), eicosanoic acid (*m/z* 311, also present in Brazilian cherry seed), and oxo‐dihydroxy‐octadecenoic acid isomer (*m/z* 327). Mariano et al. [25] also identified all of these compounds in the pulp of *Eugenia klotzschiana* Berg.

The signal at *m/z* 301 was found only in the Brazilian cherry seed and can be suggested to be the flavonoid quercetin or the polyphenol ellagic acid. This last compound was also reported by Stafussa et al. [33] in the fruits of *Psidium guineenses* , *Campomanesia phaea* , *Myrciaria cauliflora* , and Brazilian cherry, who also observed it predominantly in the Brazilian cherry seed. In addition to these compounds, four other ions at *m/z* 343, 447, 463, and 483 were exclusively identified in the Brazilian cherry seed.

The signal *m/z* 343 was suggested to be 5‐*O*‐galloylquinic acid. This phenolic acid was identified by Garmus et al. [37] in Brazilian cherry leaf extracts using the UPLC‐ESI‐MS/MS technique. The presence of phenolic compounds in plants has been extensively studied, as they have pharmacological activities and inhibit lipid oxidation and the proliferation of fungi. Furthermore, they participate in processes responsible for various foods' color, astringency, and aroma [7, 14, 46].

Among the flavonoids tentatively identified in the samples, quercetin derivatives stand out, substances that provide beneficial effects to our health, such as antioxidant activity [23, 33, 35]. In the review study by Nogueira et al. [42], it was highlighted that quercetin and its derivatives were the most cited flavonoids in the researched fruits of the genus *Eugenia*.

The ion at *m/z* 463 can be suggested as the flavonoids quercetin‐*O*‐hexoside or myricetin‐rhamnoside. This last compound was reported by Celli, Pereira‐Netto, and Beta [38] as being the main flavonoid glycoside found in Brazilian cherry of the red variety in all stages of development, as well as in the green and yellow maturation stages of Brazilian cherry of the purple variety.

The tannin group suggested seven compounds with signals at *m/z* 359, 633, 783, 785, 865, 933, and 951, with the majority found in the three samples analyzed. Tannins are polyphenols produced from the secondary metabolism of plants, with the primary function of protection against pathogenic microorganisms and insects, among other external agents. Furthermore, they have diverse applications, such as in the tanning industry and the development of food supplements and medicines [47].

The compound at *m/z* 683 was identified as caffeic acid hexoside dimer, also found in the pulp of *Eugenia dysenterica* (Myrtaceae) by Silva et al. [16], who used the same technique to obtain fingerprints of this fruit.

Given these, Brazilian cherry's results, including its peel and seed fractions, which are generally not consumed, and Brazilian cherry jelly, are sources of compounds with potential functionality for our organism. Therefore, even with the processing of Brazilian cherry to produce jelly, this product presented compounds characteristic of the fruit *in natura*, indicating that heating was not as harmful and that this process can preserve the phytochemicals present in Brazilian cherry.

## Author Contributions

**Viviane D. M. Silva:** formal analysis, investigation, visualization, writing – review and editing. **Laiza A. Nogueira** and **Claudio A. Lopes:** formal analysis, writing original draft, investigation. **Bruna V. Nunes** and **Ana Luiza C. C. Ramos:** data curation, investigation, methodology. **Mauro R. Silva:** formal analysis, validation, writing – review and editing. **Eric M. Garcia:** writing – review and editing. **Hosane Aparecida Taroco:** writing – review and editing. **Ricardo Manuel S. de Boavida Ferreira:** methodology, resources. **Rodinei Augusti:** methodology, resources. **Julio Onesio‐Ferreira Melo:** conceptualization, funding acquisition, methodology, project administration, resources, supervision, writing – review and editing. All authors read and approved the submitted version..

## Conflicts of Interest

The authors declare no conflicts of interest.

## Supporting information


**Figure S1:** Product ion mass spectrum of the ion of mz 301.

**Figure S2:** Product ion mass spectrum of the ion of mz 365.

**Figure S3:** Product ion mass spectrum of the ion of mz 381.

**Figure S4:** Product ion mass spectrum of the ion of mz 475.

**Figure S5:** Product ion mass spectrum of the ion of mz 493.

**Figure S6:** Product ion mass spectrum of the ion of mz 593.

**Figure S7:** Product ion mass spectrum of the ion of mz 601.

**Figure S8:** Product ion mass spectrum of the ion of mz 611.

**Figure S9:** Product ion mass spectrum of the ion of mz 703.

**Figure S10:** Product ion mass spectrum of the ion of mz 721.

**Figure S11:** Product ion mass spectrum of the ion of mz 723.

**Figure S12:** Product ion mass spectrum of the ion of mz 783.

**Figure S13:** Product ion mass spectrum of the ion of mz 825.

**Figure S14:** Product ion mass spectrum of the ion of mz 869.

**Figure S15:** Product ion mass spectrum of the ion of mz 957 cópia.

**Figure S16:** Product ion mass spectrum of the ion of mz 177 cópia.

**Figure S17:** Product ion mass spectrum of the ion of mz 179.

**Figure S18:** Product ion mass spectrum of the ion of mz 191.

**Figure S19:** Product ion mass spectrum of the ion of mz 193.

**Figure S20:** Product ion mass spectrum of the ion of mz 215.

**Figure S21:** Product ion mass spectrum of the ion of mz 255.

**Figure S22:** Product ion mass spectrum of the ion of mz 269.

**Figure S23:** Product ion mass spectrum of the ion of mz 271.

**Figure S24:** Product ion mass spectrum of the ion of mz 281.

**Figure S25:** Product ion mass spectrum of the ion of mz 283.

**Figure S26:** Product ion mass spectrum of the ion of mz 301.

**Figure S27:** Product ion mass spectrum of the ion of mz 305.

**Figure S28:** Product ion mass spectrum of the ion of mz 309.

**Figure S29:** Product ion mass spectrum of the ion of mz 311.

**Figure S30:** Product ion mass spectrum of the ion of mz 325.

**Figure S31:** Product ion mass spectrum of the ion of mz 327.

**Figure S32:** Product ion mass spectrum of the ion of mz 339.

**Figure S33:** Product ion mass spectrum of the ion of mz 341.

**Figure S34:** Product ion mass spectrum of the ion of mz 343.

**Figure S35:** Product ion mass spectrum of the ion of mz 353.

**Figure S36:** Product ion mass spectrum of the ion of mz 357.

**Figure S37:** Product ion mass spectrum of the ion of mz 359.

**Figure S38:** Product ion mass spectrum of the ion of mz 369.

**Figure S39:** Product ion mass spectrum of the ion of mz 377.

**Figure S40:** Product ion mass spectrum of the ion of mz 383.

**Figure S41:** Product ion mass spectrum of the ion of mz 387.

**Figure S42:** Product ion mass spectrum of the ion of mz 417.

**Figure S43:** Product ion mass spectrum of the ion of mz 443.

**Figure S44:** Product ion mass spectrum of the ion of mz 447.

**Figure S45:** Product ion mass spectrum of the ion of mz 463.

**Figure S46:** Product ion mass spectrum of the ion of mz 483.

**Figure S47:** Product ion mass spectrum of the ion of mz 505.

**Figure S48:** Product ion mass spectrum of the ion of mz 521.

**Figure S49:** Product ion mass spectrum of the ion of mz 535.

**Figure S50:** Product ion mass spectrum of the ion of mz 615.

**Figure S51:** Product ion mass spectrum of the ion of mz 631.

**Figure S52:** Product ion mass spectrum of the ion of mz 633.

**Figure S53:** Product ion mass spectrum of the ion of mz 635.

**Figure S54:** Product ion mass spectrum of the ion of mz 643.

**Figure S55:** Product ion mass spectrum of the ion of mz 683.

**Figure S56:** Product ion mass spectrum of the ion of mz 761.

**Figure S57:** Product ion mass spectrum of the ion of mz 783.

**Figure S58:** Product ion mass spectrum of the ion of mz 785.

**Figure S59:** Product ion mass spectrum of the ion of mz 865.

**Figure S60:** Product ion mass spectrum of the ion of mz 933.

**Figure S61:** Product ion mass spectrum of the ion of mz 951.

## Data Availability

The data that supports the findings of this study are available in the Supporting Information of this article.
