# Supplementary figures and images for: Evaluation of the Chemical Profile by Paper Spray Mass Spectrometry of Eugenia uniflora Pulp, Peel, Seeds, and Jelly
Source: J Mass Spectrom. 2025 Sep 16;60(10):e5173. doi: 10.1002/jms.5173 (PMC12439022; doi:10.1002/jms.5173)

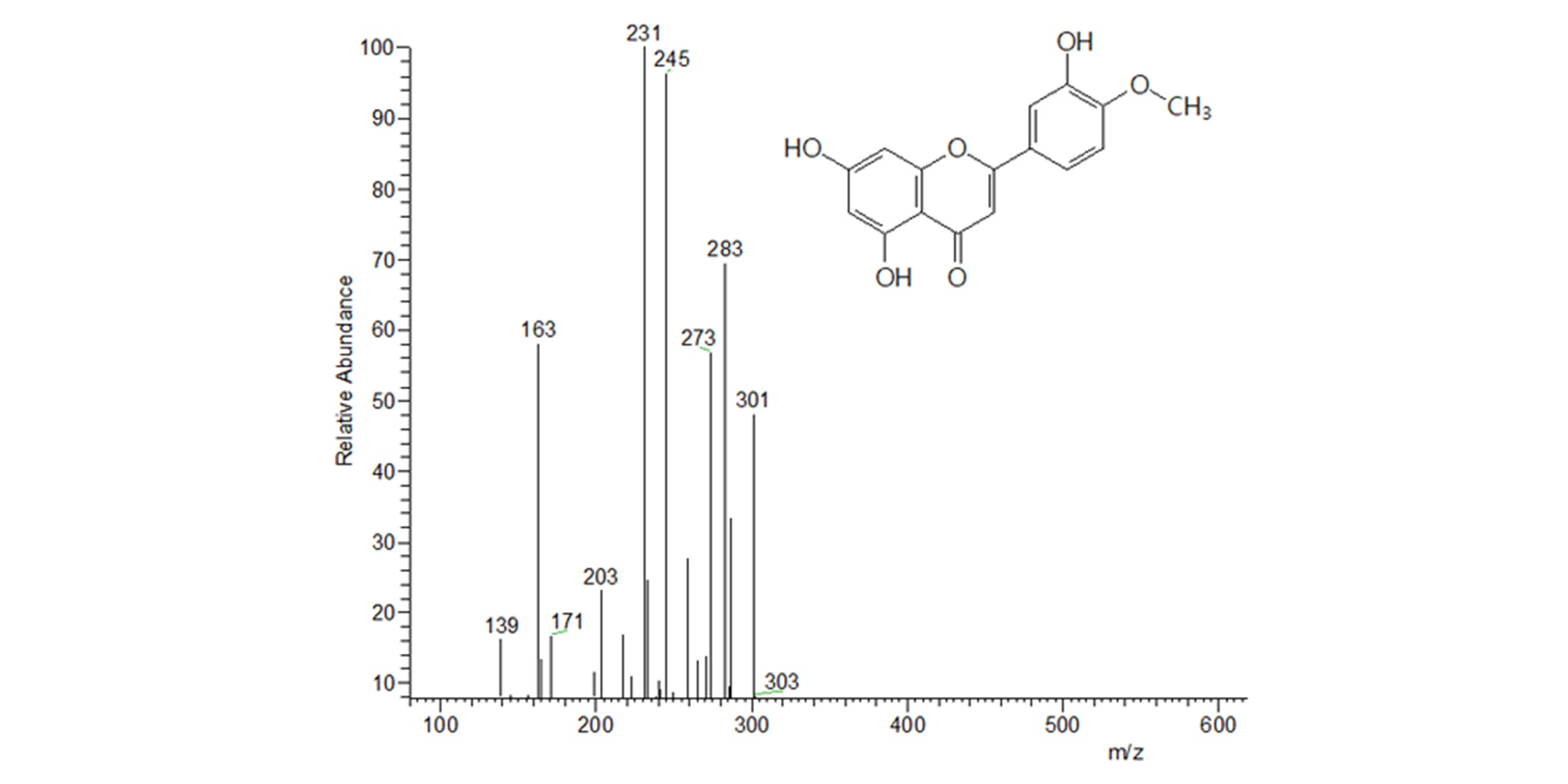

Supplement: Supplementary file 1 — Figure S1: Product ion mass spectrum of the ion of mz 301. [file JMS-60-e5173-s008.jpg]

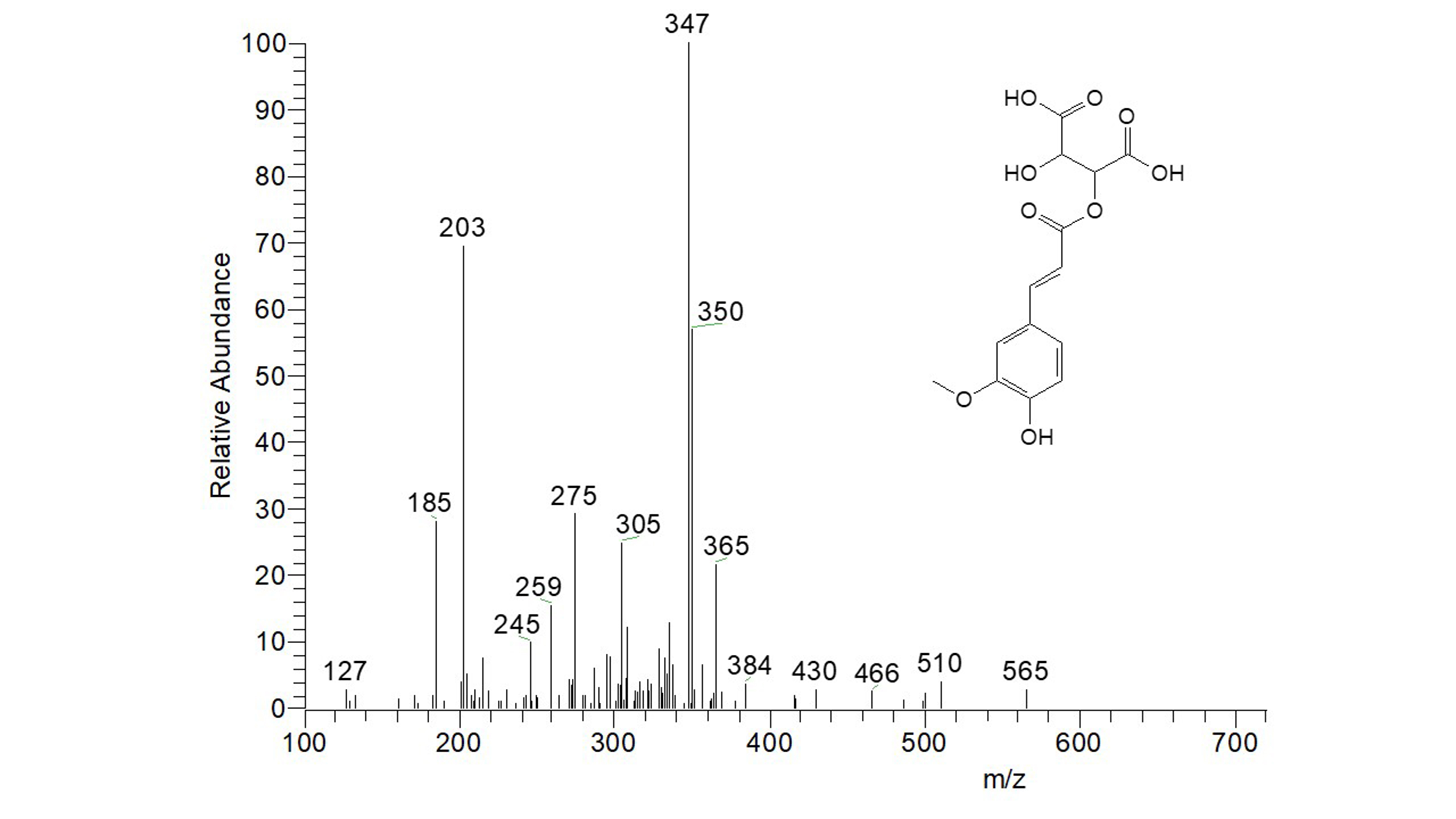

Supplement: Supplementary file 2 — Figure S2: Product ion mass spectrum of the ion of mz 365. [file JMS-60-e5173-s031.jpg]

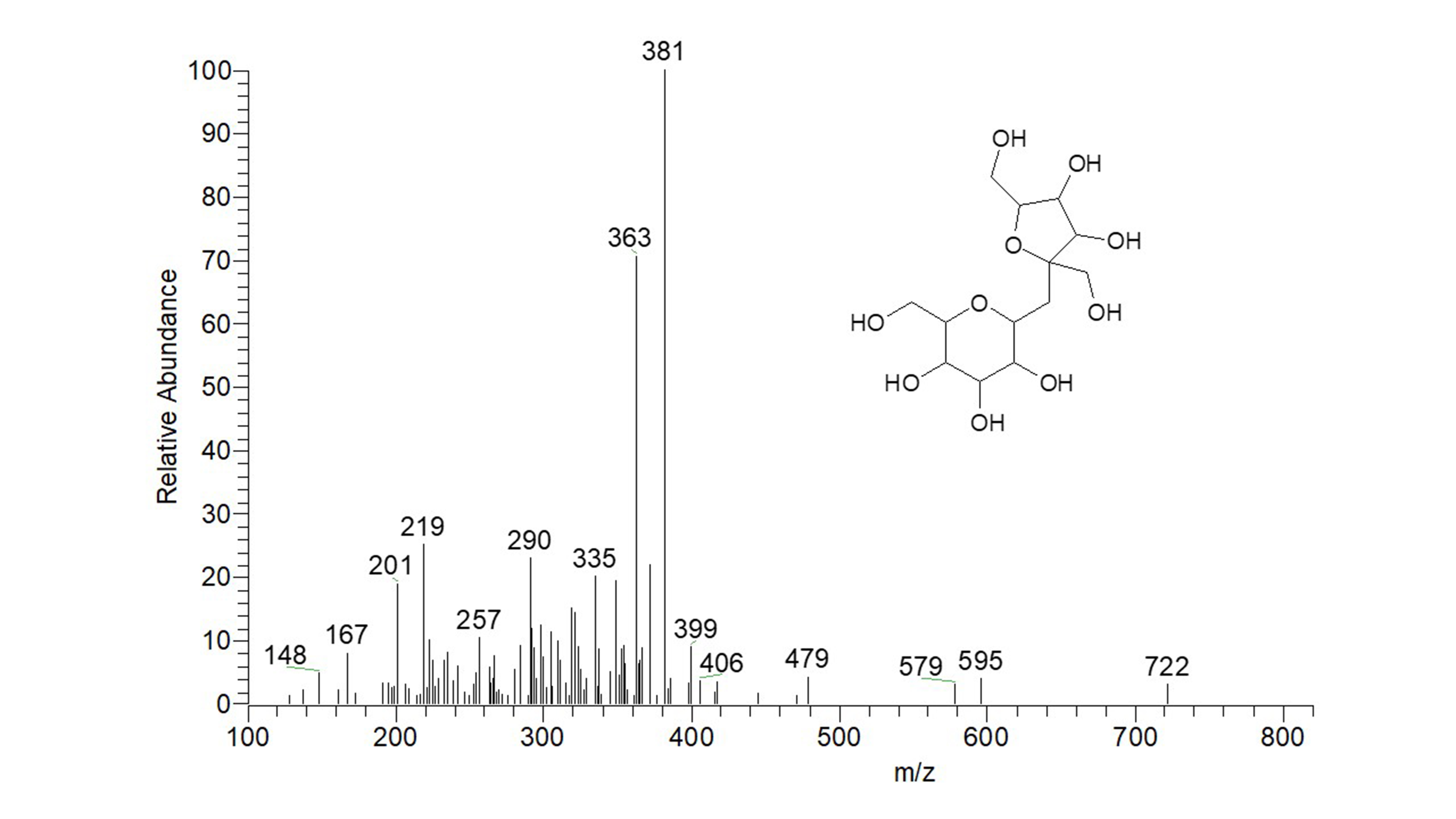

Supplement: Supplementary file 3 — Figure S3: Product ion mass spectrum of the ion of mz 381. [file JMS-60-e5173-s061.jpg]

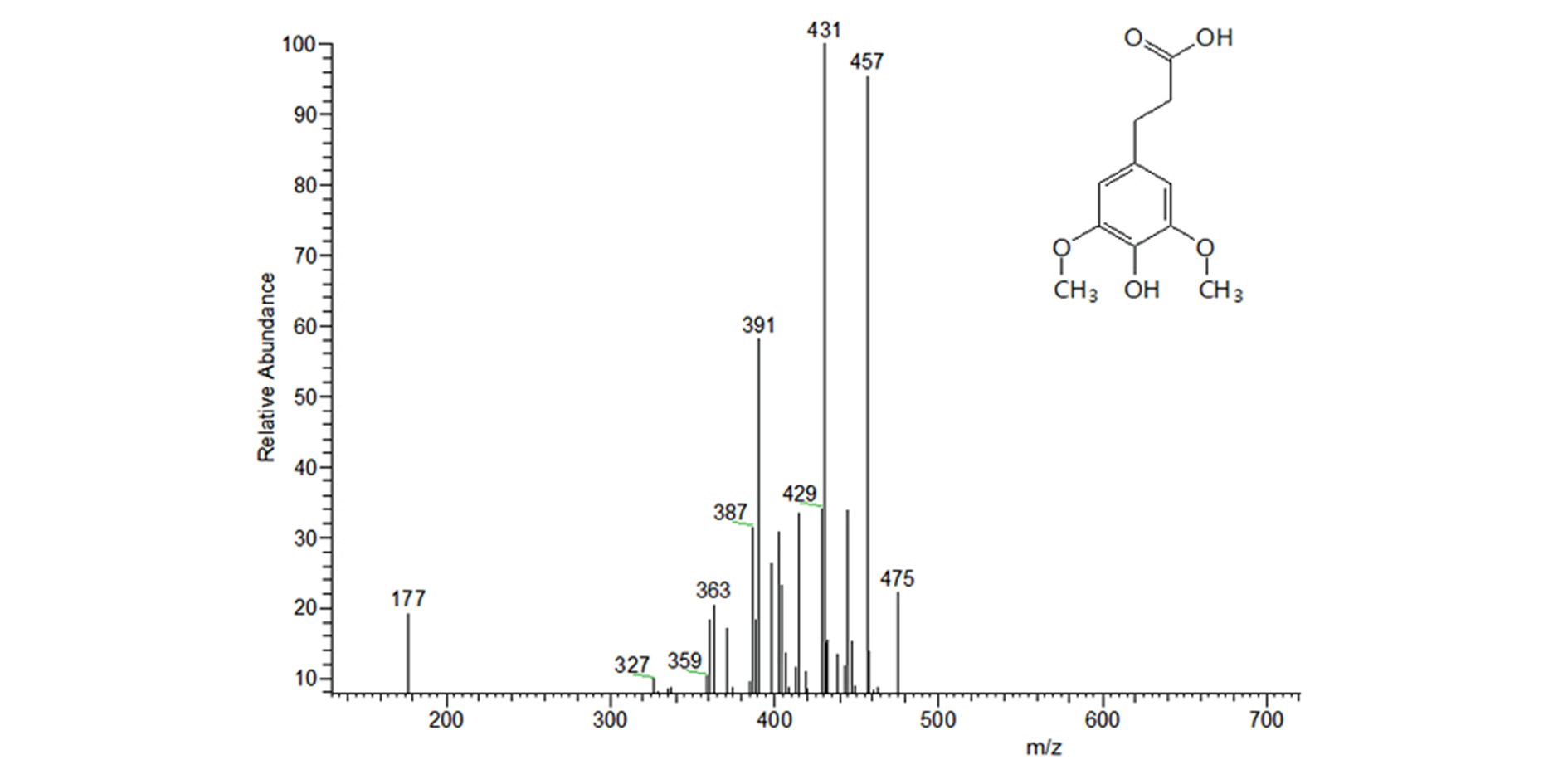

Supplement: Supplementary file 4 — Figure S4: Product ion mass spectrum of the ion of mz 475. [file JMS-60-e5173-s049.jpg]

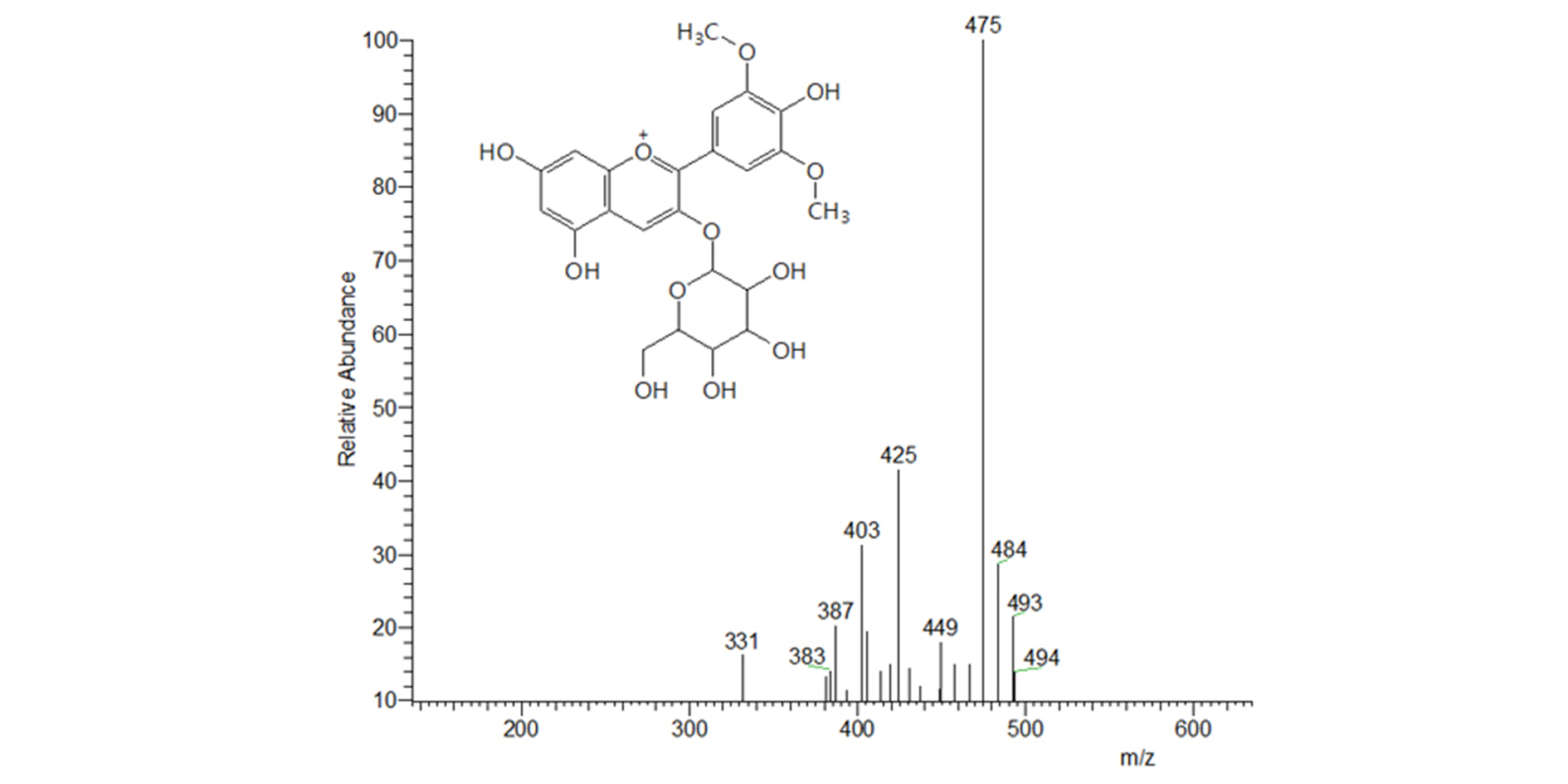

Supplement: Supplementary file 5 — Figure S5: Product ion mass spectrum of the ion of mz 493. [file JMS-60-e5173-s017.jpg]

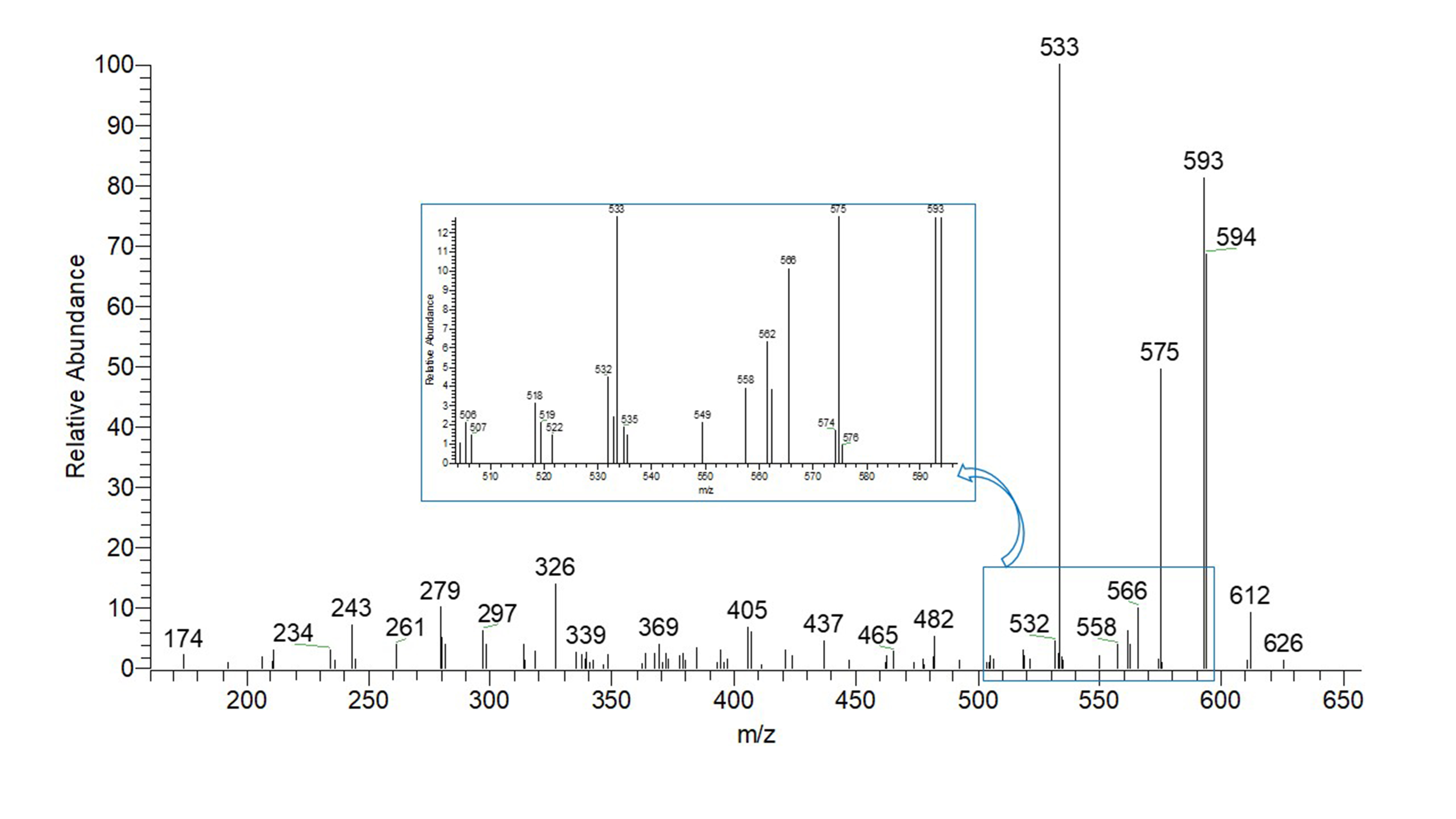

Supplement: Supplementary file 6 — Figure S6: Product ion mass spectrum of the ion of mz 593. [file JMS-60-e5173-s001.jpg]

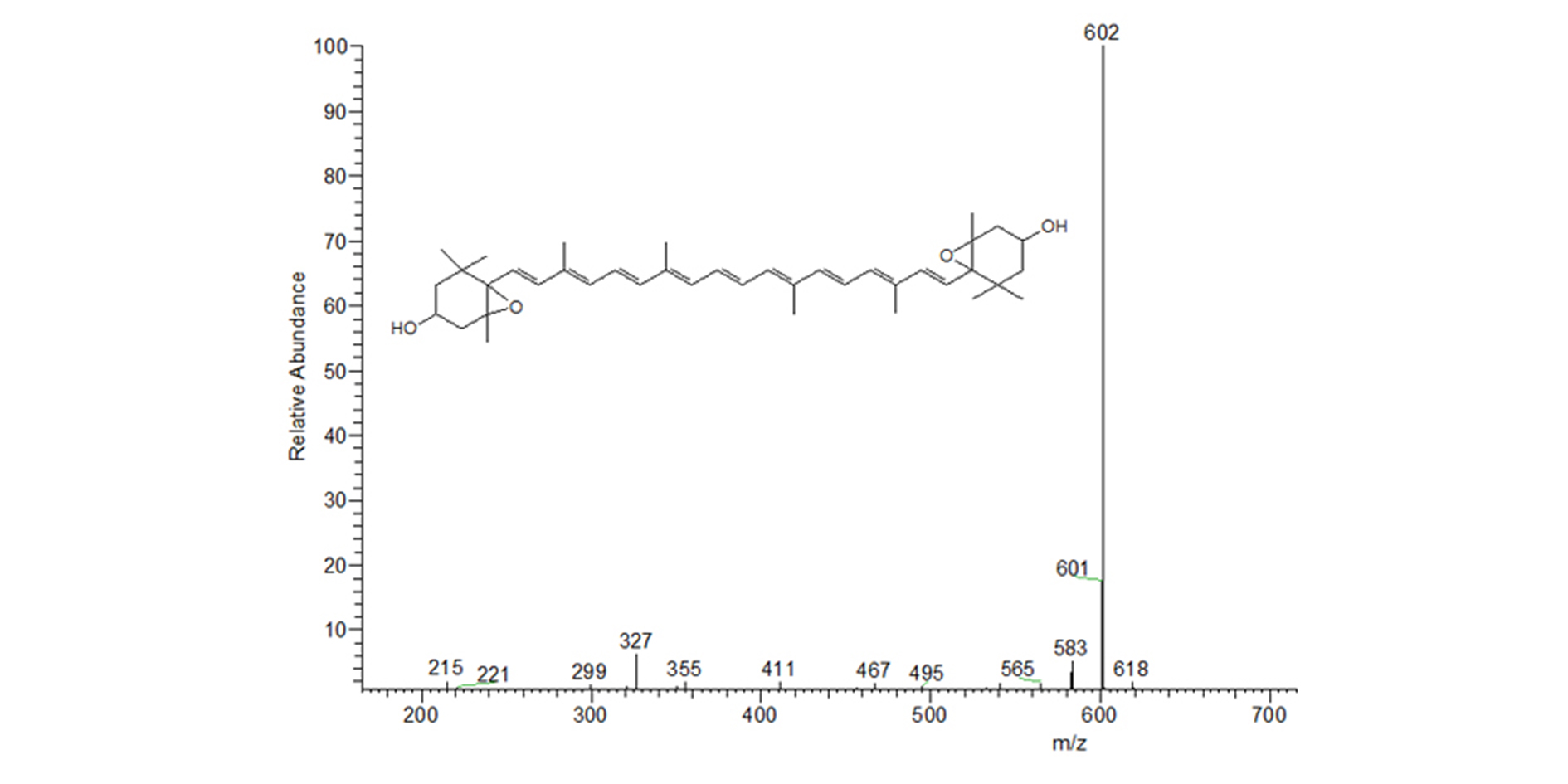

Supplement: Supplementary file 7 — Figure S7: Product ion mass spectrum of the ion of mz 601. [file JMS-60-e5173-s032.jpg]

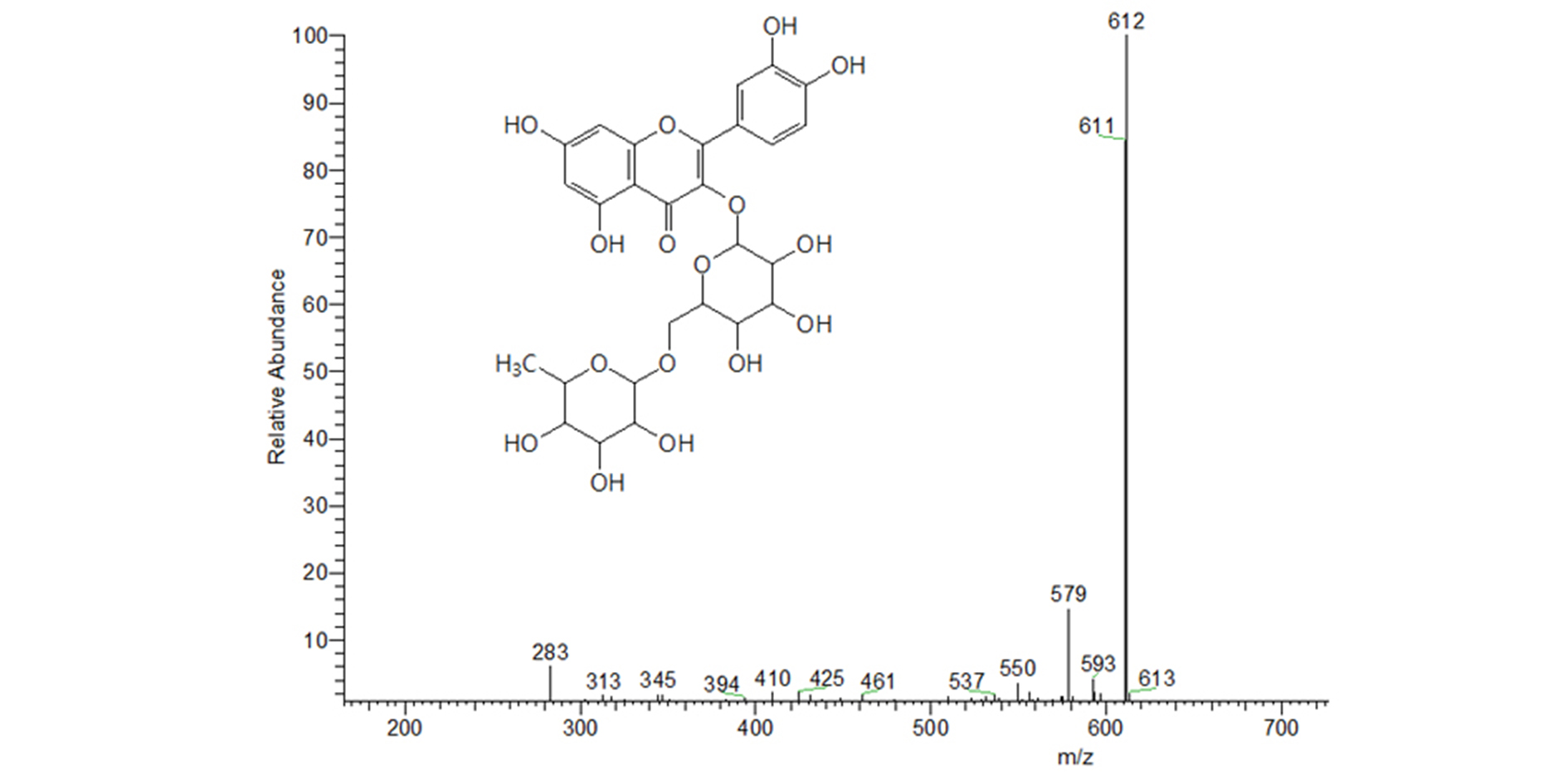

Supplement: Supplementary file 8 — Figure S8: Product ion mass spectrum of the ion of mz 611. [file JMS-60-e5173-s022.jpg]

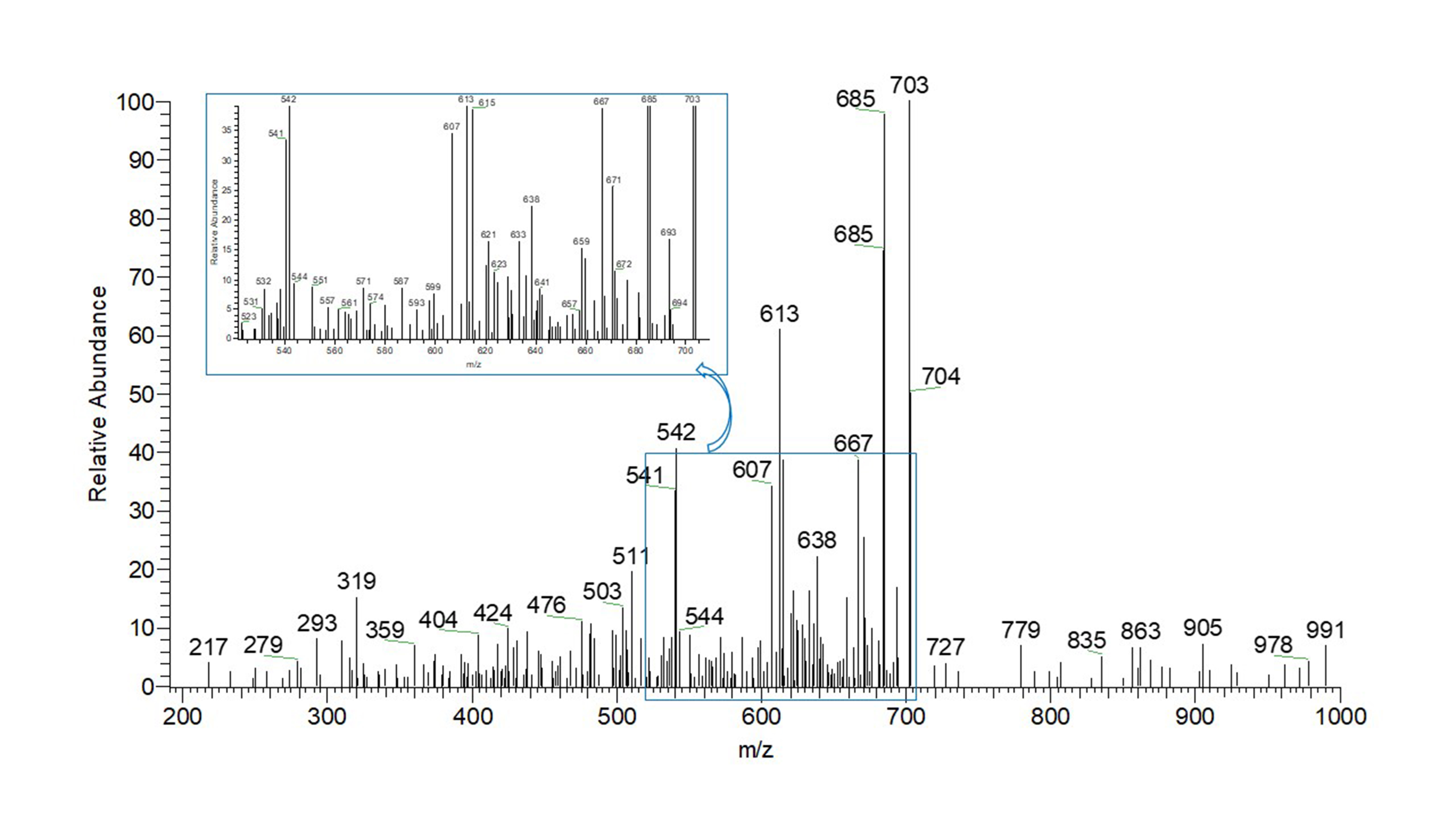

Supplement: Supplementary file 9 — Figure S9: Product ion mass spectrum of the ion of mz 703. [file JMS-60-e5173-s052.jpg]

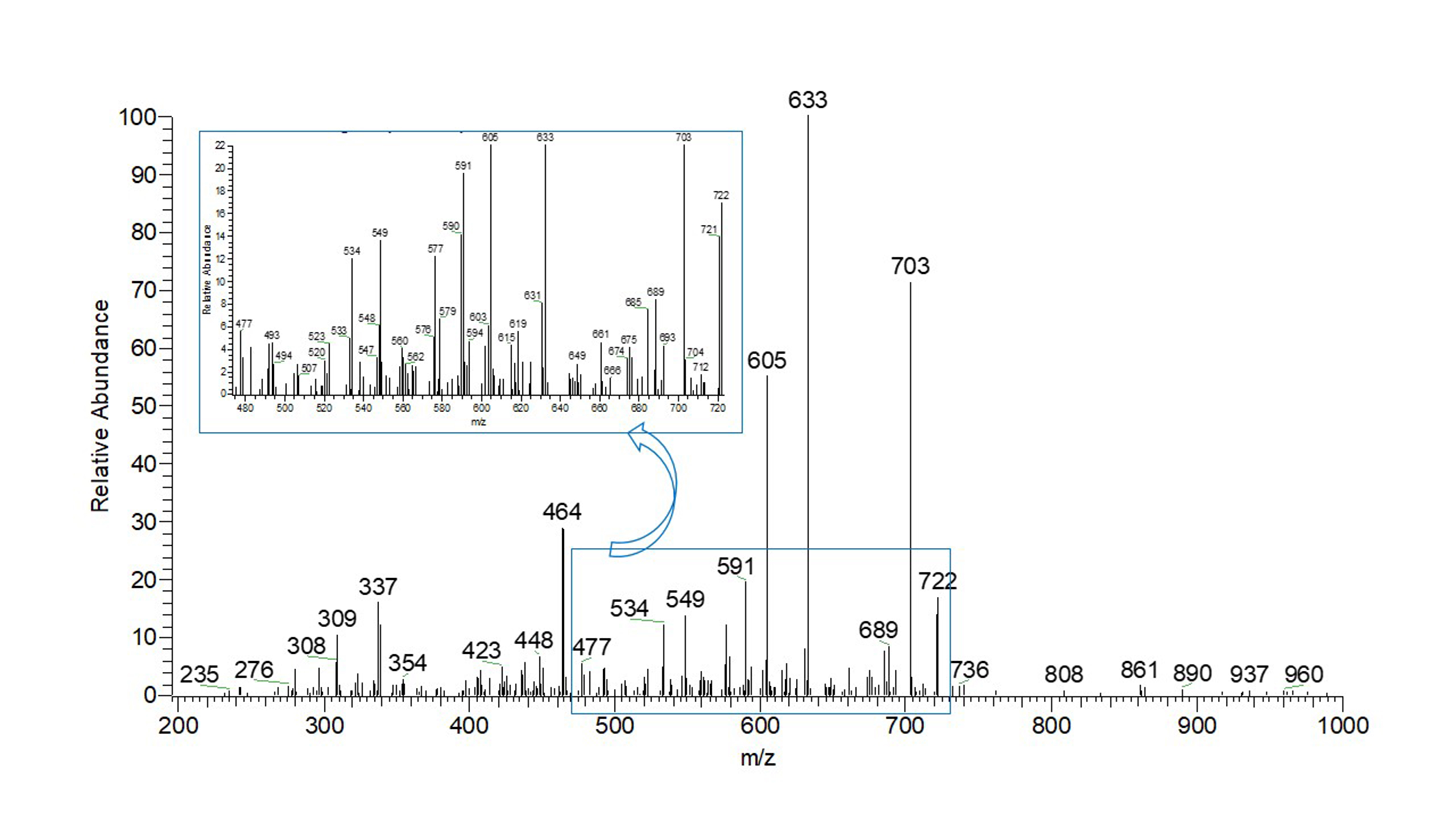

Supplement: Supplementary file 10 — Figure S10: Product ion mass spectrum of the ion of mz 721. [file JMS-60-e5173-s055.jpg]

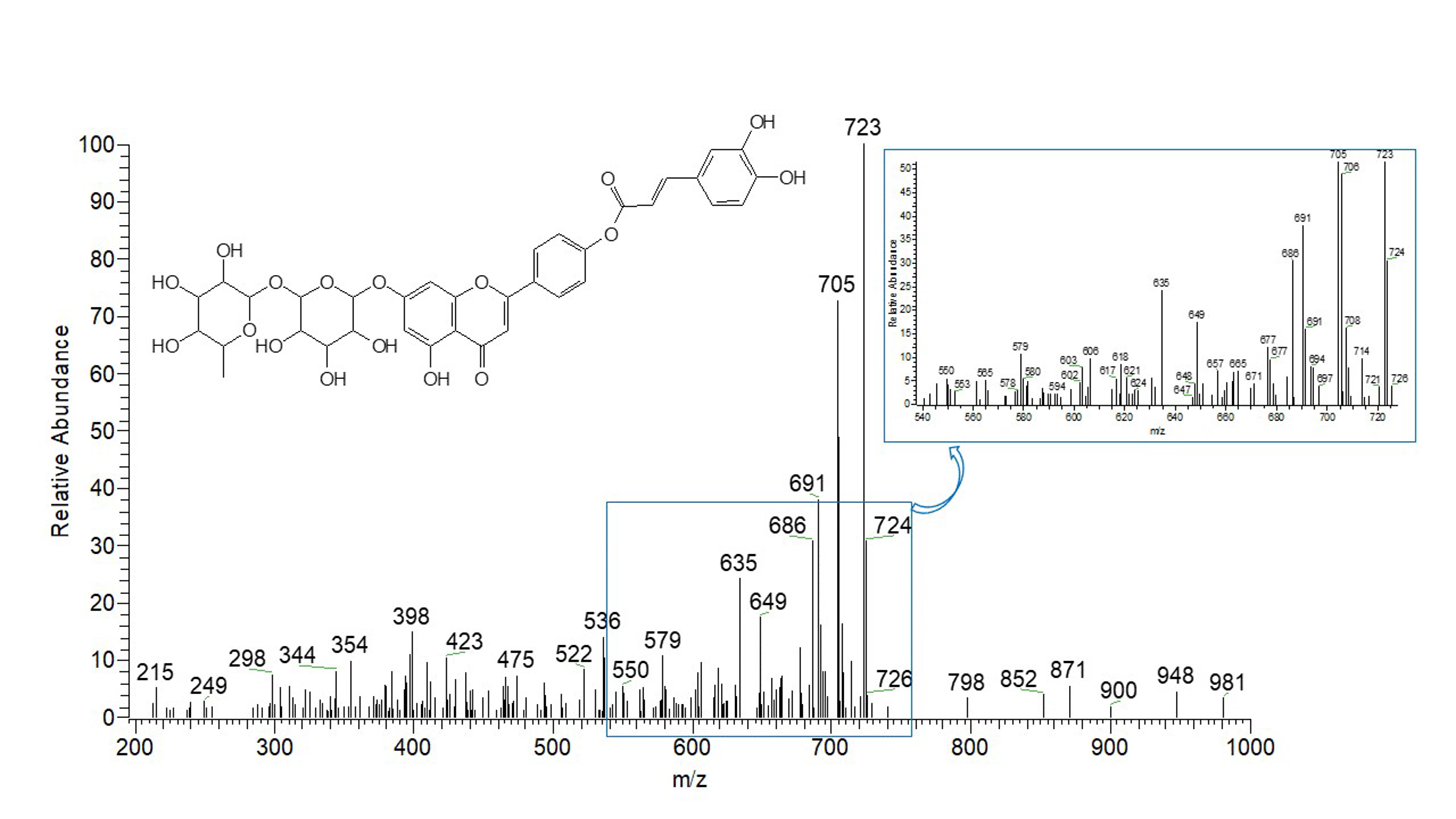

Supplement: Supplementary file 11 — Figure S11: Product ion mass spectrum of the ion of mz 723. [file JMS-60-e5173-s027.jpg]

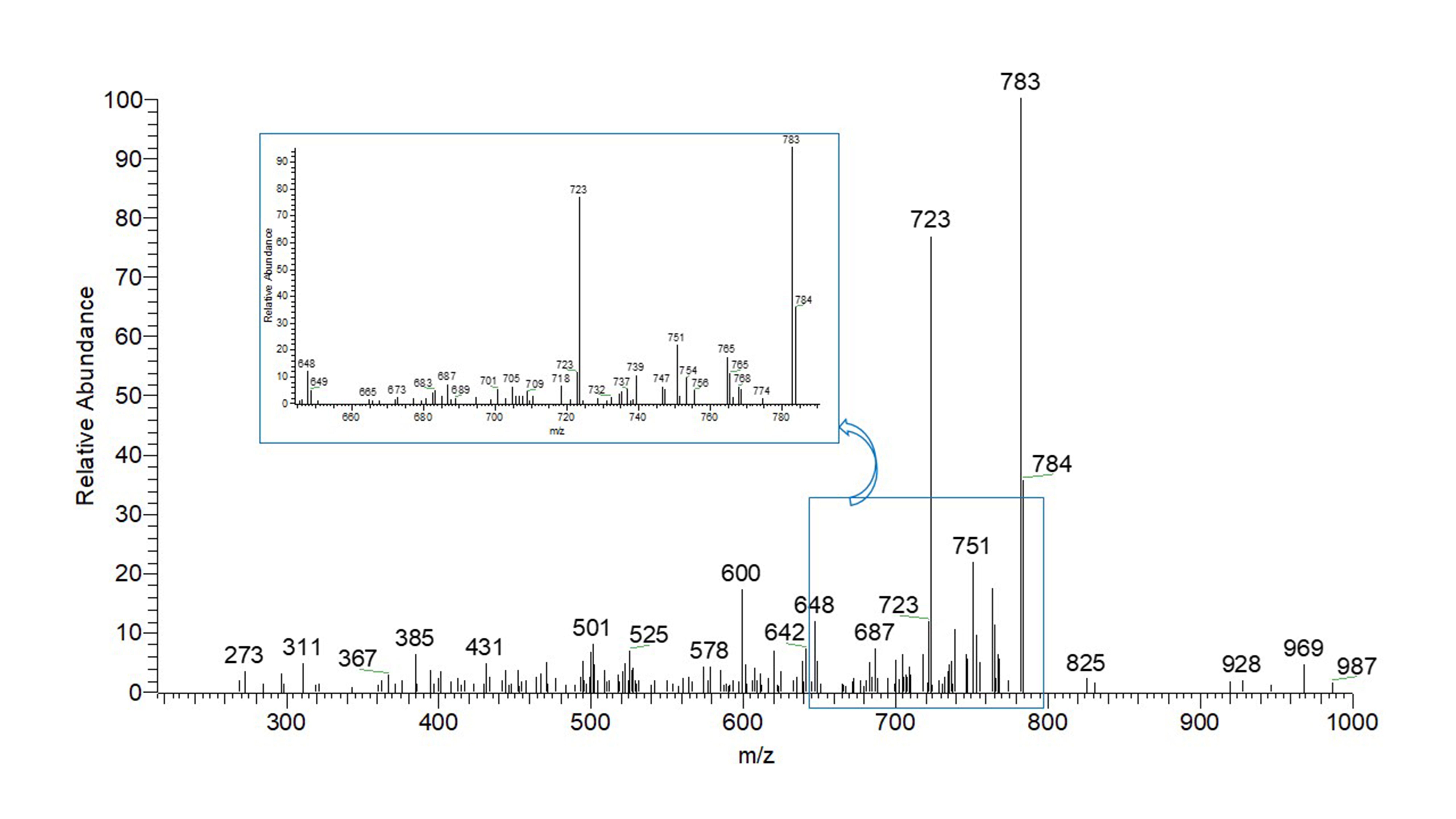

Supplement: Supplementary file 12 — Figure S12: Product ion mass spectrum of the ion of mz 783. [file JMS-60-e5173-s007.jpg]

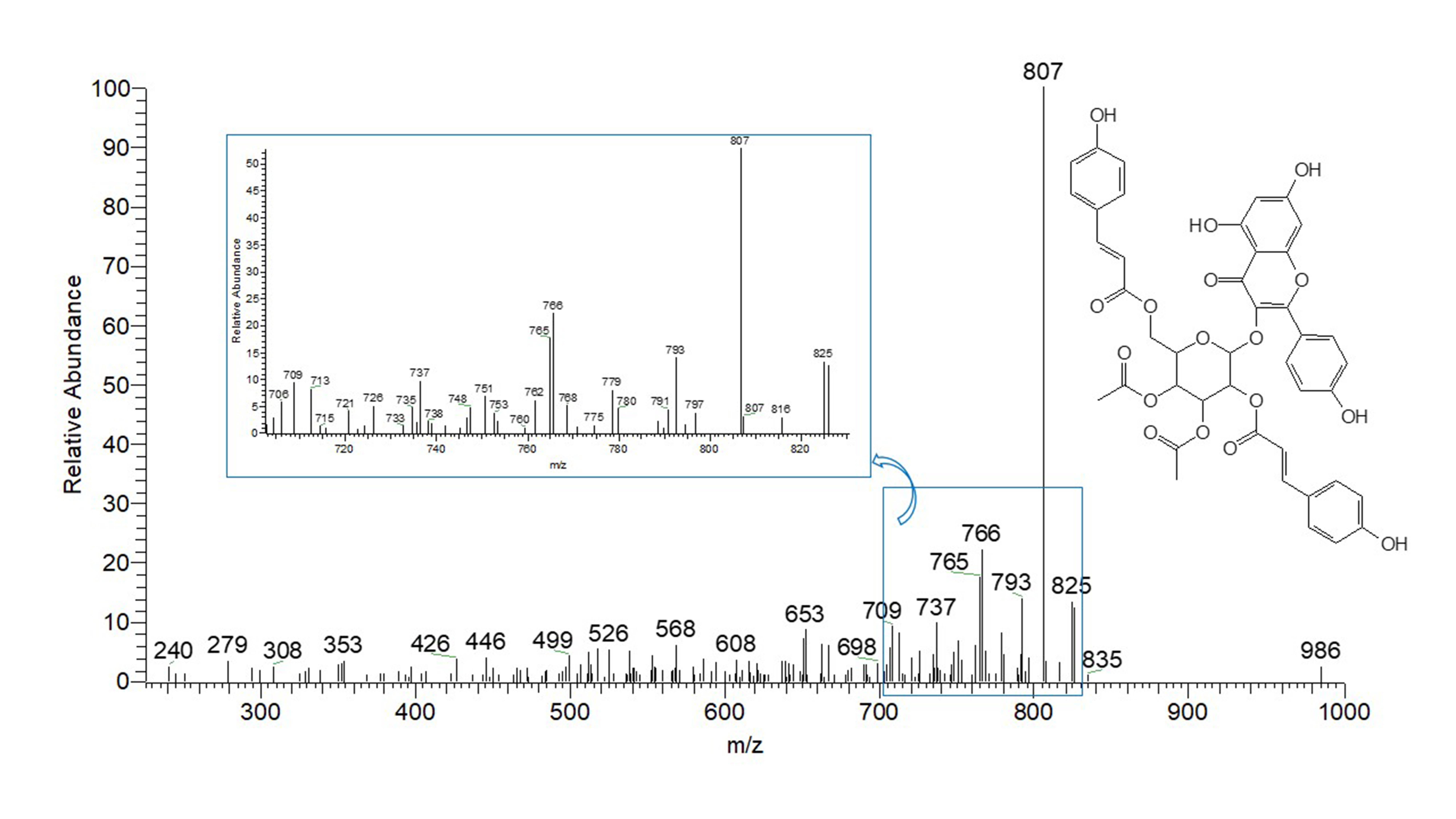

Supplement: Supplementary file 13 — Figure S13: Product ion mass spectrum of the ion of mz 825. [file JMS-60-e5173-s035.jpg]

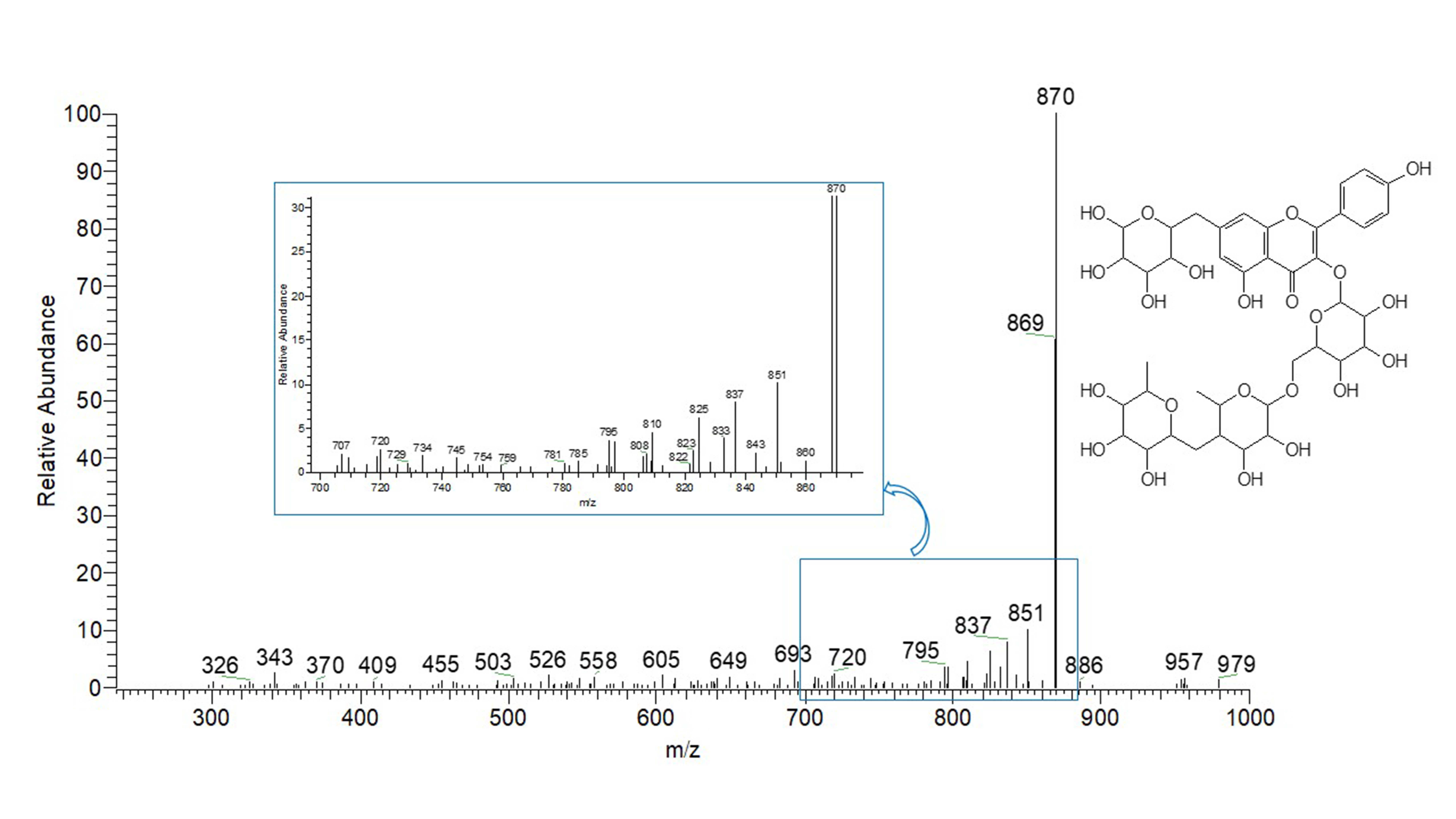

Supplement: Supplementary file 14 — Figure S14: Product ion mass spectrum of the ion of mz 869. [file JMS-60-e5173-s046.jpg]

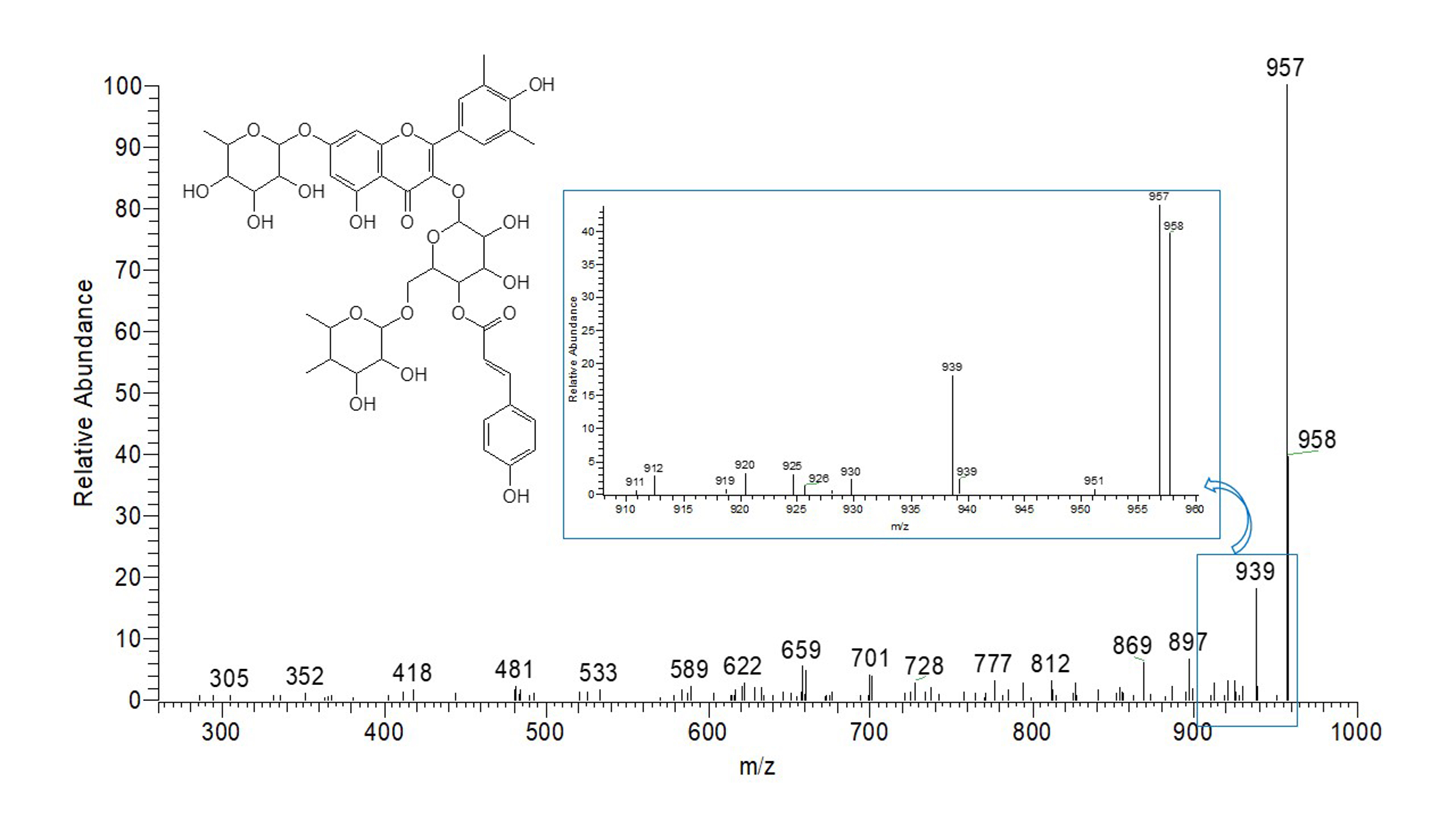

Supplement: Supplementary file 15 — Figure S15: Product ion mass spectrum of the ion of mz 957 cópia. [file JMS-60-e5173-s021.jpg]

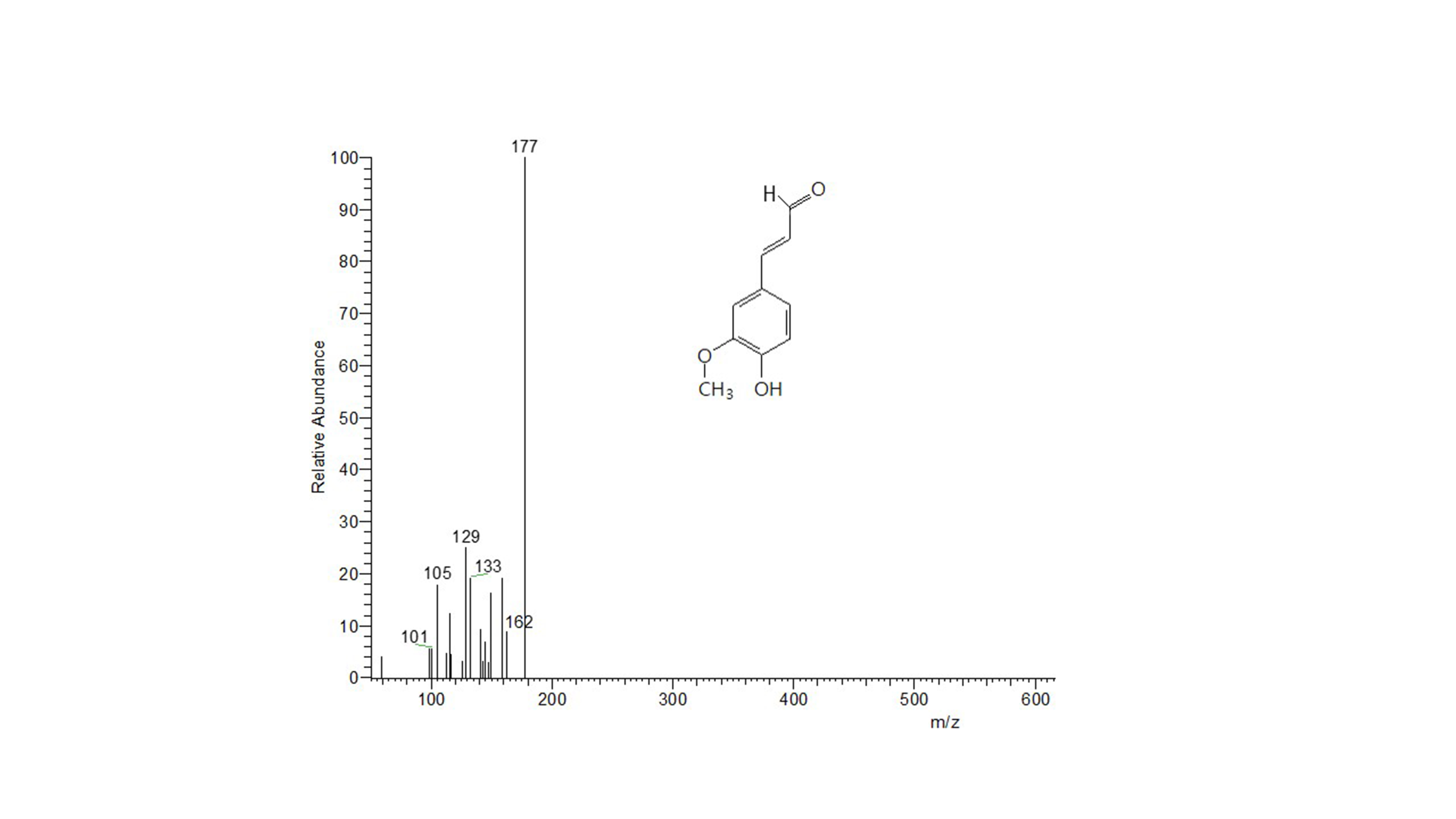

Supplement: Supplementary file 16 — Figure S16: Product ion mass spectrum of the ion of mz 177 cópia. [file JMS-60-e5173-s015.jpg]

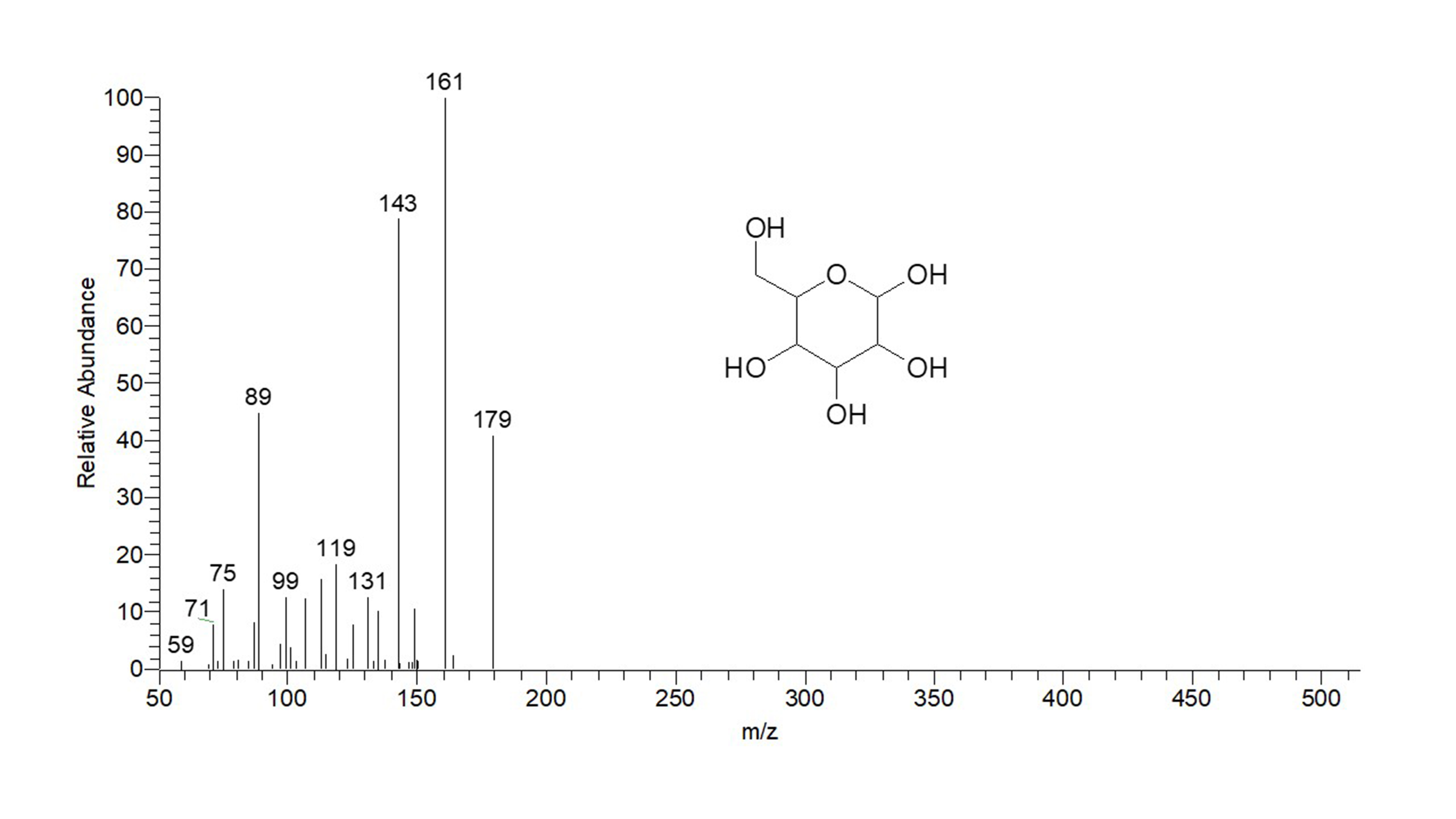

Supplement: Supplementary file 17 — Figure S17: Product ion mass spectrum of the ion of mz 179. [file JMS-60-e5173-s043.jpg]

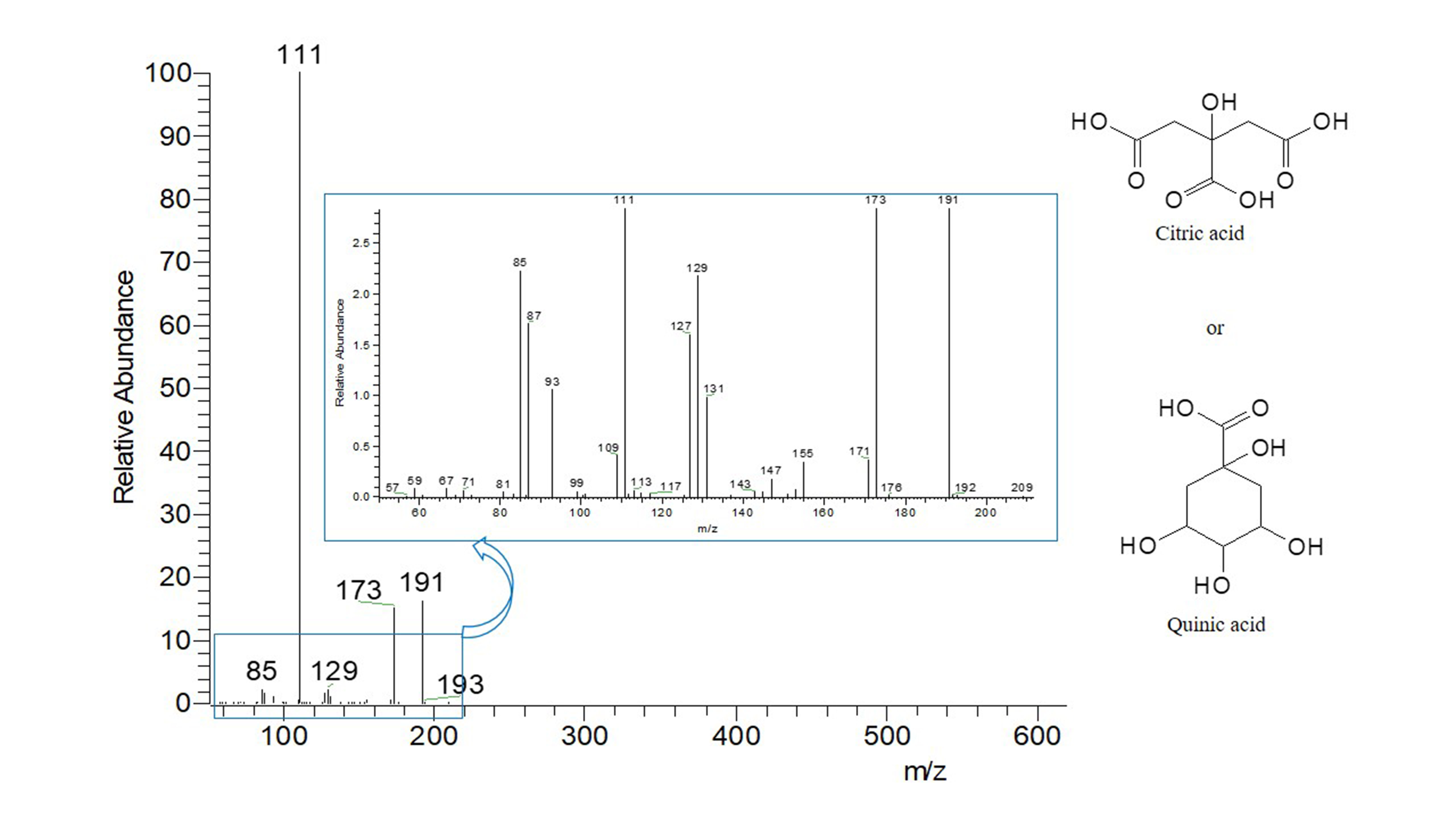

Supplement: Supplementary file 18 — Figure S18: Product ion mass spectrum of the ion of mz 191. [file JMS-60-e5173-s039.jpg]

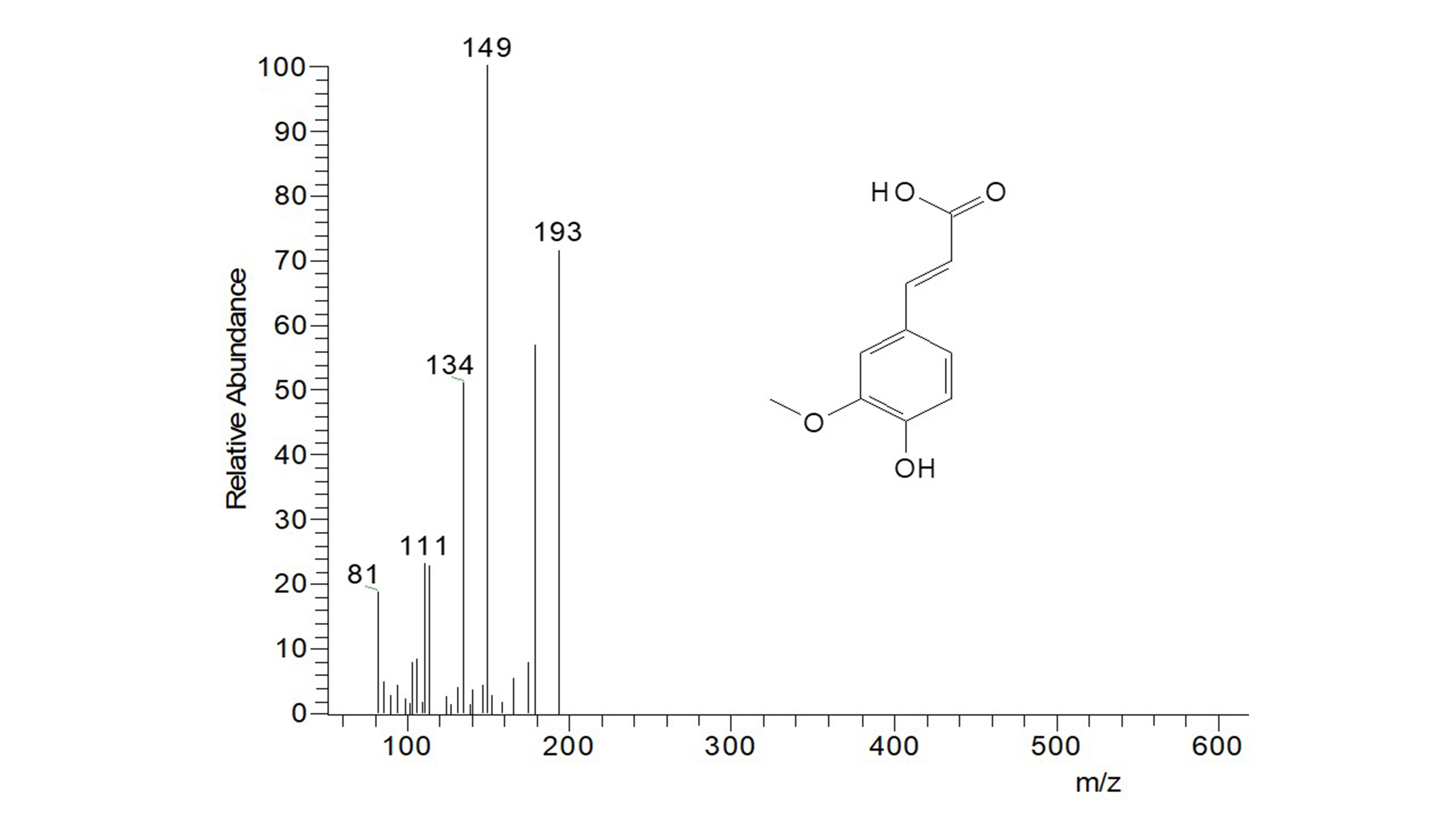

Supplement: Supplementary file 19 — Figure S19: Product ion mass spectrum of the ion of mz 193. [file JMS-60-e5173-s011.jpg]

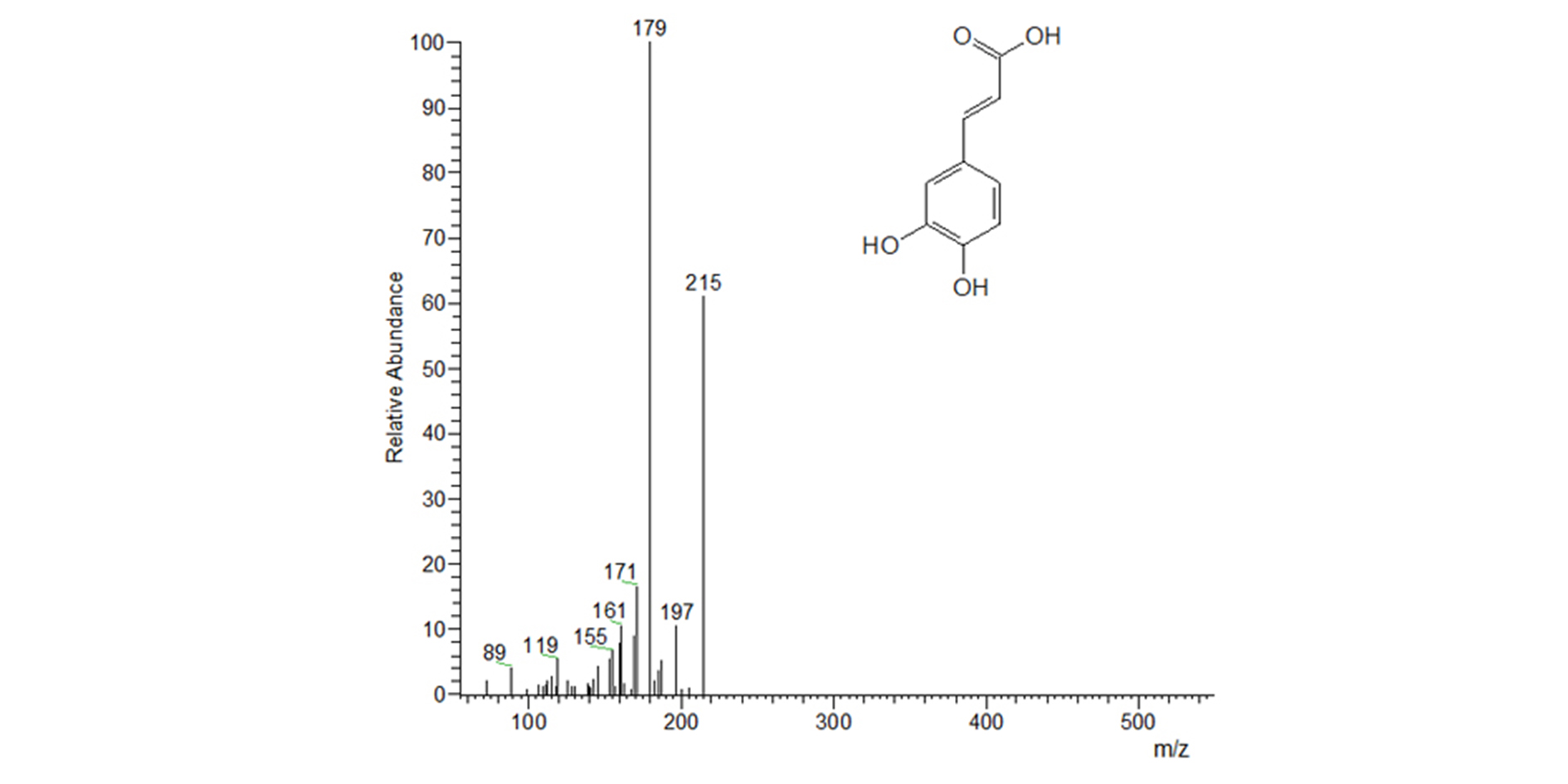

Supplement: Supplementary file 20 — Figure S20: Product ion mass spectrum of the ion of mz 215. [file JMS-60-e5173-s012.jpg]

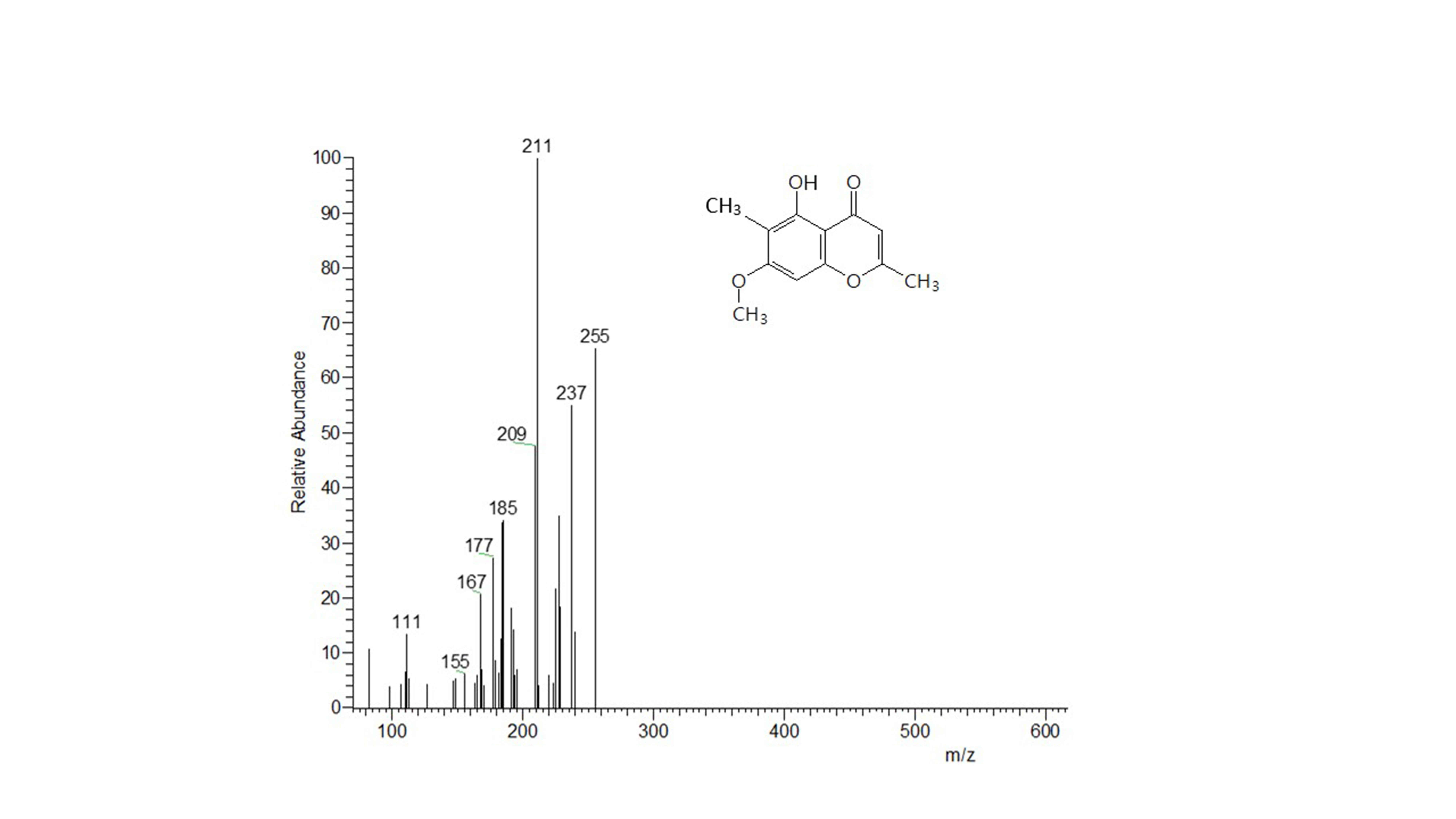

Supplement: Supplementary file 21 — Figure S21: Product ion mass spectrum of the ion of mz 255. [file JMS-60-e5173-s041.jpg]

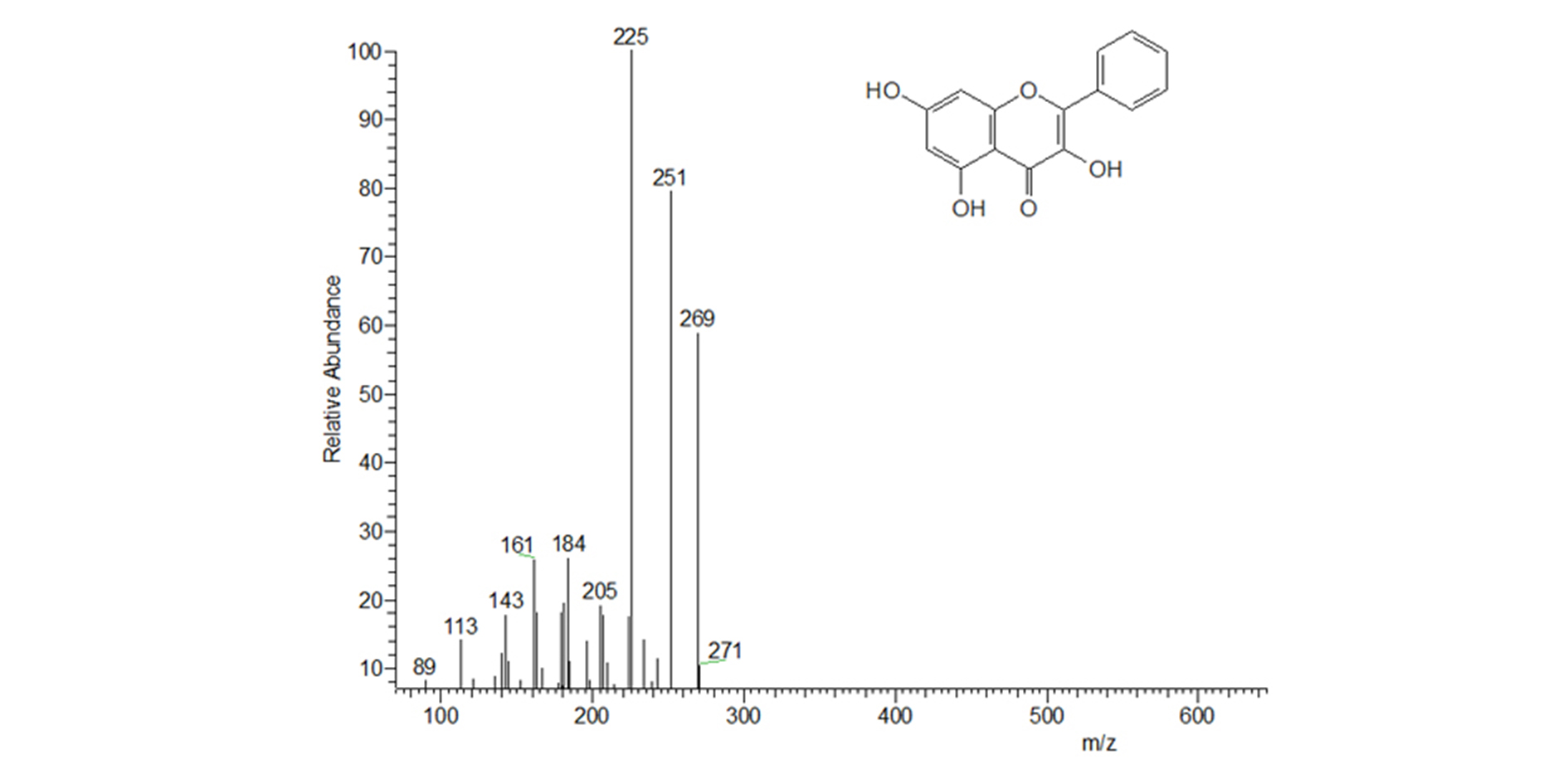

Supplement: Supplementary file 22 — Figure S22: Product ion mass spectrum of the ion of mz 269. [file JMS-60-e5173-s047.jpg]

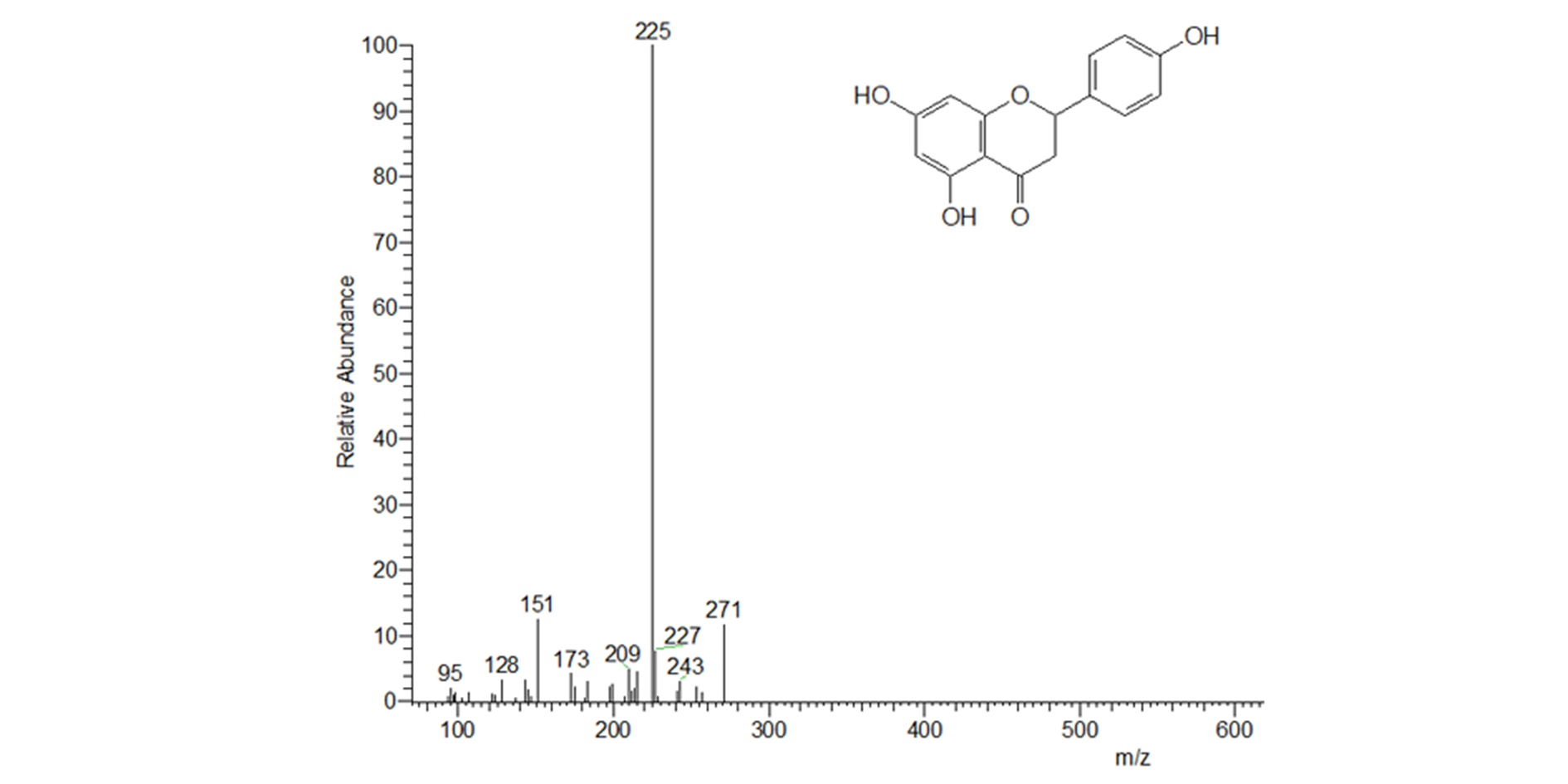

Supplement: Supplementary file 23 — Figure S23: Product ion mass spectrum of the ion of mz 271. [file JMS-60-e5173-s024.jpg]

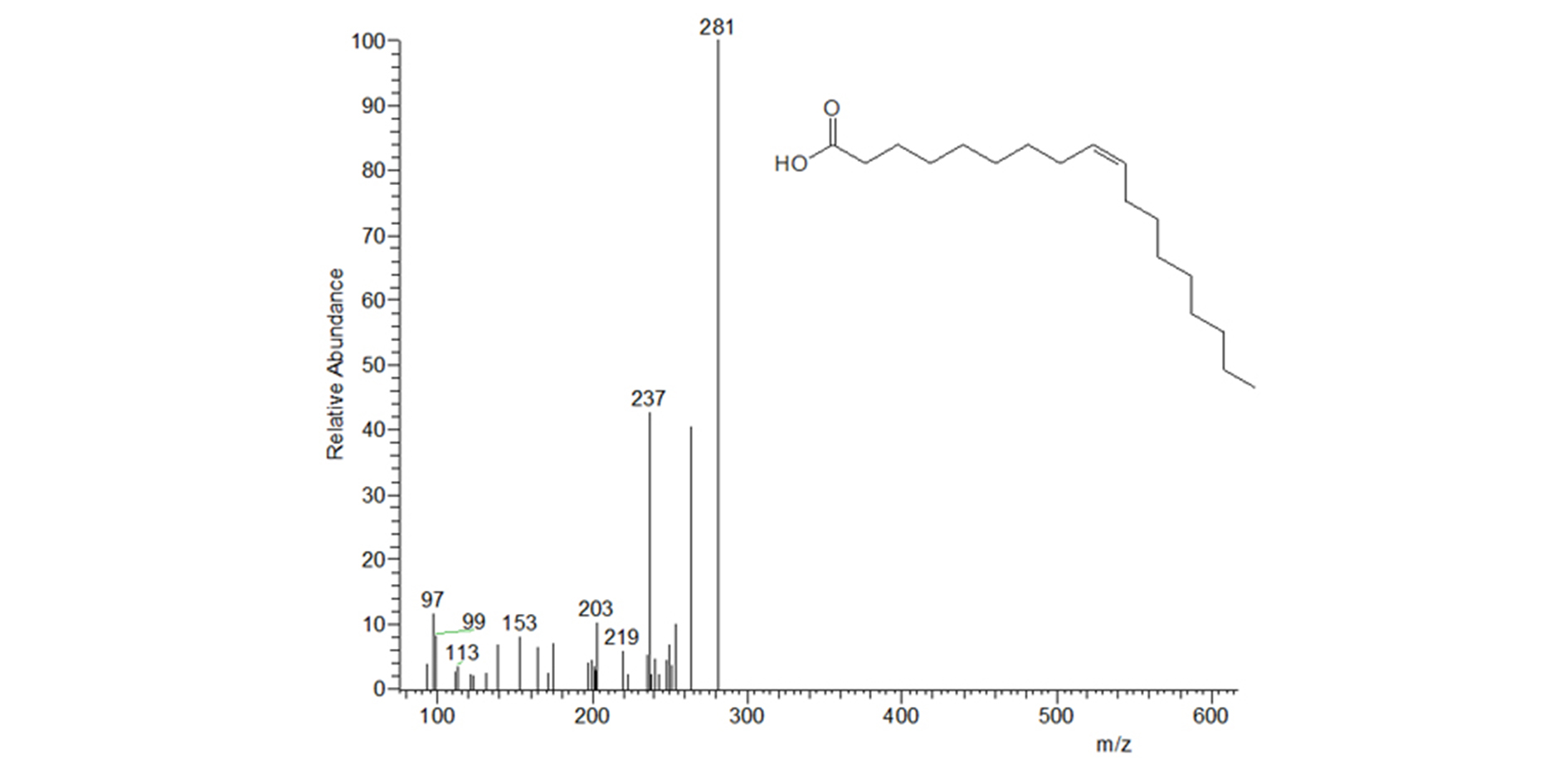

Supplement: Supplementary file 24 — Figure S24: Product ion mass spectrum of the ion of mz 281. [file JMS-60-e5173-s005.jpg]

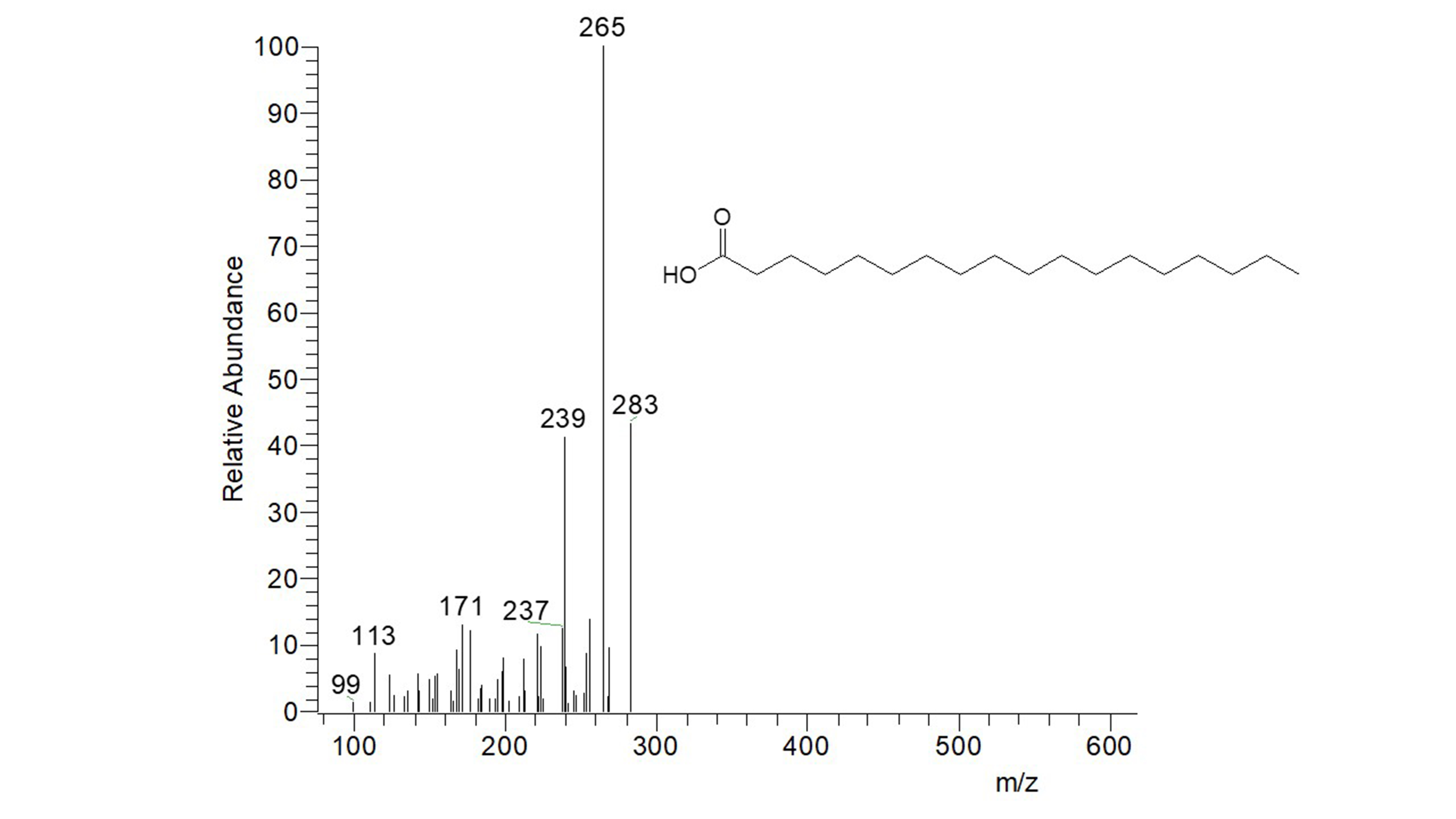

Supplement: Supplementary file 25 — Figure S25: Product ion mass spectrum of the ion of mz 283. [file JMS-60-e5173-s034.jpg]

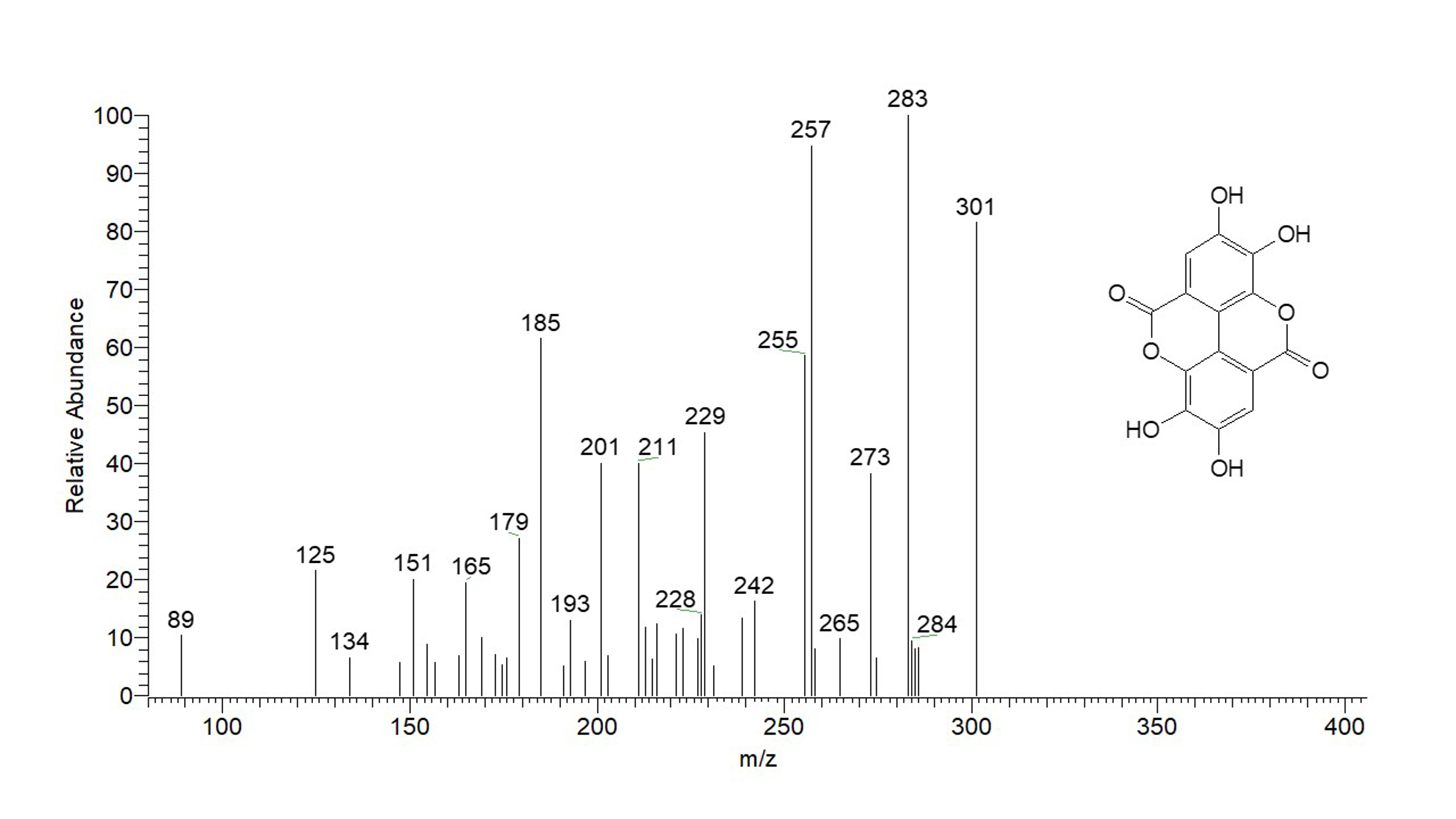

Supplement: Supplementary file 26 — Figure S26: Product ion mass spectrum of the ion of mz 301. [file JMS-60-e5173-s057.jpg]

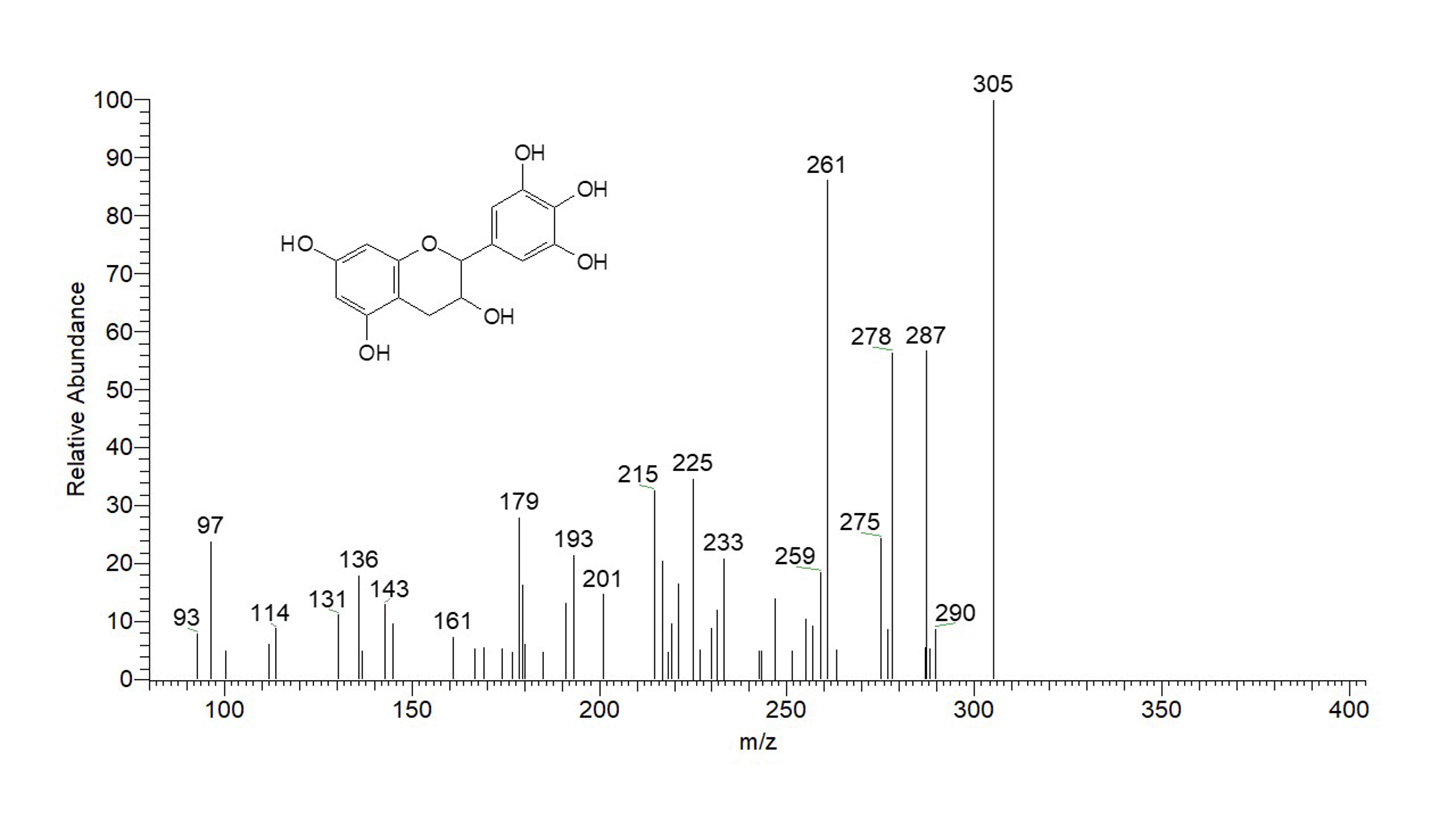

Supplement: Supplementary file 27 — Figure S27: Product ion mass spectrum of the ion of mz 305. [file JMS-60-e5173-s030.jpg]

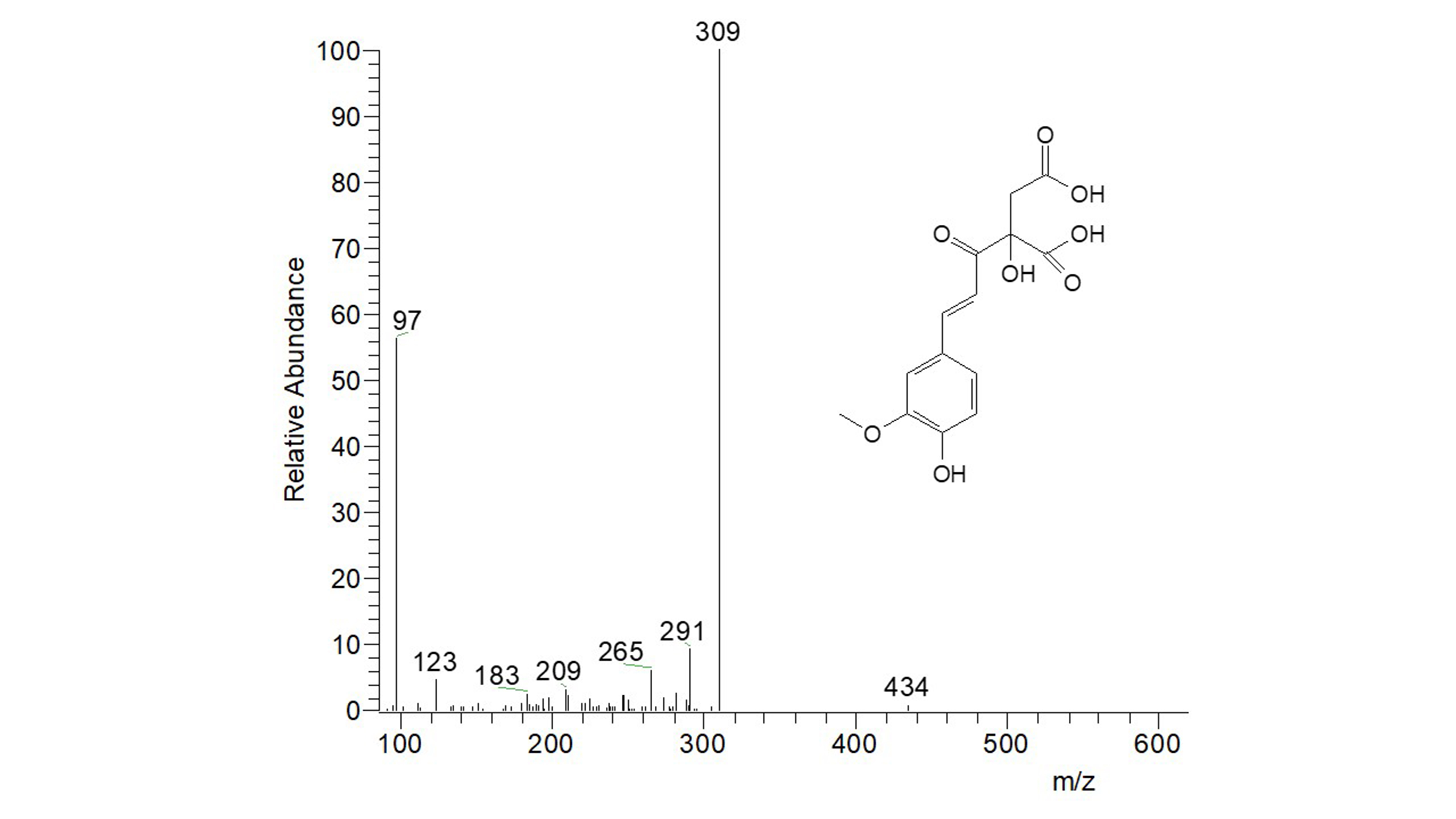

Supplement: Supplementary file 28 — Figure S28: Product ion mass spectrum of the ion of mz 309. [file JMS-60-e5173-s028.jpg]

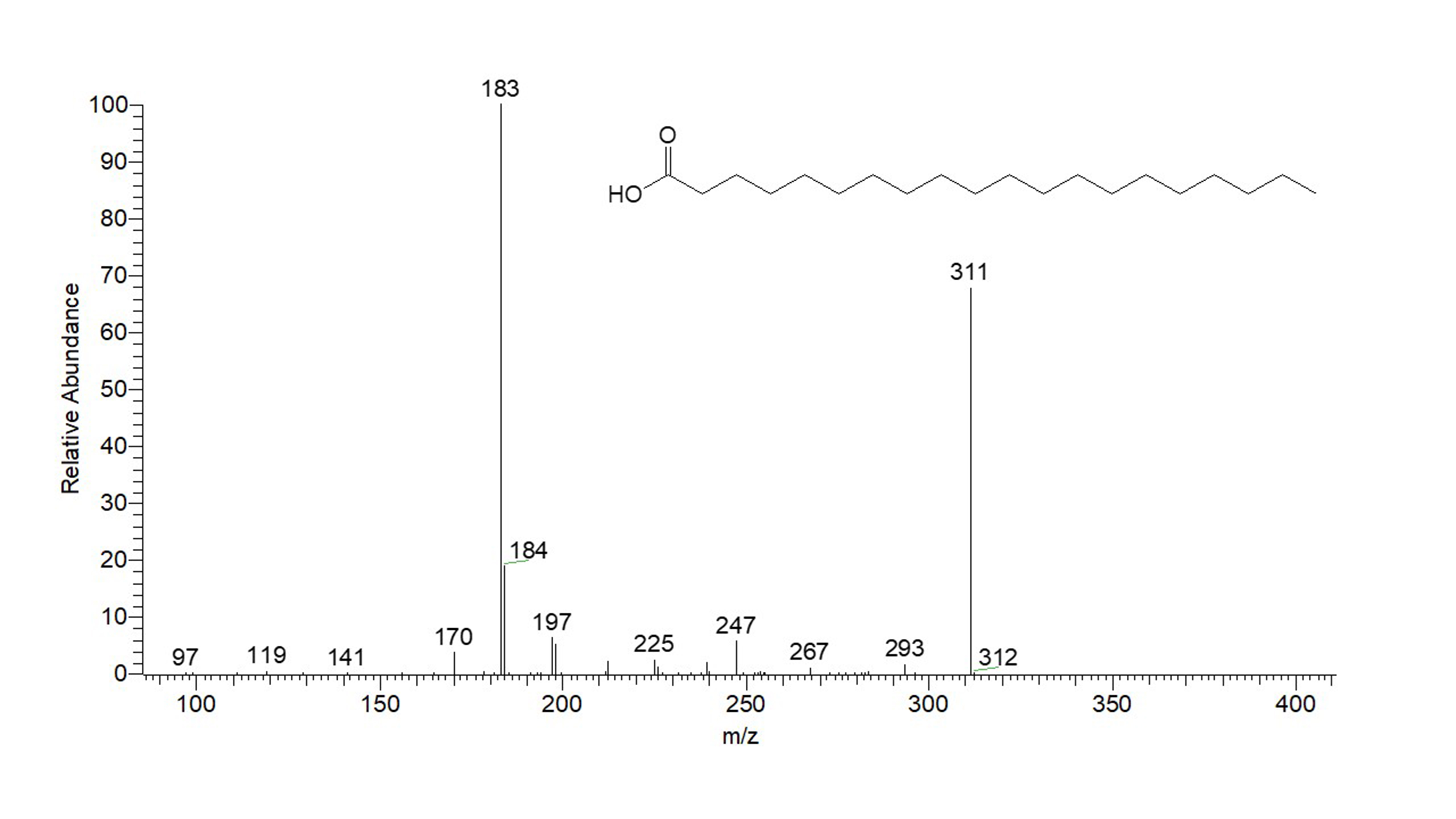

Supplement: Supplementary file 29 — Figure S29: Product ion mass spectrum of the ion of mz 311. [file JMS-60-e5173-s056.jpg]

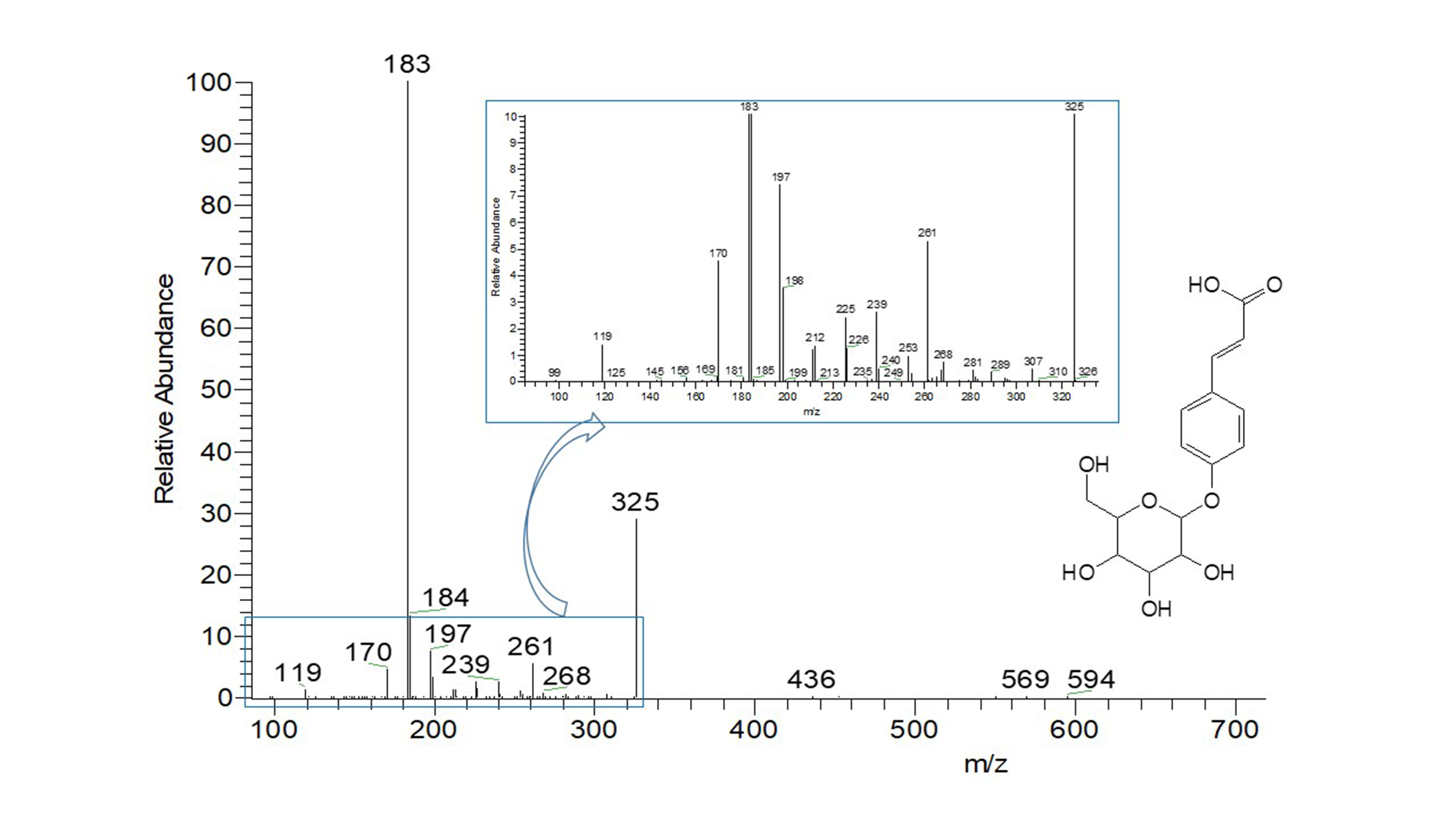

Supplement: Supplementary file 30 — Figure S30: Product ion mass spectrum of the ion of mz 325. [file JMS-60-e5173-s029.jpg]

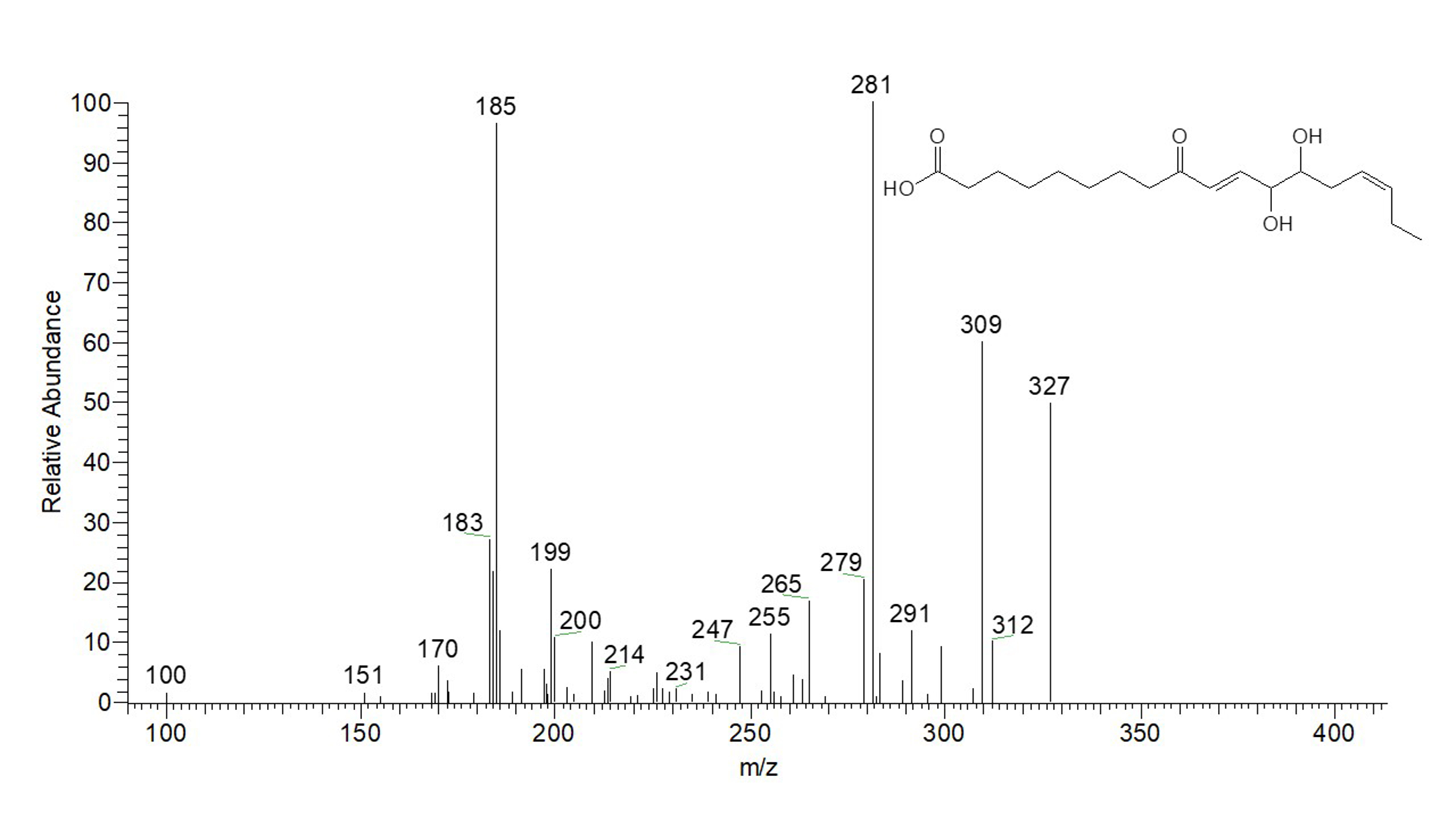

Supplement: Supplementary file 31 — Figure S31: Product ion mass spectrum of the ion of mz 327. [file JMS-60-e5173-s058.jpg]

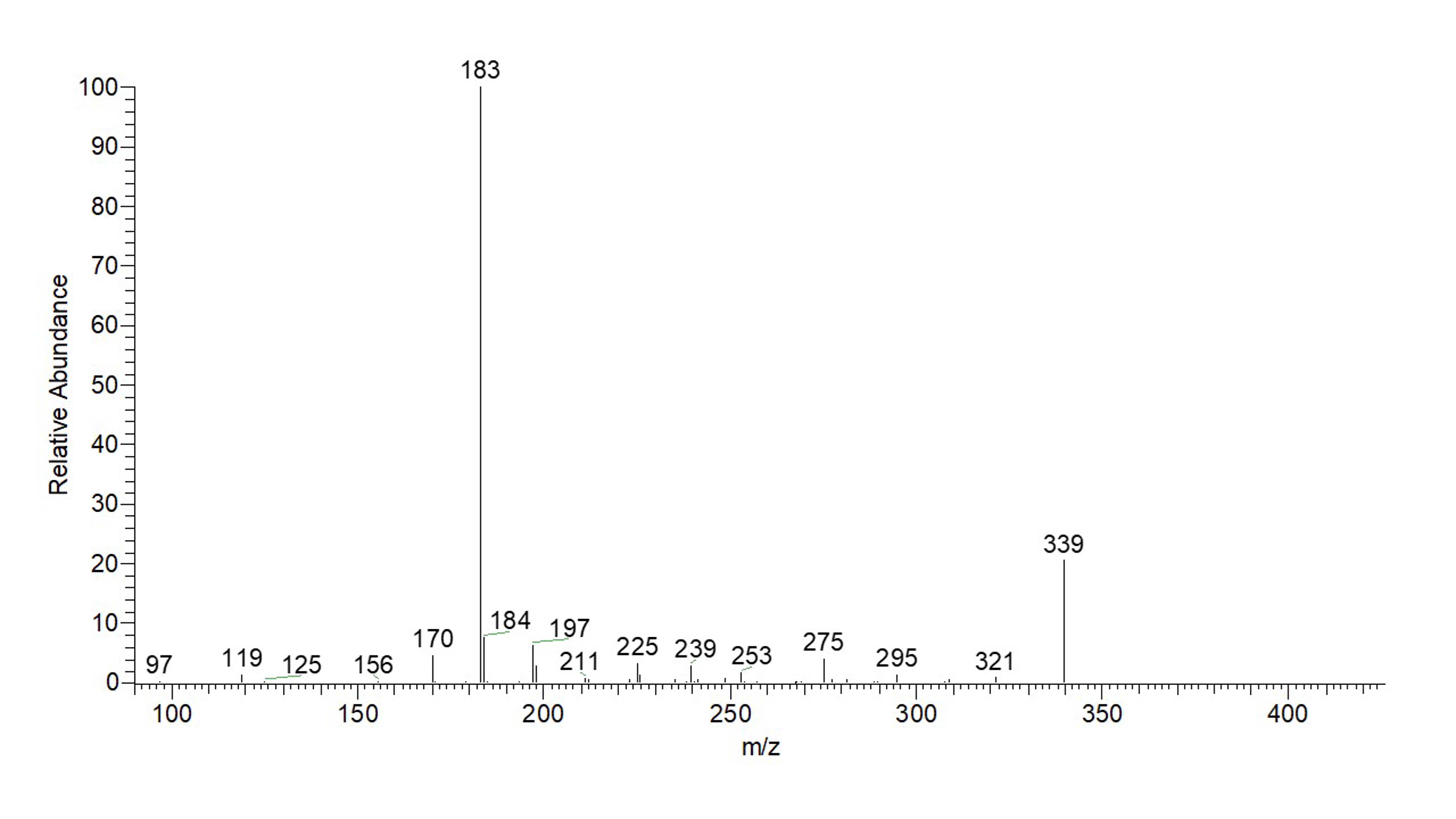

Supplement: Supplementary file 32 — Figure S32: Product ion mass spectrum of the ion of mz 339. [file JMS-60-e5173-s033.jpg]

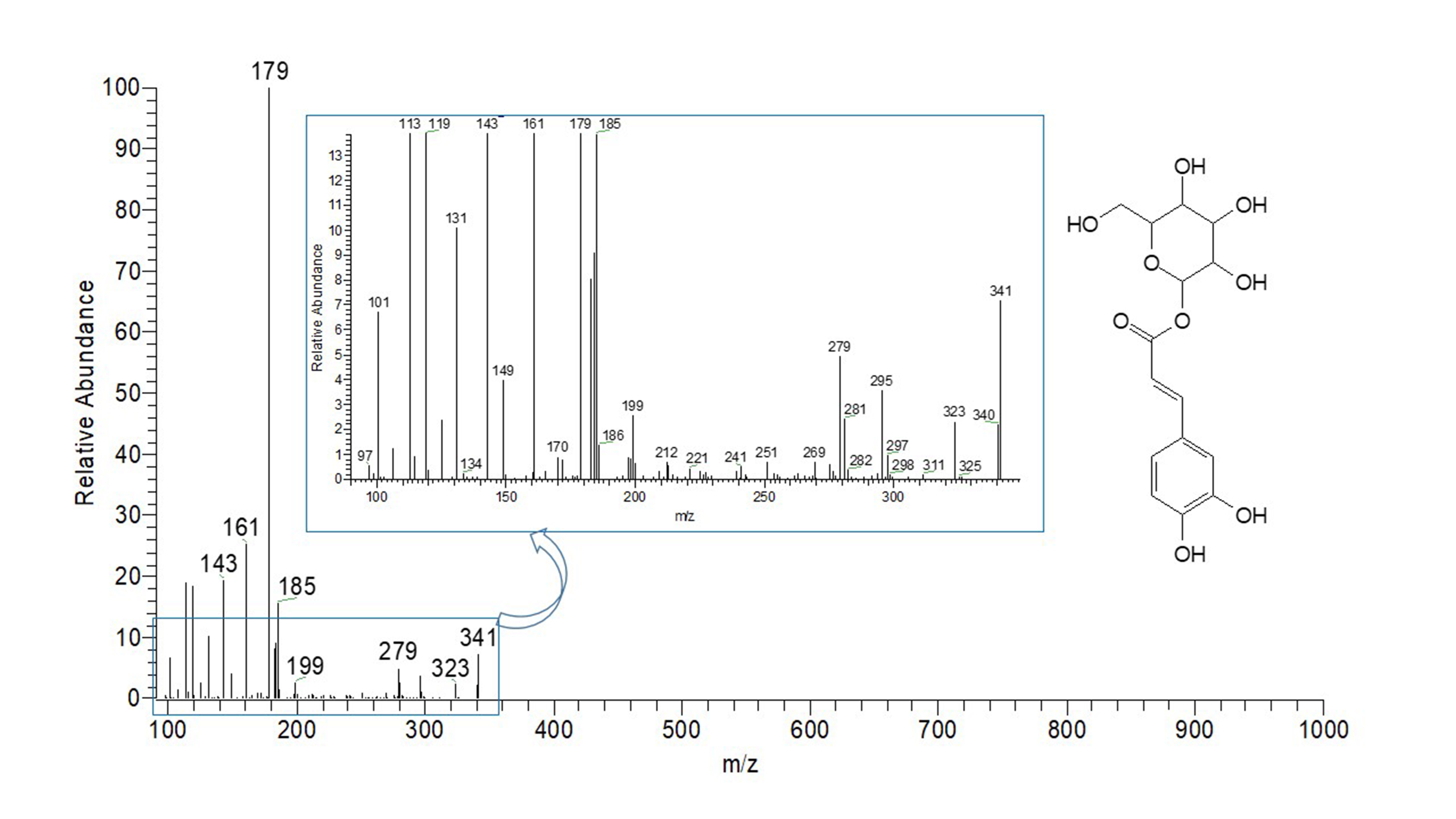

Supplement: Supplementary file 33 — Figure S33: Product ion mass spectrum of the ion of mz 341. [file JMS-60-e5173-s006.jpg]

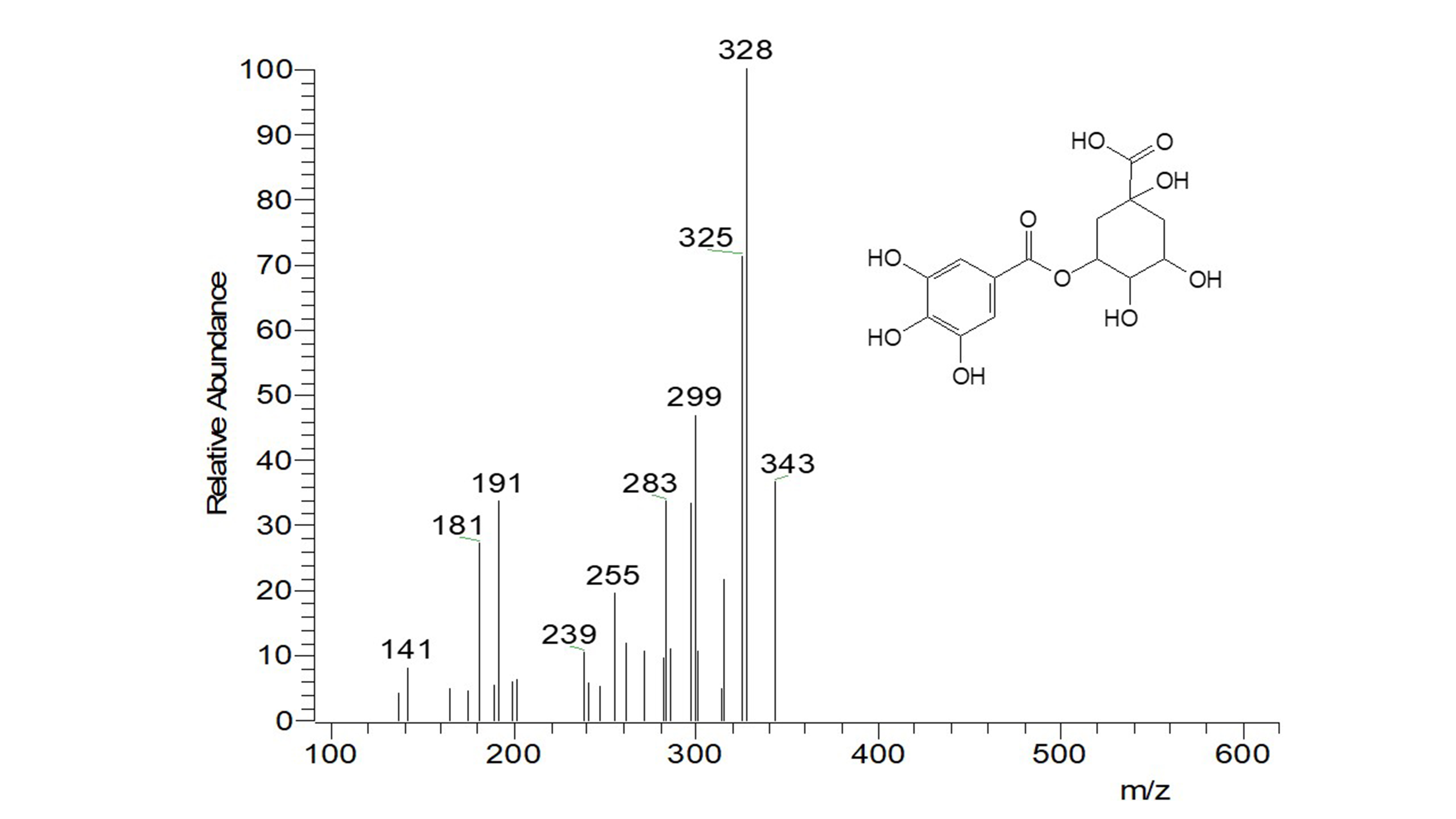

Supplement: Supplementary file 34 — Figure S34: Product ion mass spectrum of the ion of mz 343. [file JMS-60-e5173-s023.jpg]

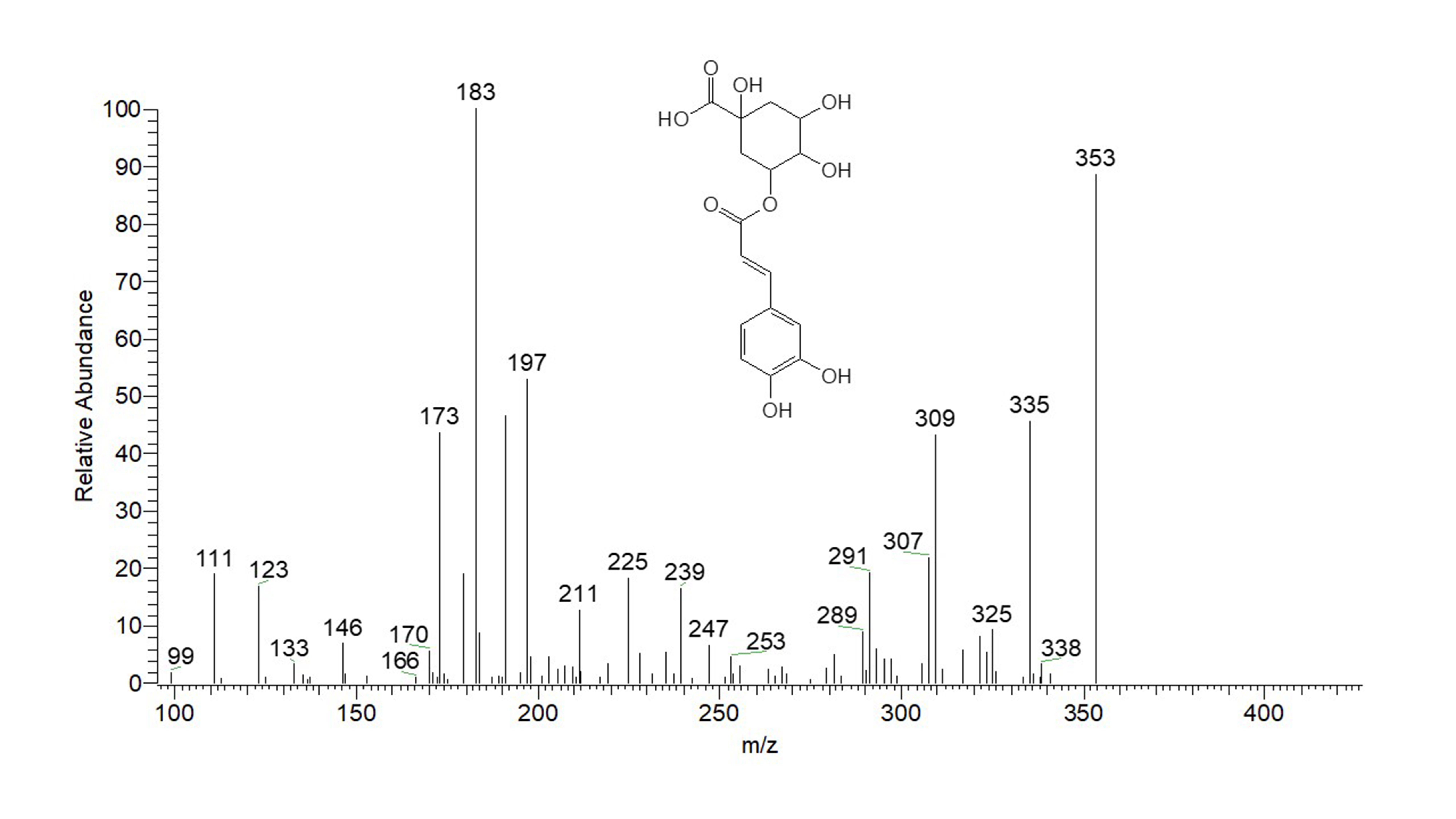

Supplement: Supplementary file 35 — Figure S35: Product ion mass spectrum of the ion of mz 353. [file JMS-60-e5173-s048.jpg]

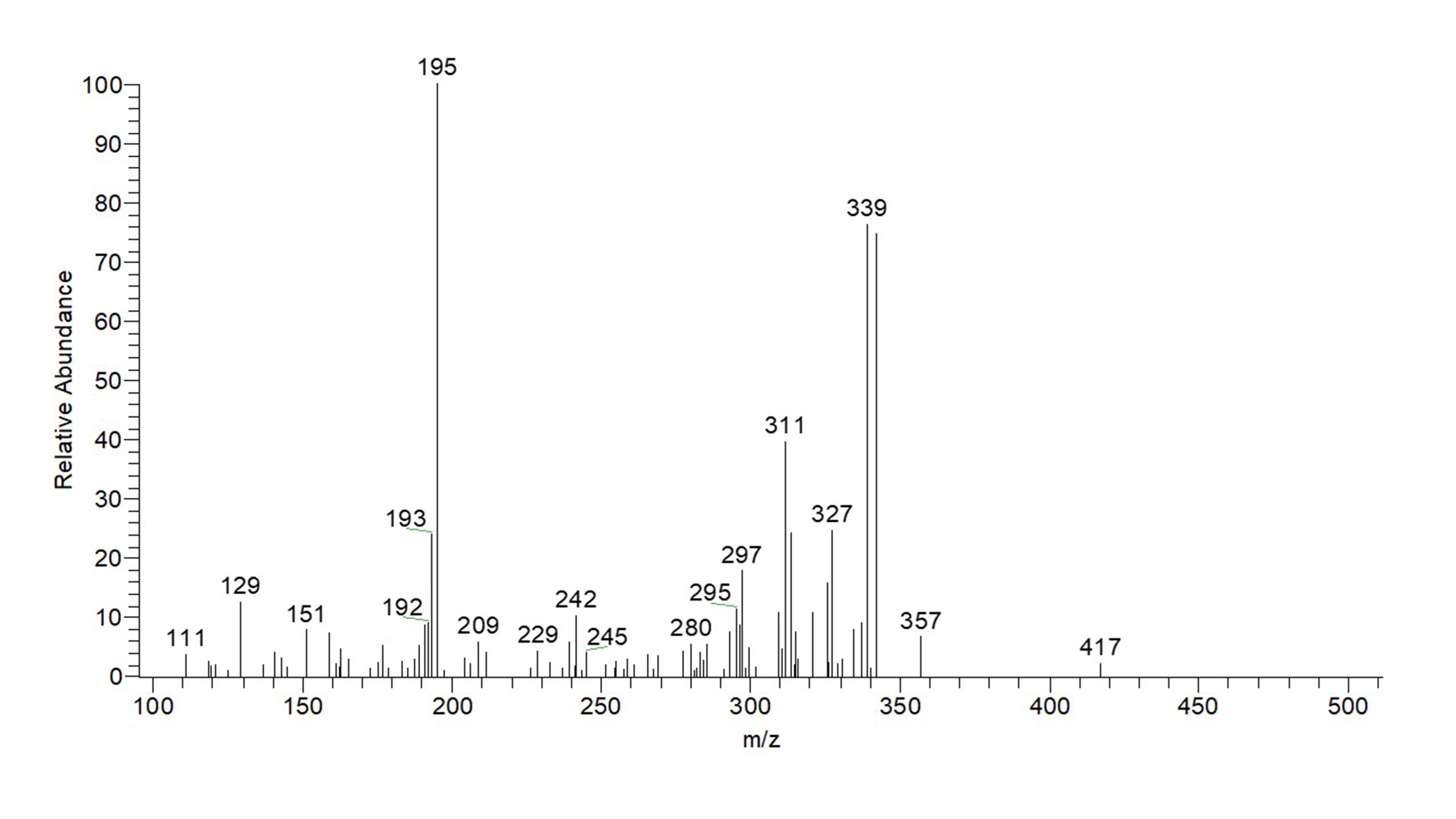

Supplement: Supplementary file 36 — Figure S36: Product ion mass spectrum of the ion of mz 357. [file JMS-60-e5173-s040.jpg]

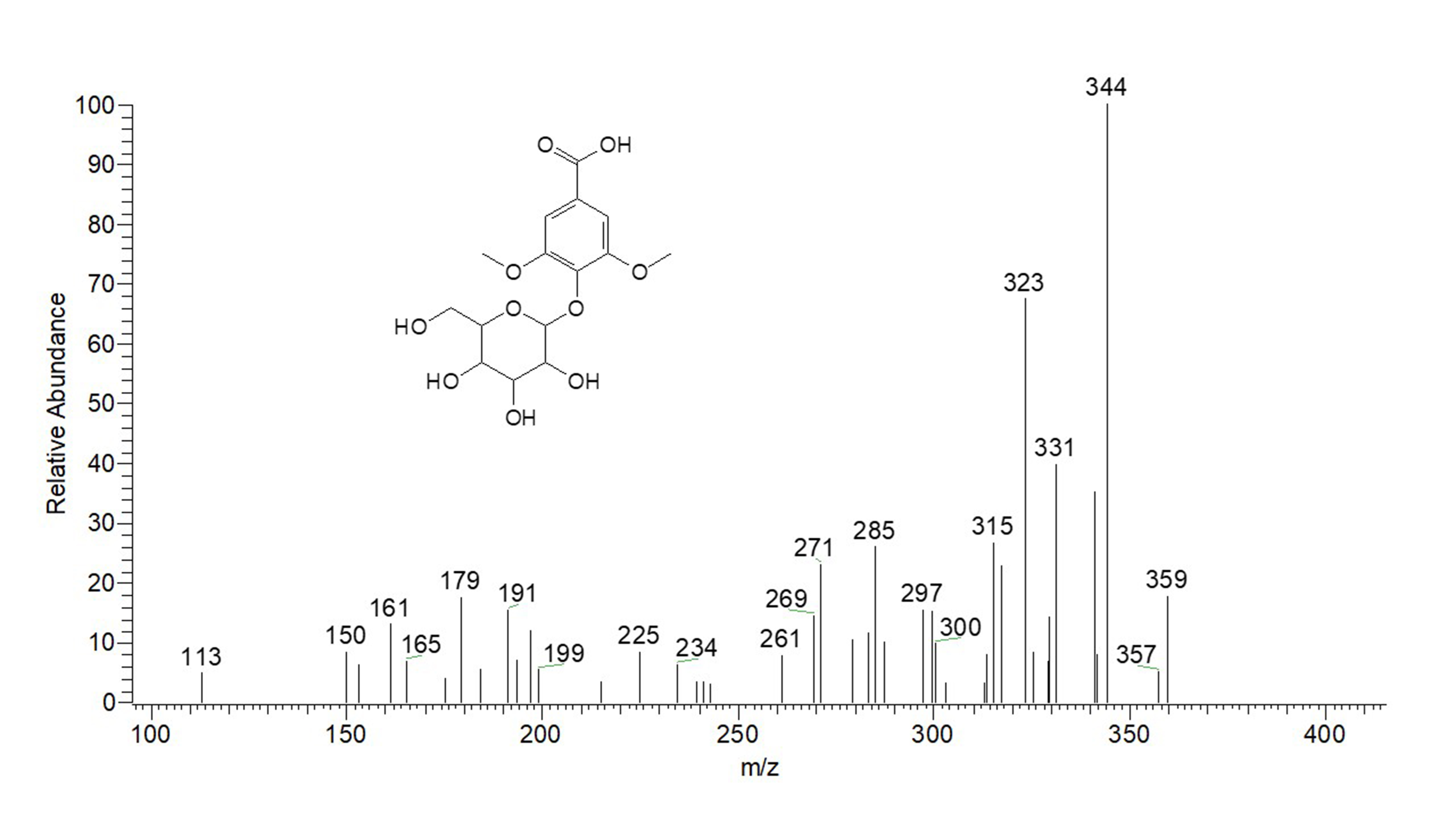

Supplement: Supplementary file 37 — Figure S37: Product ion mass spectrum of the ion of mz 359. [file JMS-60-e5173-s013.jpg]

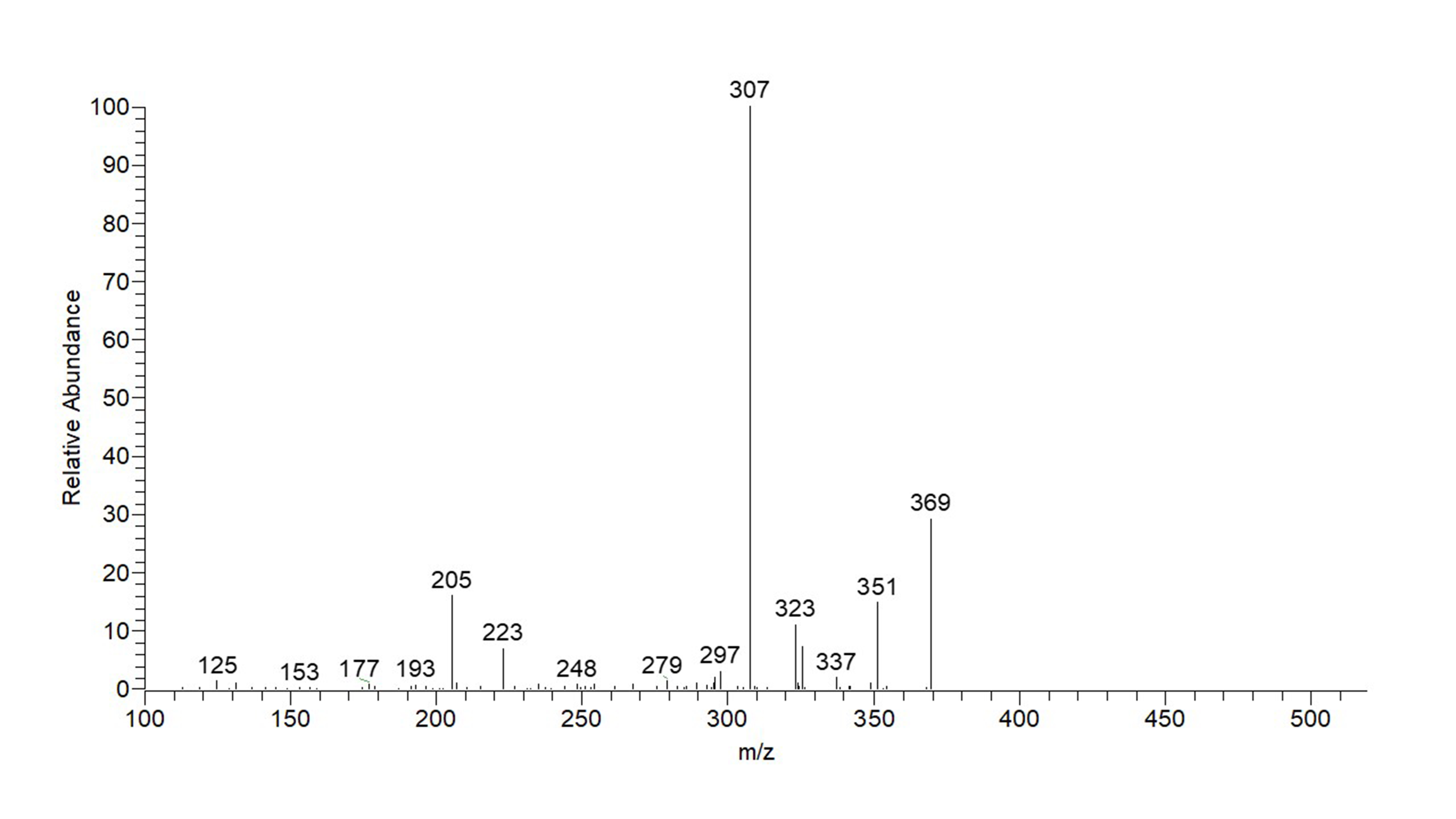

Supplement: Supplementary file 38 — Figure S38: Product ion mass spectrum of the ion of mz 369. [file JMS-60-e5173-s014.jpg]

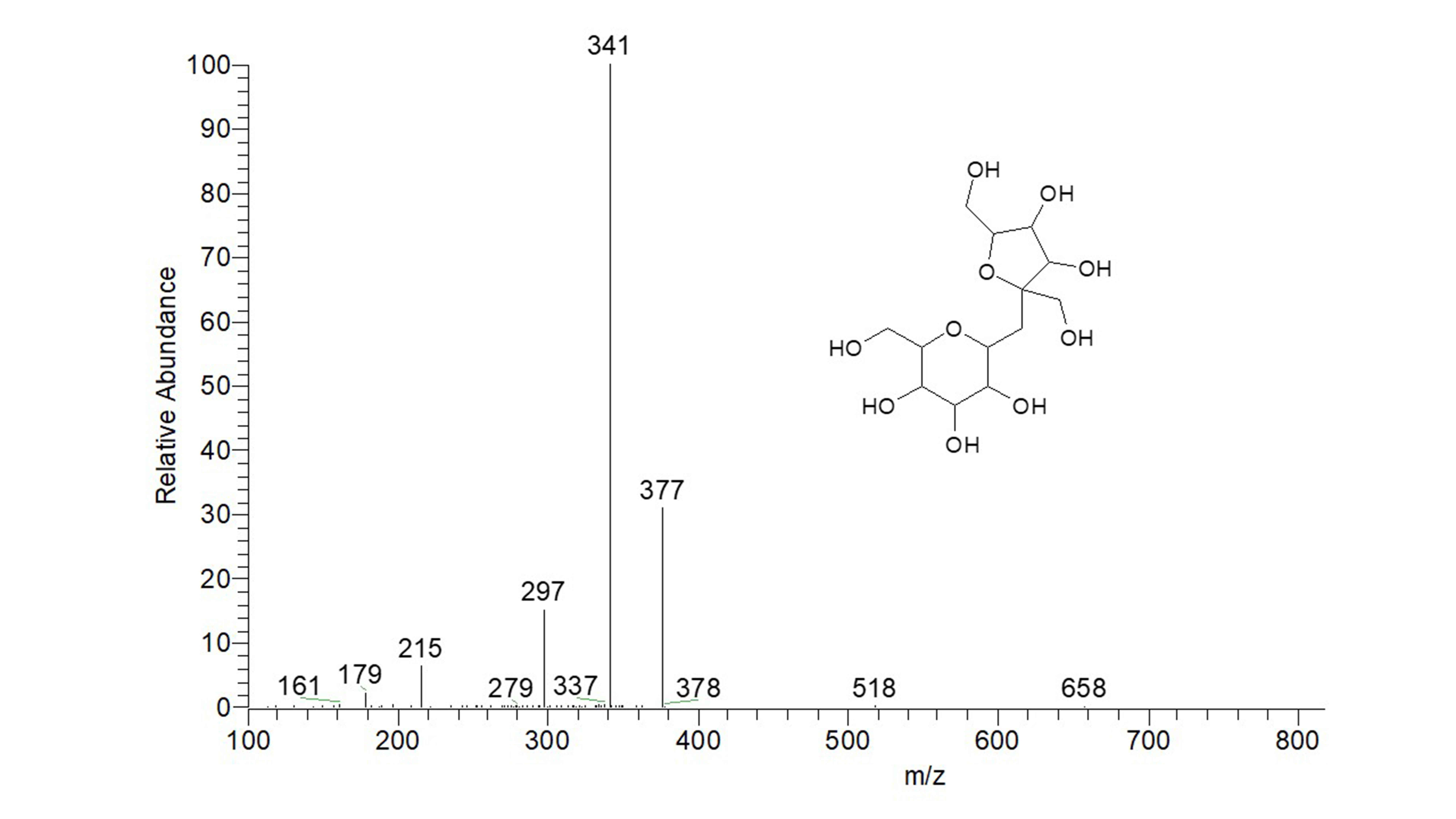

Supplement: Supplementary file 39 — Figure S39: Product ion mass spectrum of the ion of mz 377. [file JMS-60-e5173-s042.jpg]

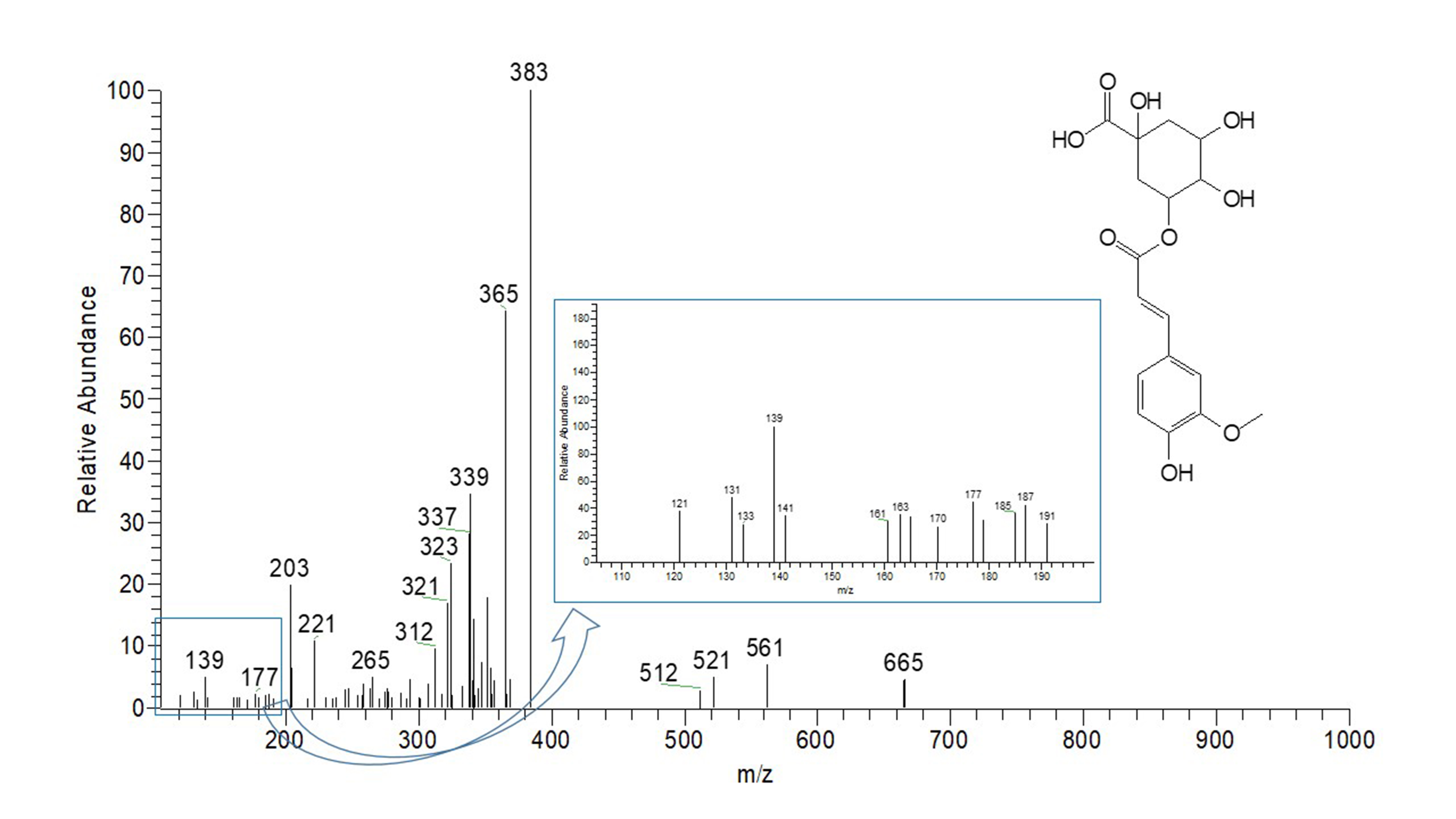

Supplement: Supplementary file 40 — Figure S40: Product ion mass spectrum of the ion of mz 383. [file JMS-60-e5173-s016.jpg]

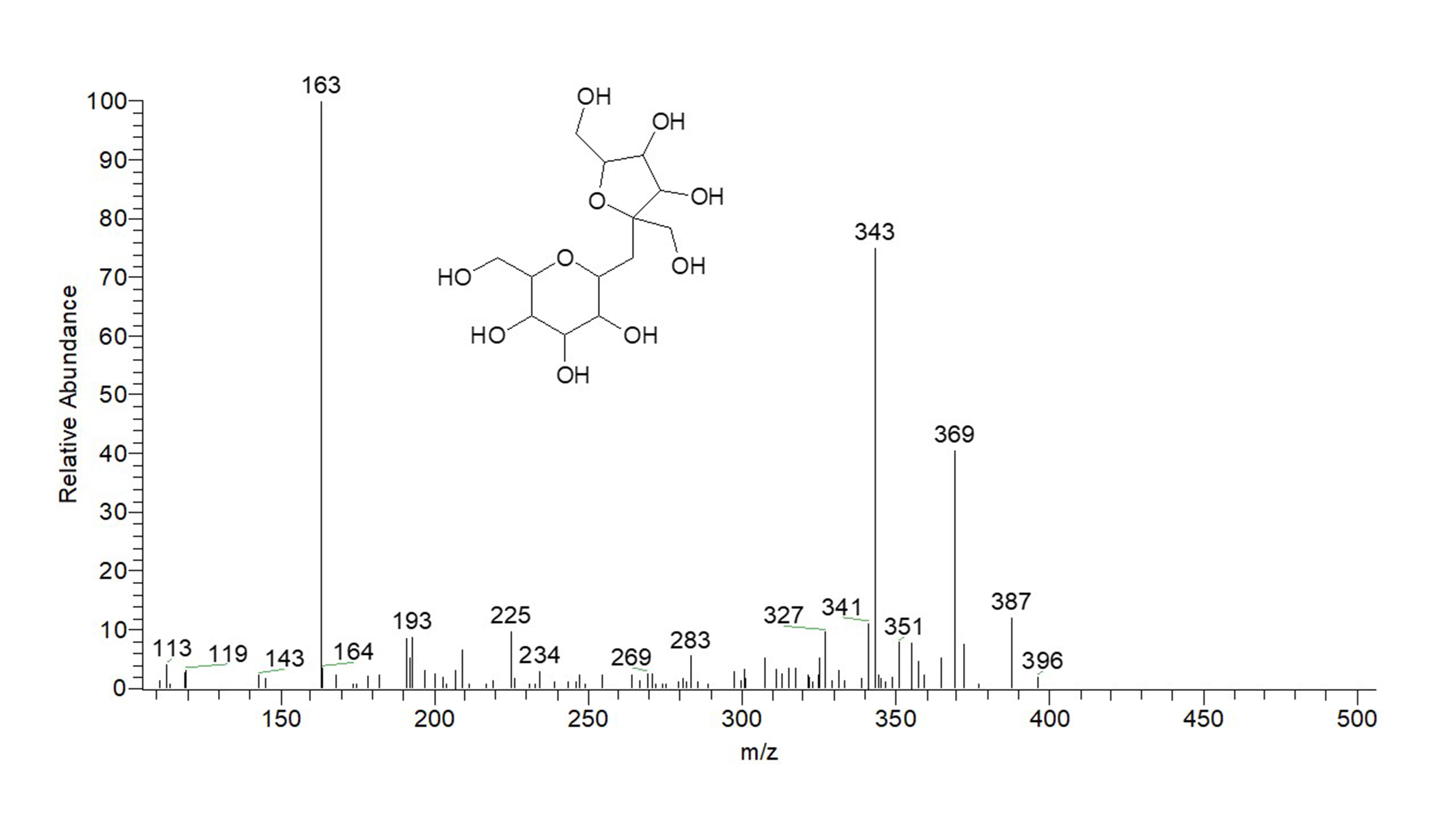

Supplement: Supplementary file 41 — Figure S41: Product ion mass spectrum of the ion of mz 387. [file JMS-60-e5173-s051.jpg]

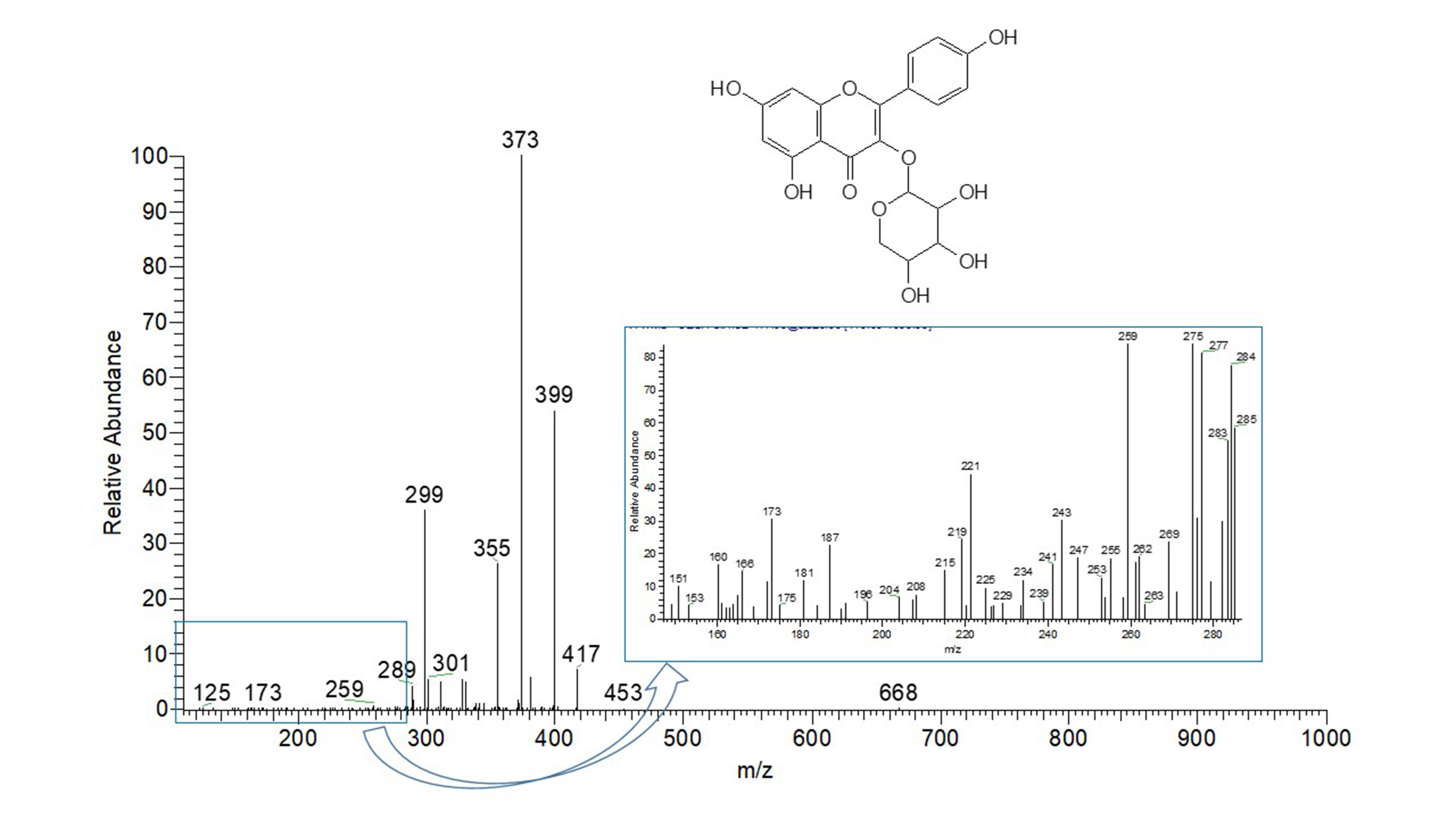

Supplement: Supplementary file 42 — Figure S42: Product ion mass spectrum of the ion of mz 417. [file JMS-60-e5173-s044.jpg]

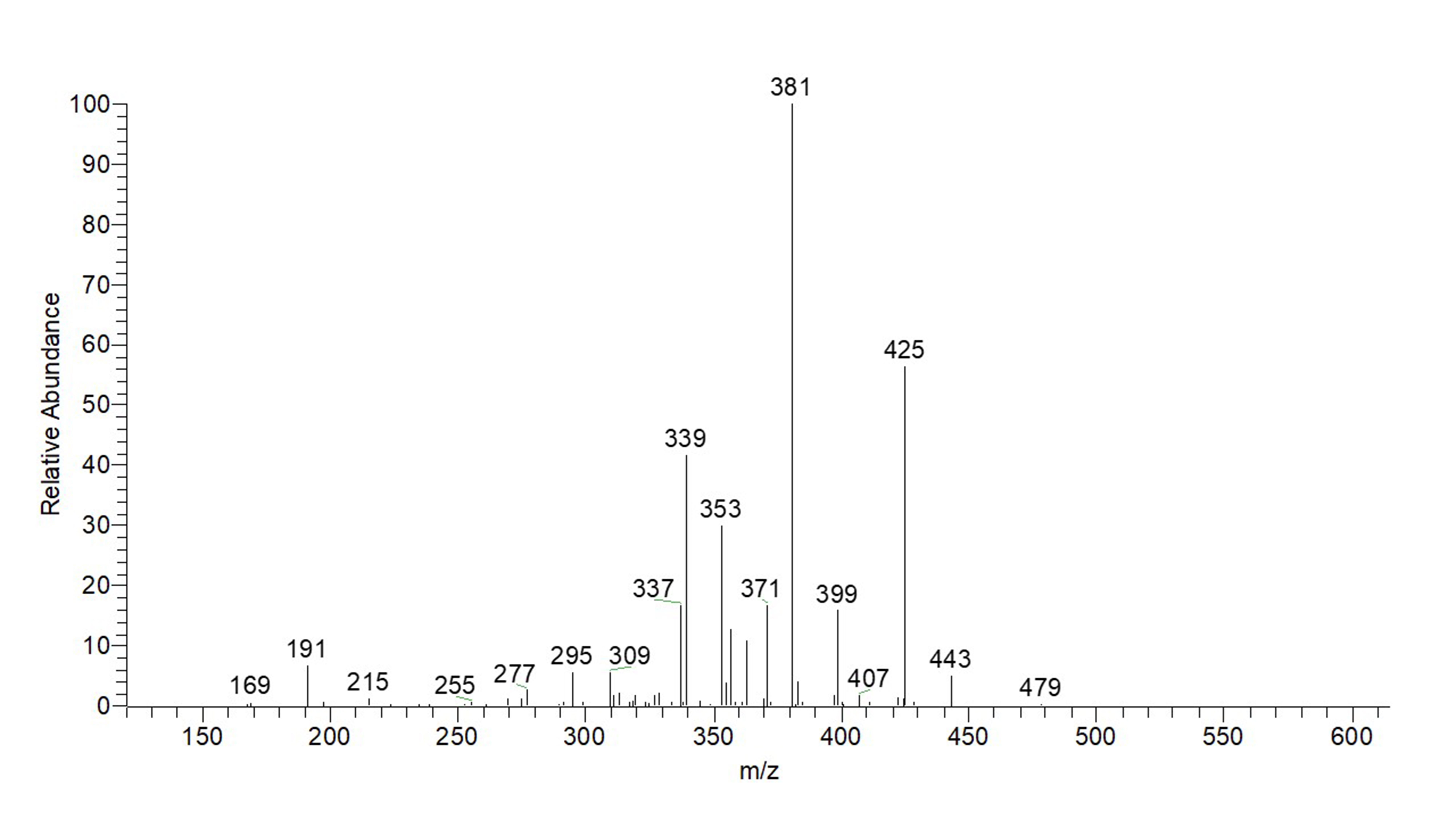

Supplement: Supplementary file 43 — Figure S43: Product ion mass spectrum of the ion of mz 443. [file JMS-60-e5173-s010.jpg]

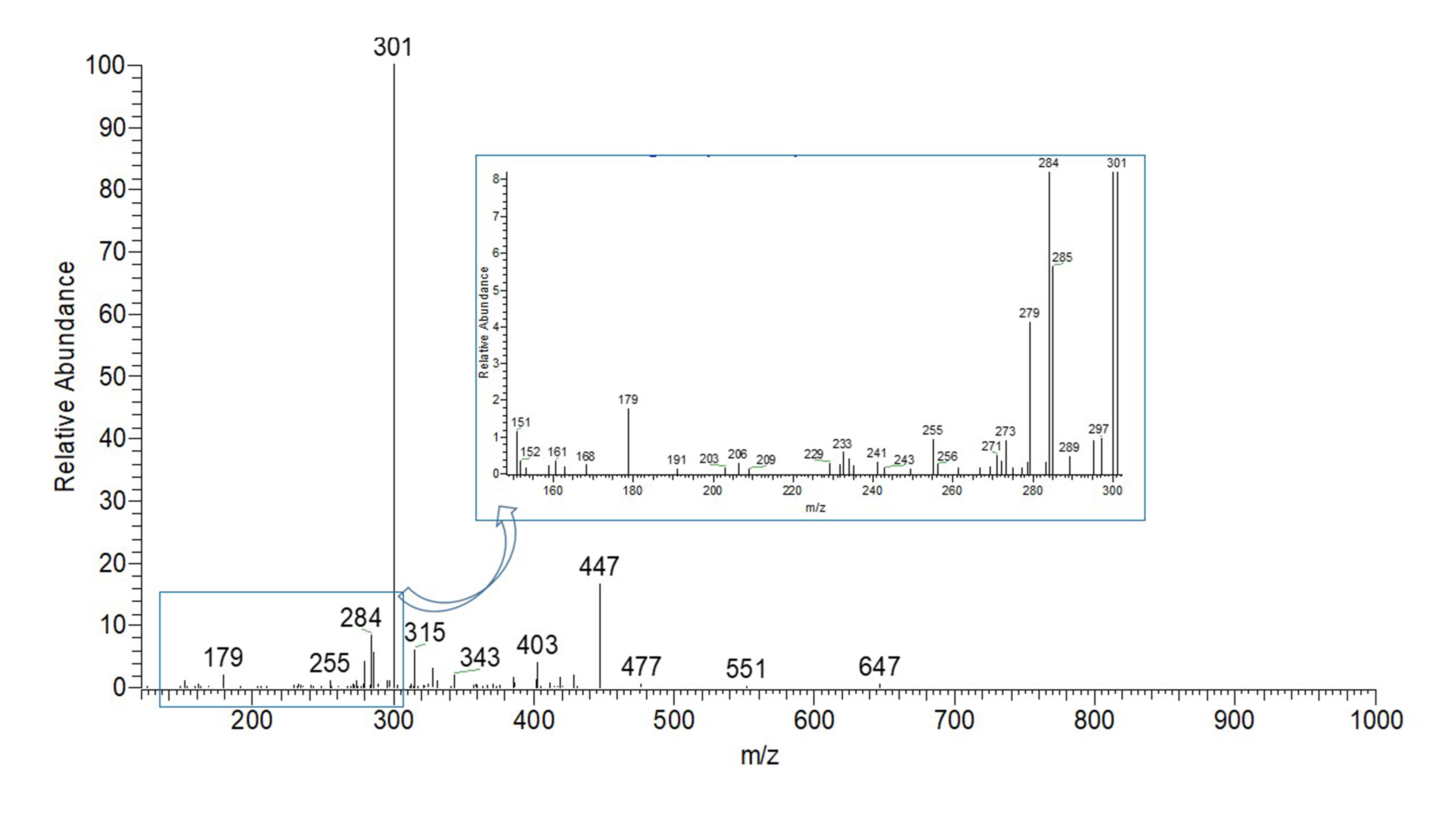

Supplement: Supplementary file 44 — Figure S44: Product ion mass spectrum of the ion of mz 447. [file JMS-60-e5173-s025.jpg]

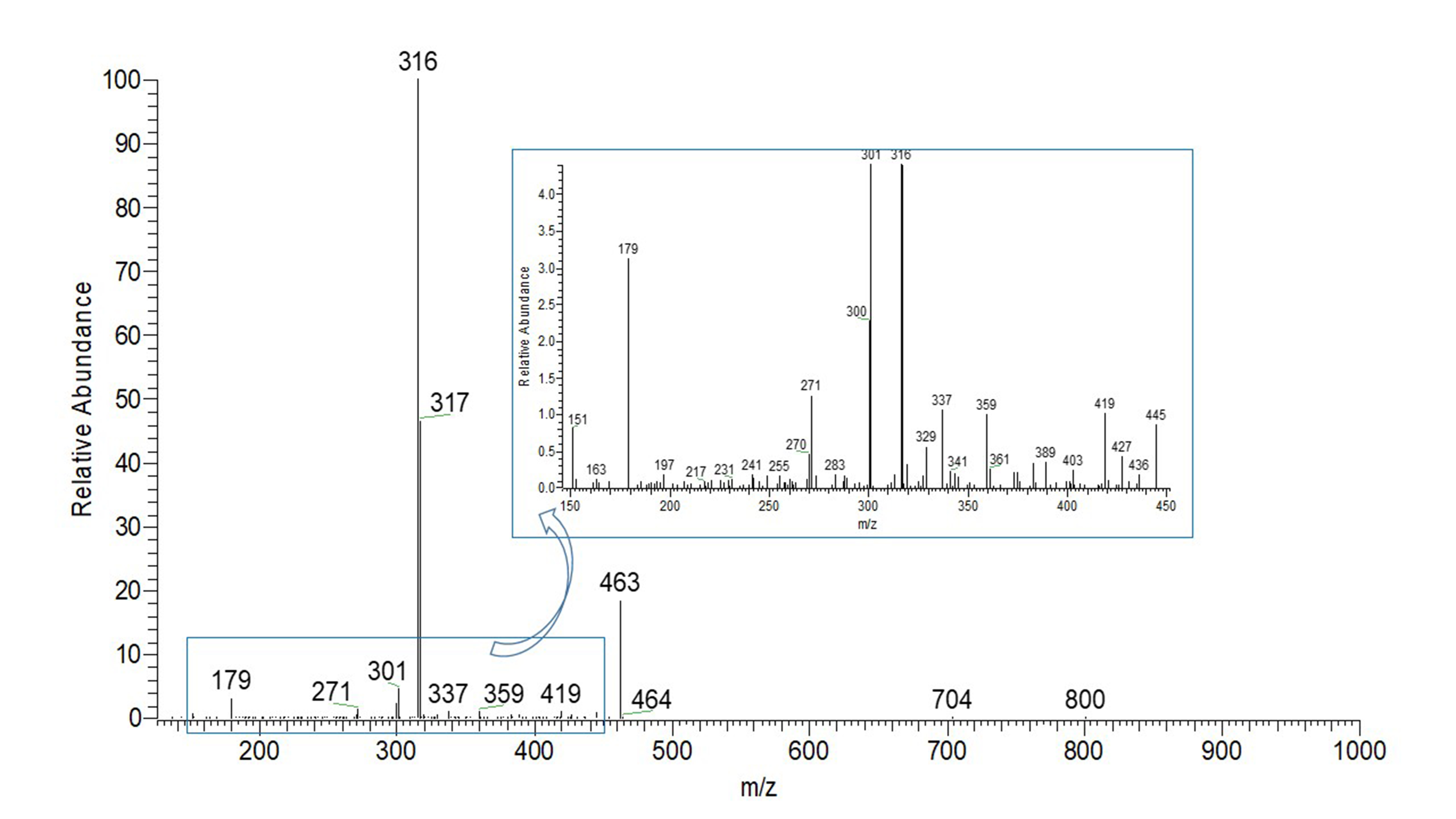

Supplement: Supplementary file 45 — Figure S45: Product ion mass spectrum of the ion of mz 463. [file JMS-60-e5173-s060.jpg]

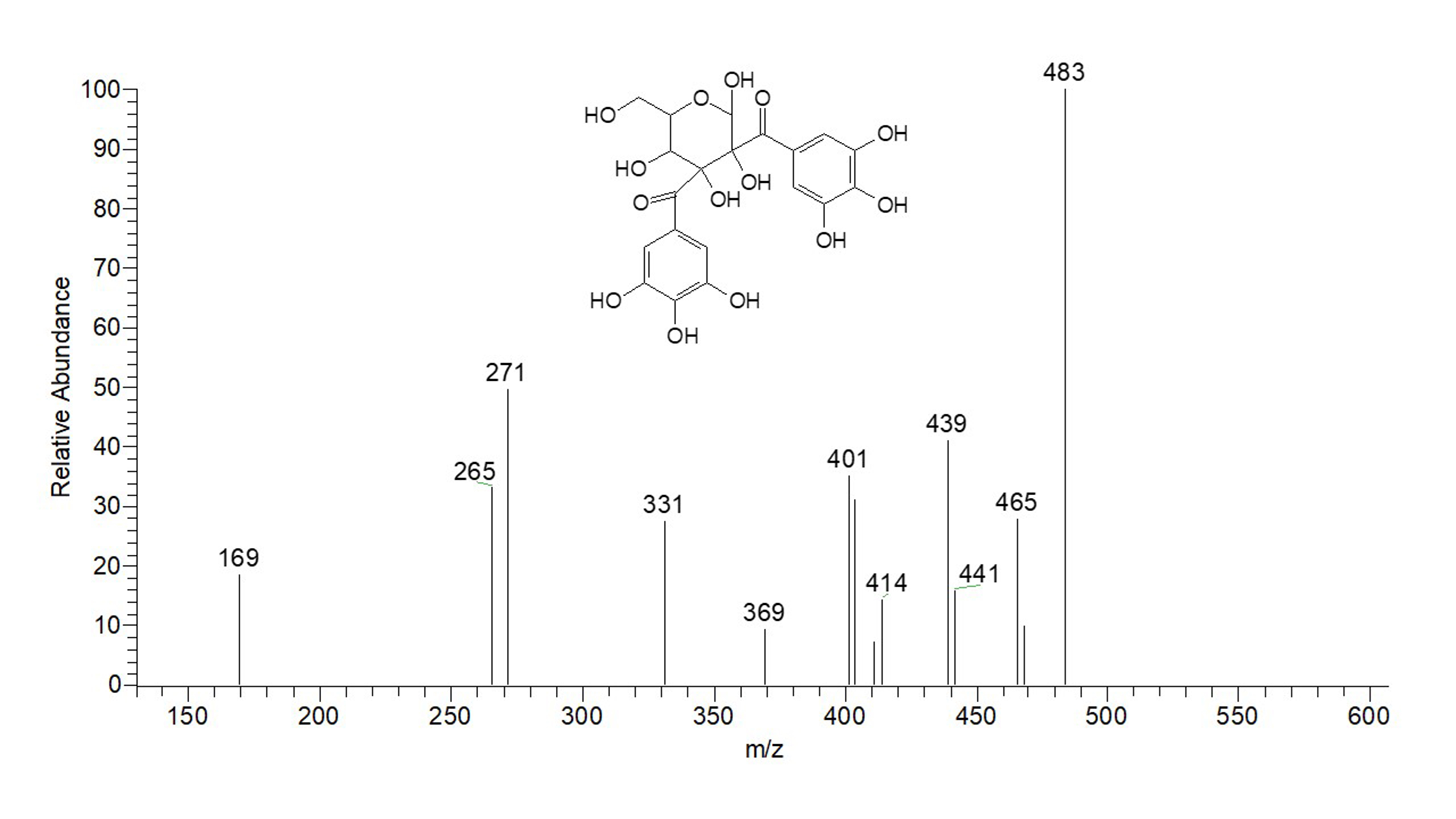

Supplement: Supplementary file 46 — Figure S46: Product ion mass spectrum of the ion of mz 483. [file JMS-60-e5173-s037.jpg]

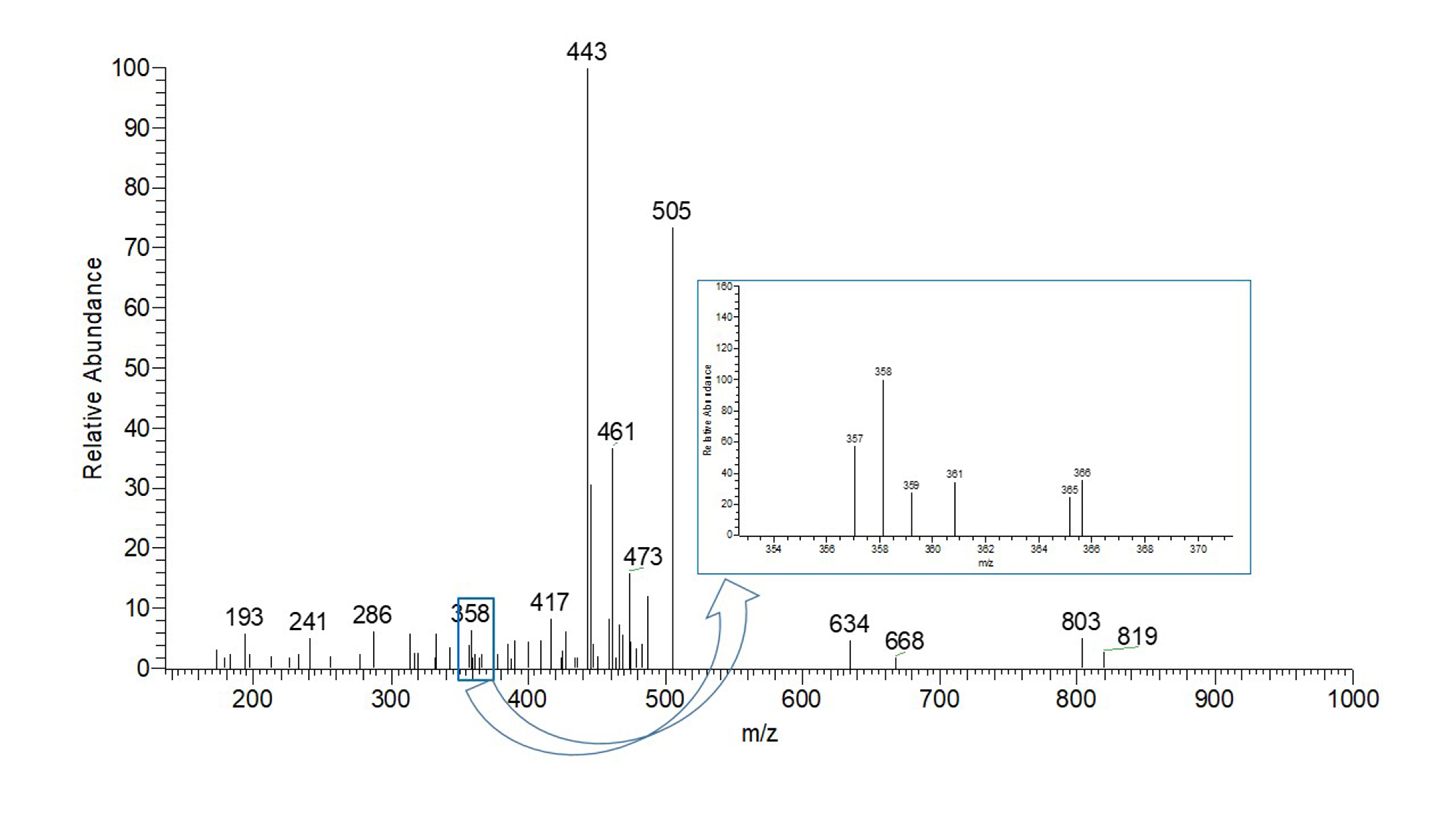

Supplement: Supplementary file 47 — Figure S47: Product ion mass spectrum of the ion of mz 505. [file JMS-60-e5173-s004.jpg]

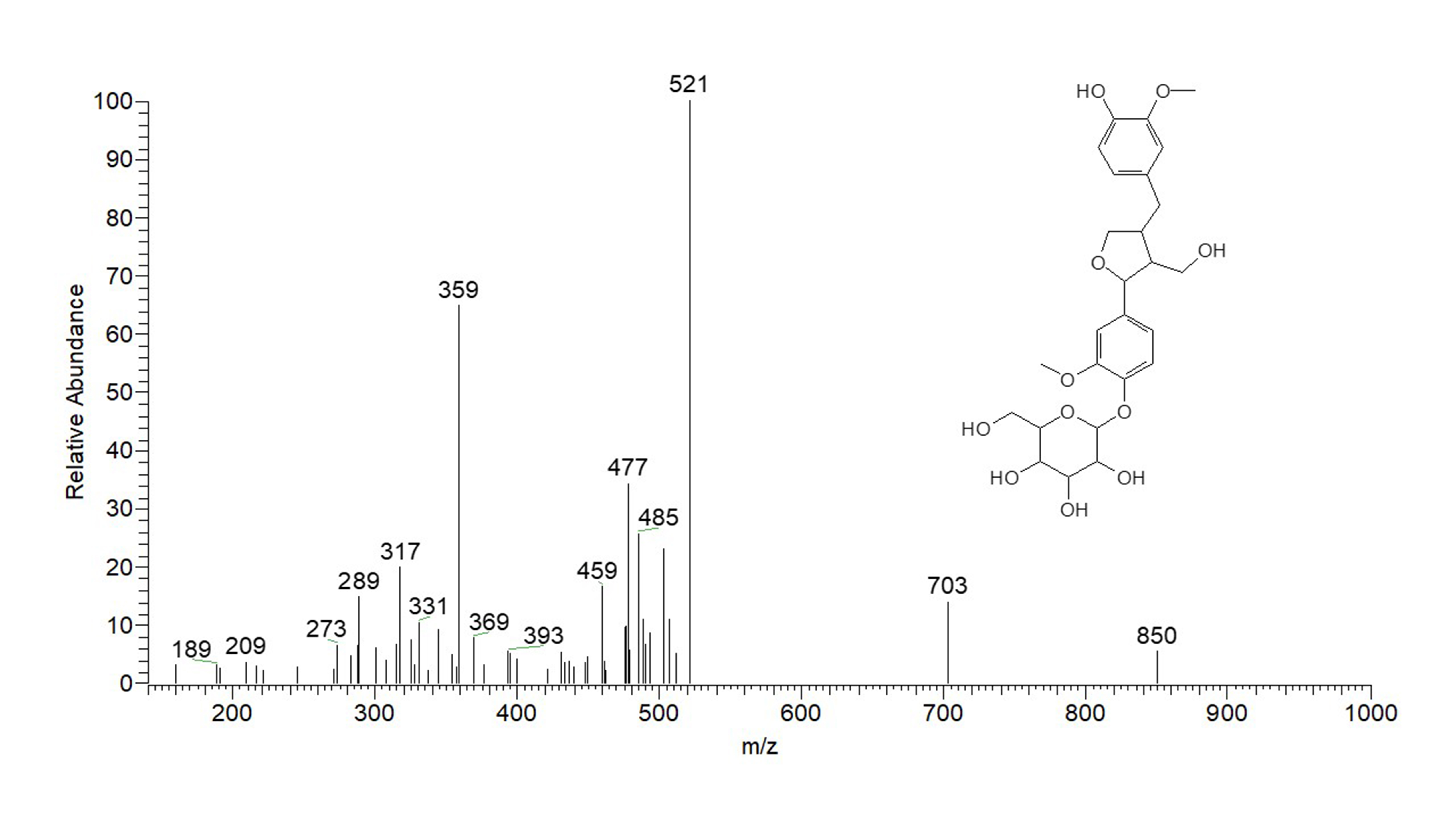

Supplement: Supplementary file 48 — Figure S48: Product ion mass spectrum of the ion of mz 521. [file JMS-60-e5173-s002.jpg]

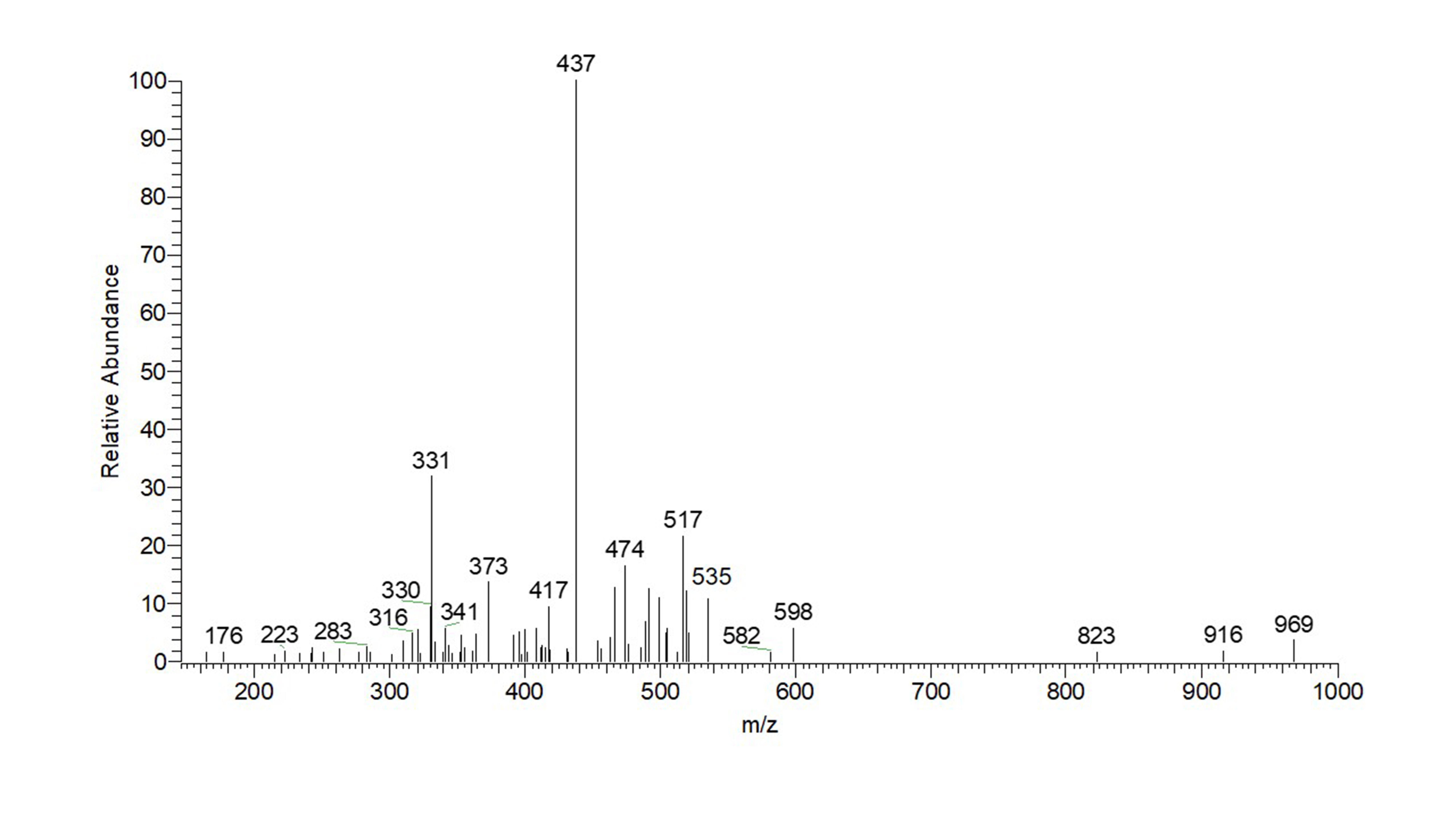

Supplement: Supplementary file 49 — Figure S49: Product ion mass spectrum of the ion of mz 535. [file JMS-60-e5173-s036.jpg]

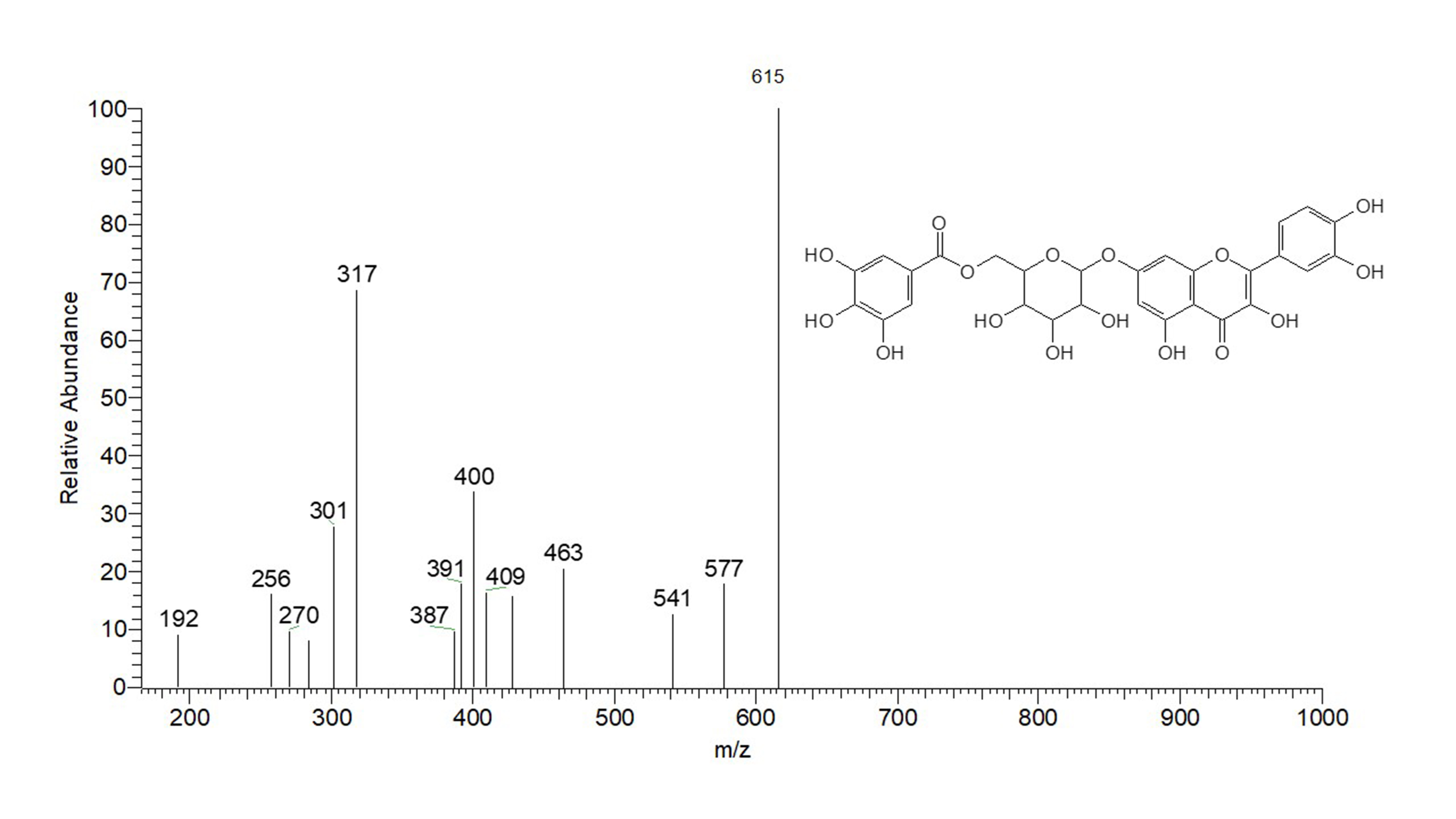

Supplement: Supplementary file 50 — Figure S50: Product ion mass spectrum of the ion of mz 615. [file JMS-60-e5173-s003.jpg]

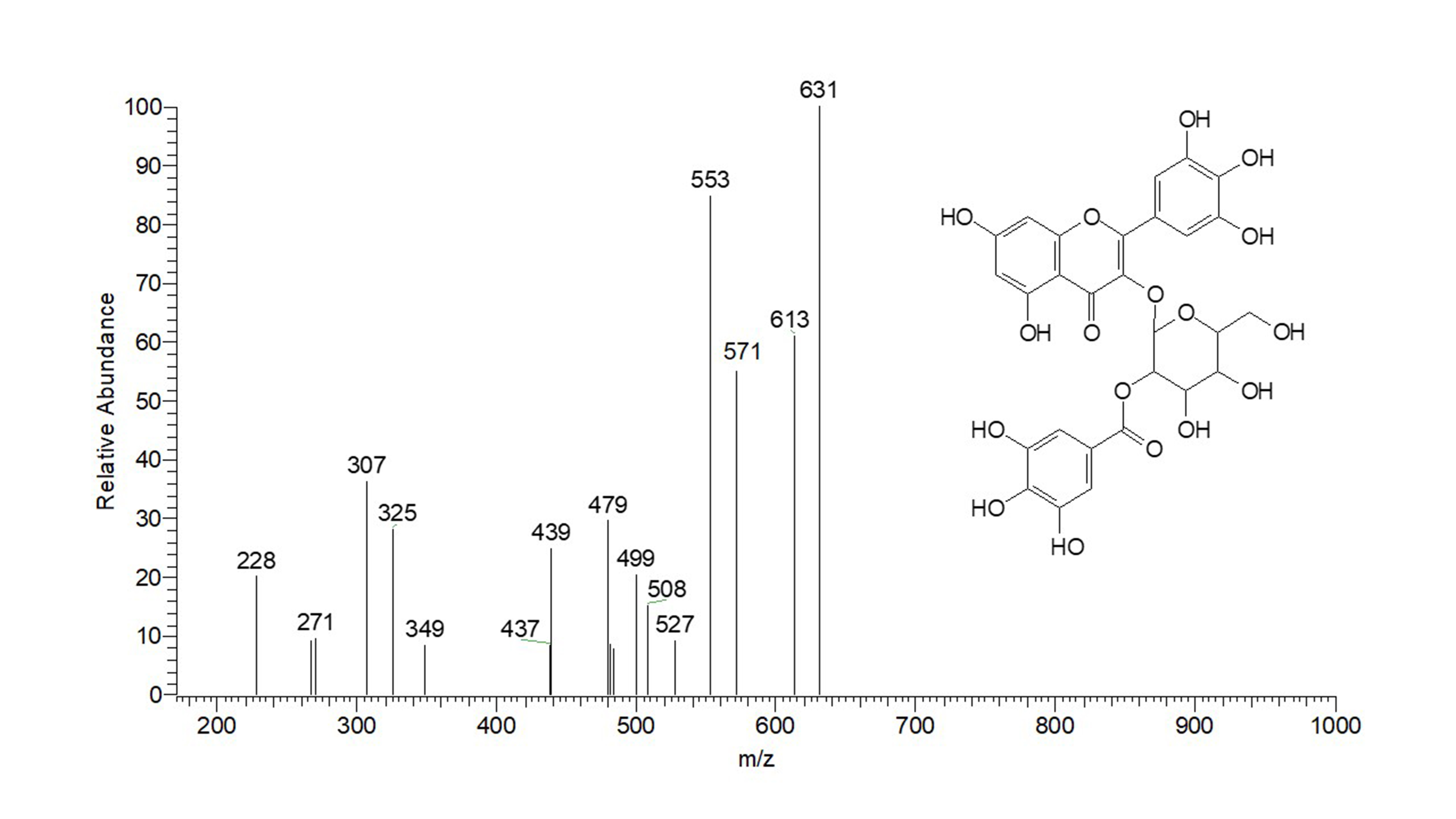

Supplement: Supplementary file 51 — Figure S51: Product ion mass spectrum of the ion of mz 631. [file JMS-60-e5173-s038.jpg]

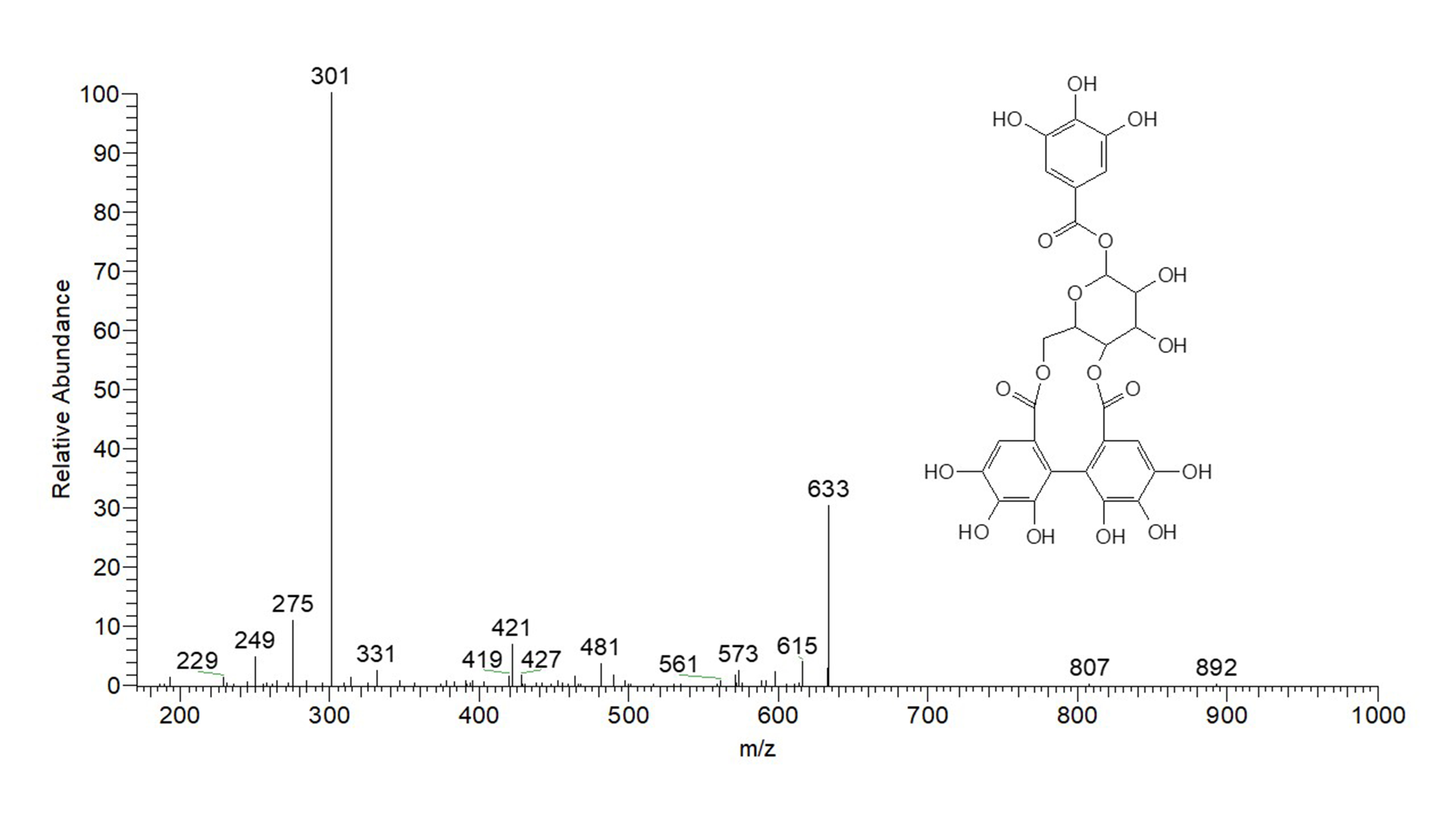

Supplement: Supplementary file 52 — Figure S52: Product ion mass spectrum of the ion of mz 633. [file JMS-60-e5173-s059.jpg]

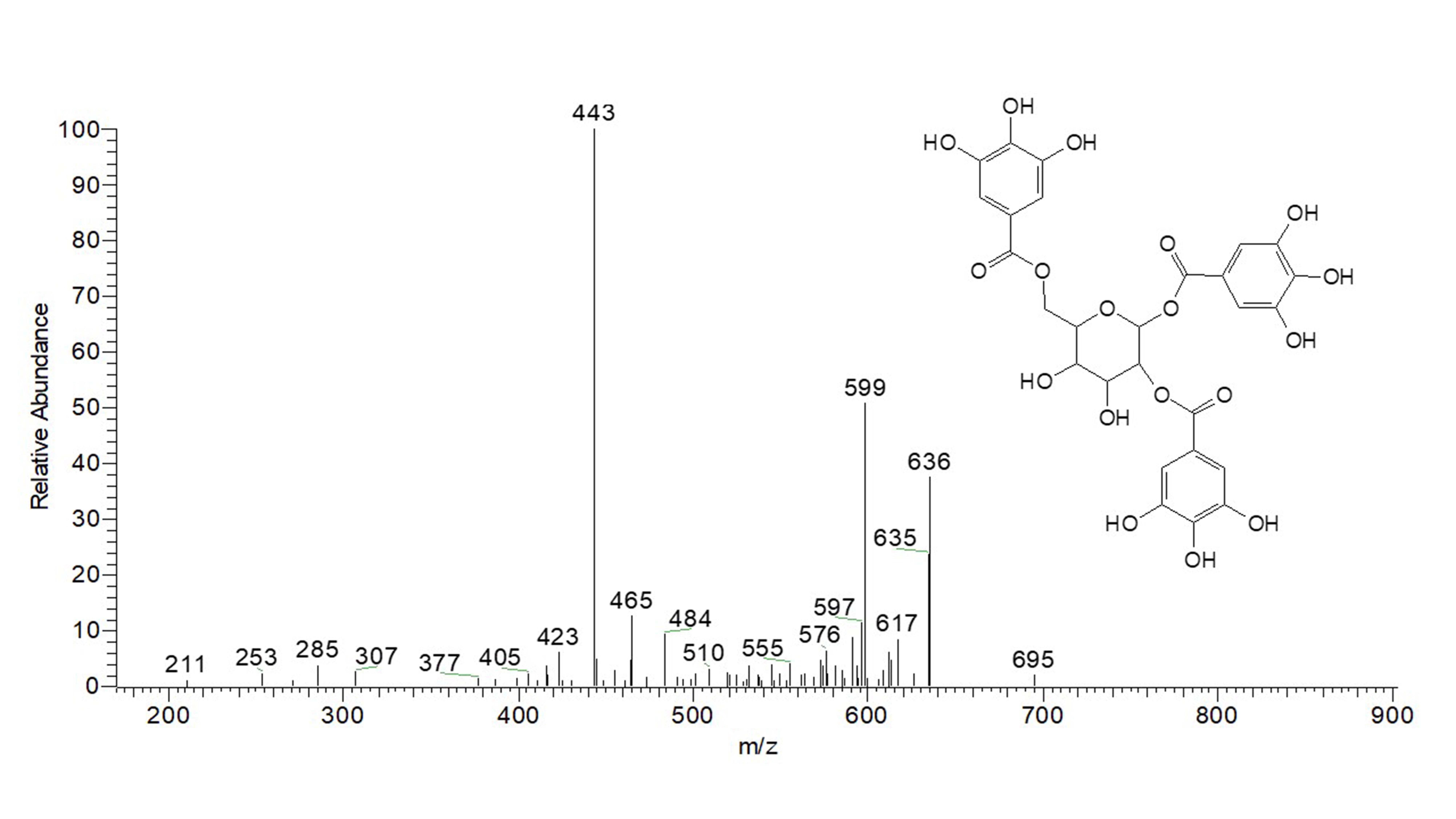

Supplement: Supplementary file 53 — Figure S53: Product ion mass spectrum of the ion of mz 635. [file JMS-60-e5173-s026.jpg]

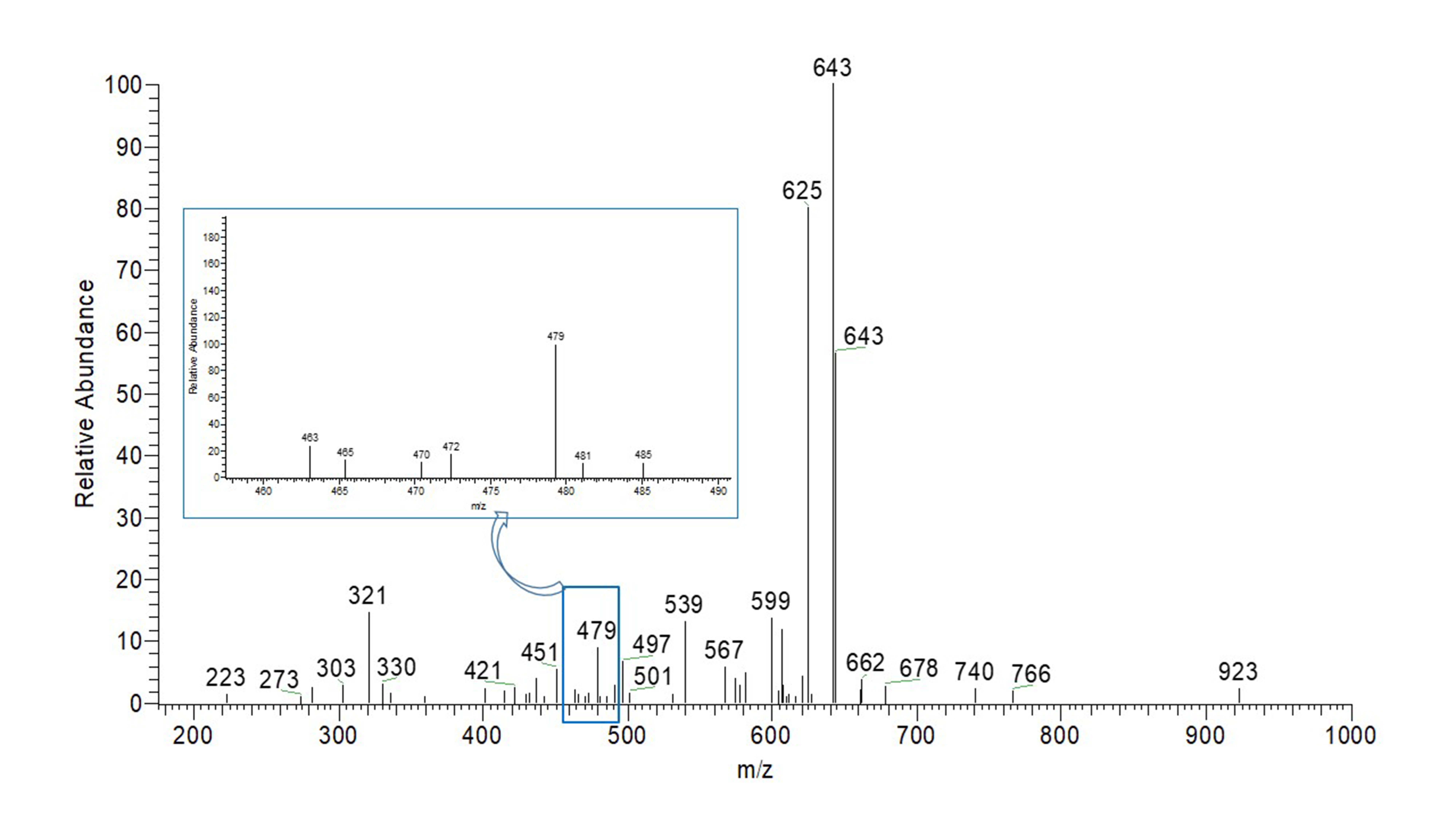

Supplement: Supplementary file 54 — Figure S54: Product ion mass spectrum of the ion of mz 643. [file JMS-60-e5173-s009.jpg]

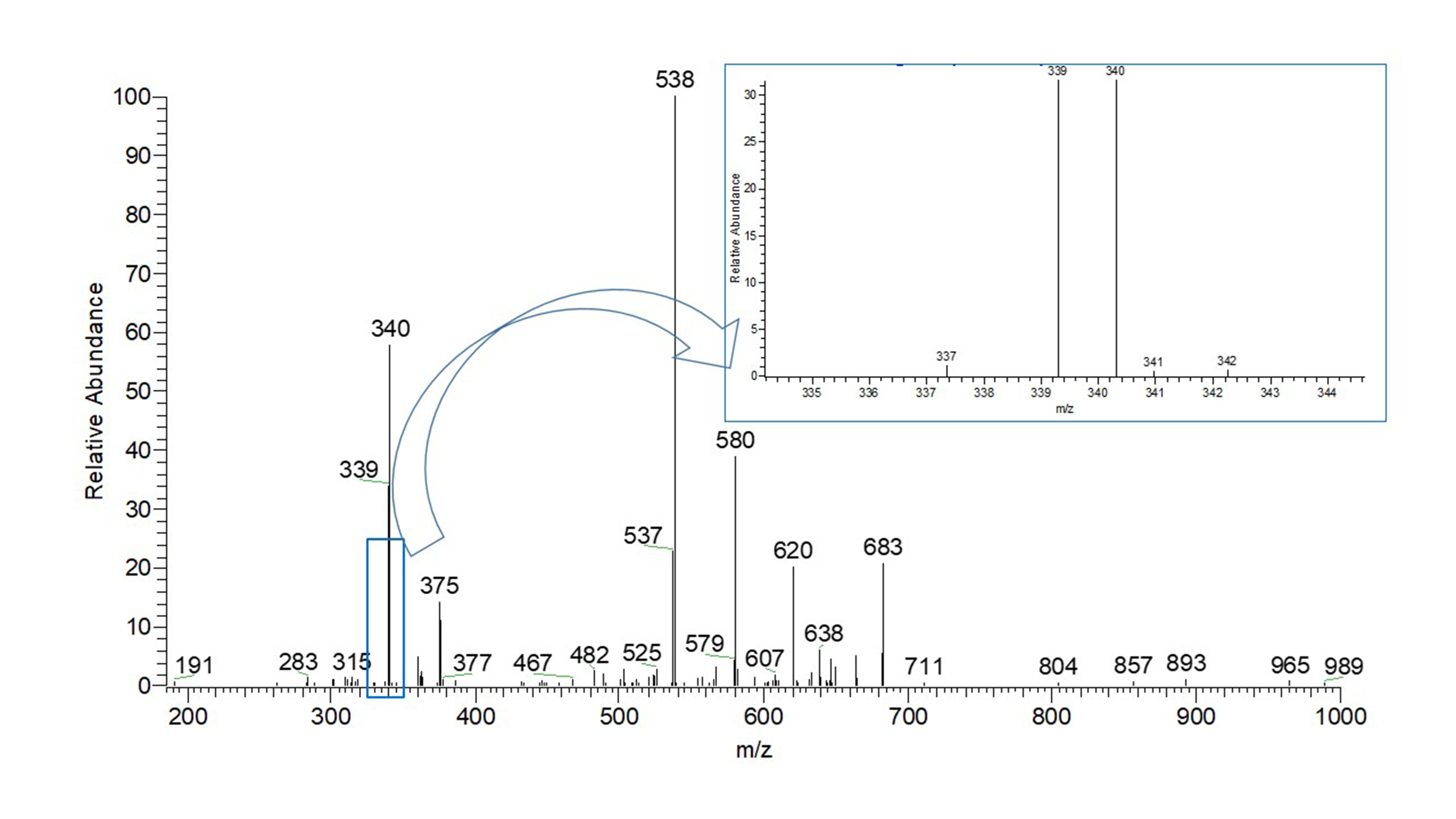

Supplement: Supplementary file 55 — Figure S55: Product ion mass spectrum of the ion of mz 683. [file JMS-60-e5173-s045.jpg]

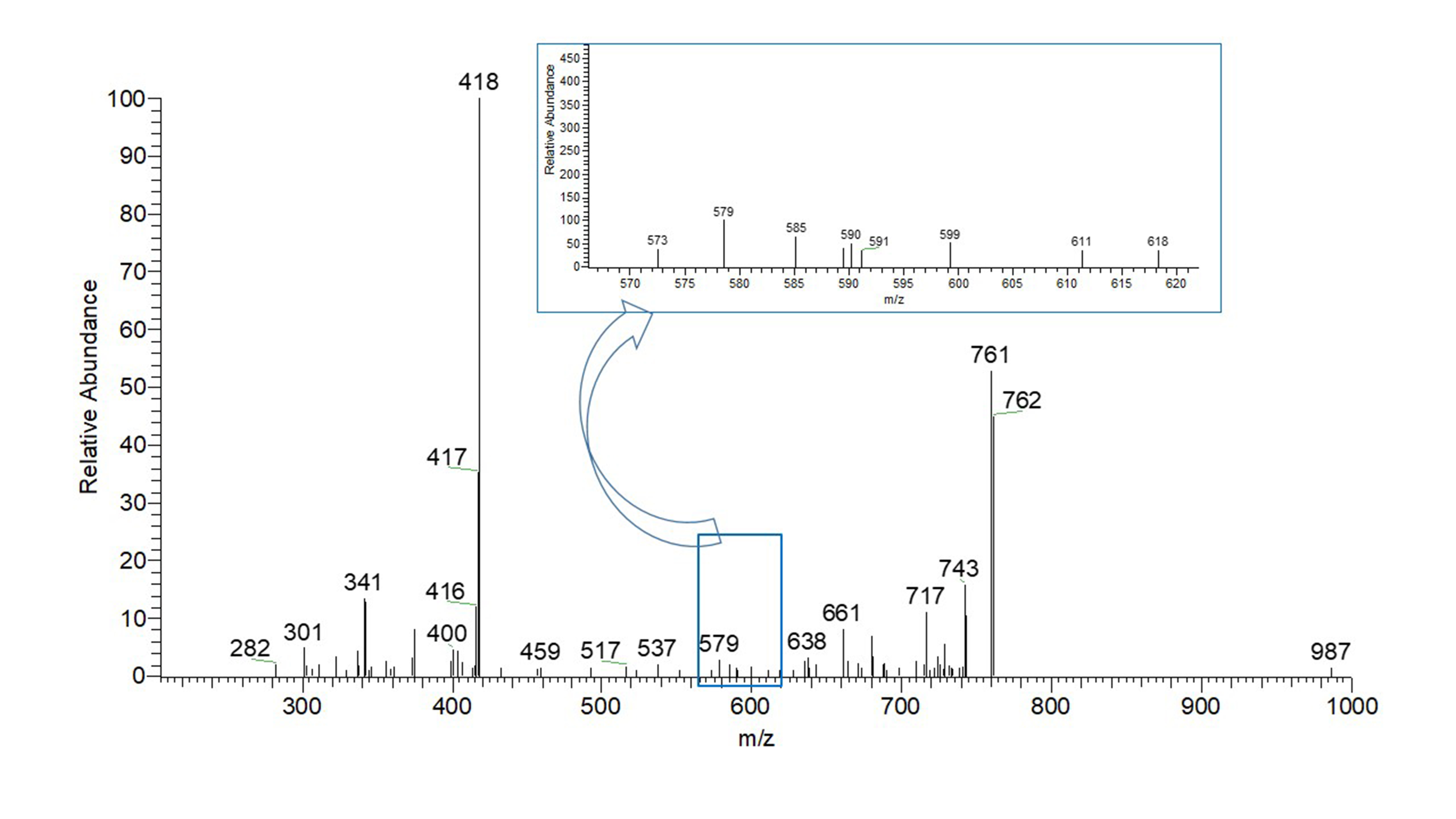

Supplement: Supplementary file 56 — Figure S56: Product ion mass spectrum of the ion of mz 761. [file JMS-60-e5173-s050.jpg]

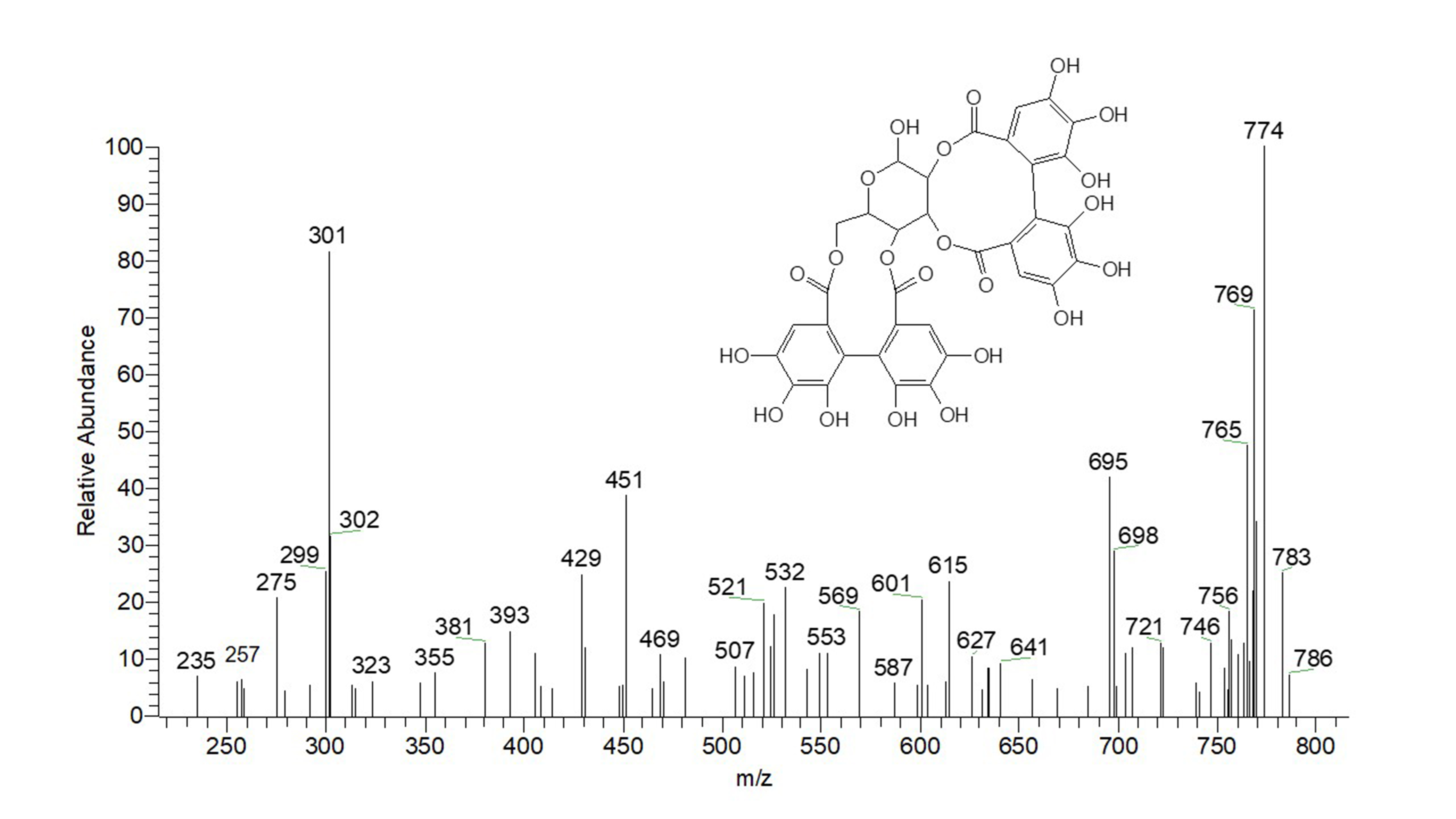

Supplement: Supplementary file 57 — Figure S57: Product ion mass spectrum of the ion of mz 783. [file JMS-60-e5173-s018.jpg]

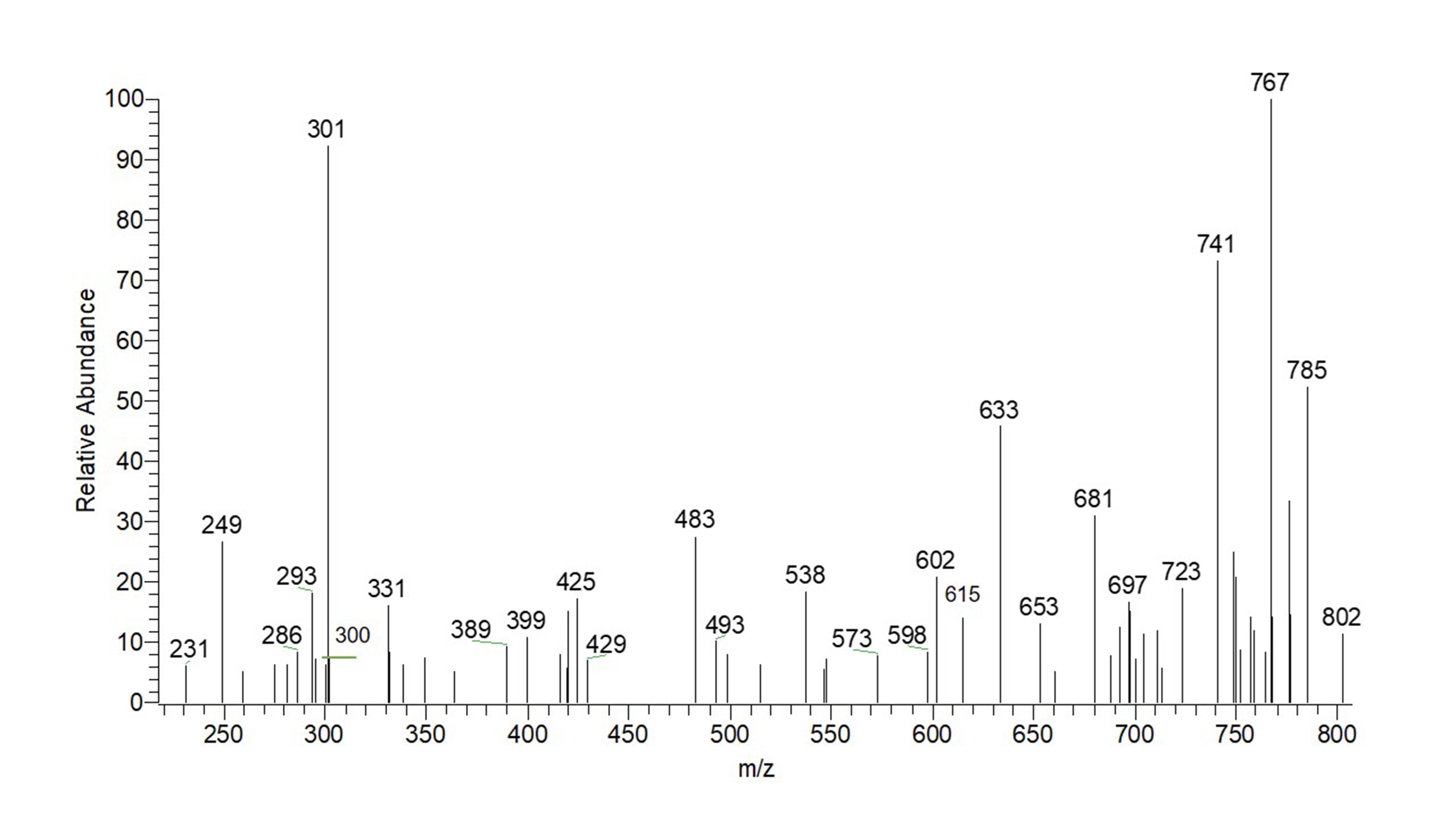

Supplement: Supplementary file 58 — Figure S58: Product ion mass spectrum of the ion of mz 785. [file JMS-60-e5173-s019.jpg]

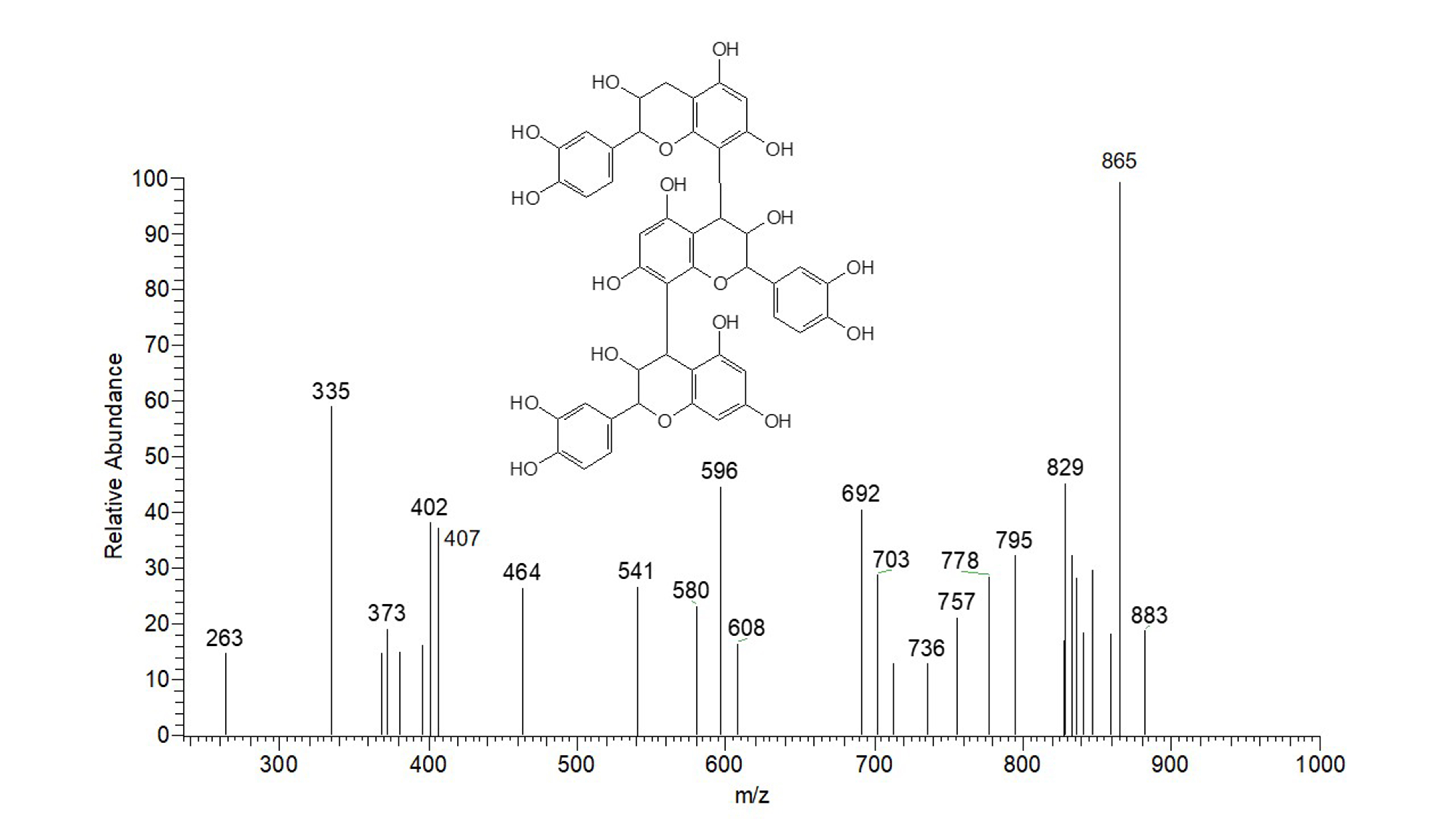

Supplement: Supplementary file 59 — Figure S59: Product ion mass spectrum of the ion of mz 865. [file JMS-60-e5173-s053.jpg]

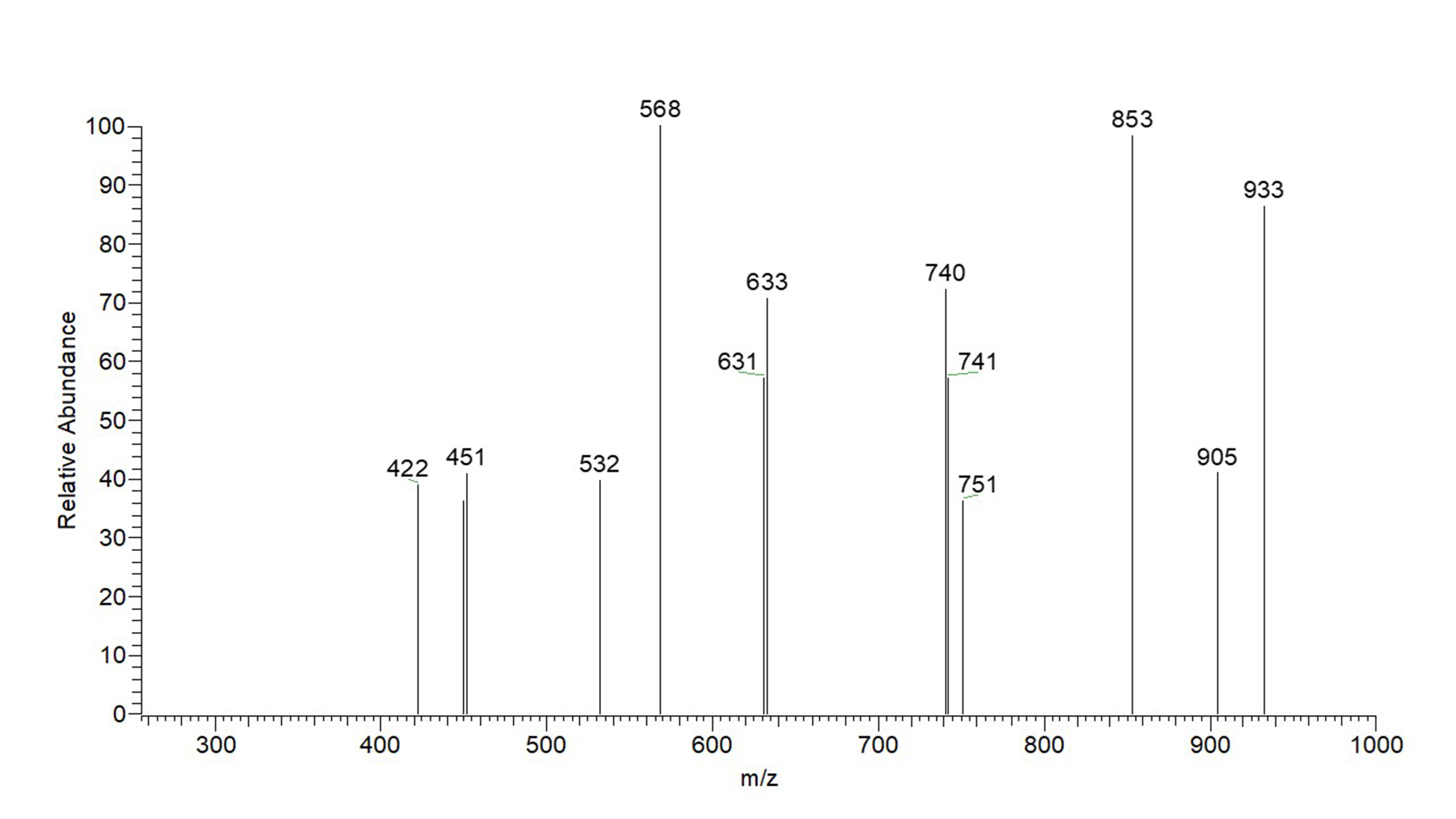

Supplement: Supplementary file 60 — Figure S60: Product ion mass spectrum of the ion of mz 933. [file JMS-60-e5173-s054.jpg]

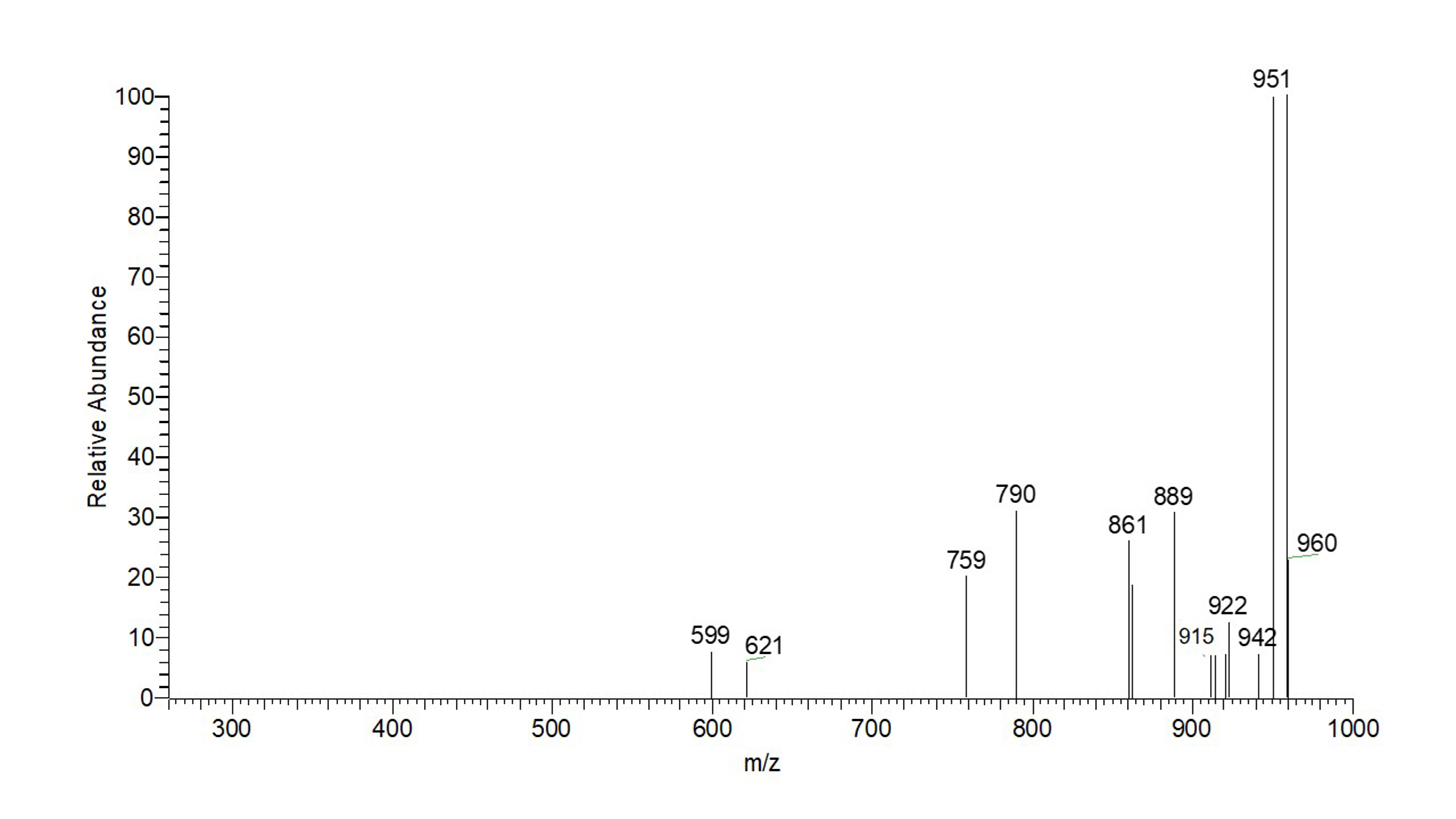

Supplement: Supplementary file 61 — Figure S61: Product ion mass spectrum of the ion of mz 951. [file JMS-60-e5173-s020.jpg]
